# Semaphorin 7A interacts with nuclear factor NF-kappa-B p105 via integrin β1 and mediates inflammation

**DOI:** 10.1186/s12964-022-01024-w

**Published:** 2023-01-30

**Authors:** Xuan Li, Wanlu Xie, Qiong Pan, Xiaoxun Zhang, Liangjun Zhang, Nan Zhao, Qiaoling Xie, Jingjing Ding, Jin Chai

**Affiliations:** 1 Department of Gastroenterology, The First Affiliated Hospital (Southwest Hospital) to Third Military Medical University (Army Medical University), Chongqing, 400038 China; 2 Institute of Digestive Diseases of PLA, The First Affiliated Hospital (Southwest Hospital) to Third Military Medical University (Army Medical University), Chongqing, 400038 China; 3 Cholestatic Liver Diseases Center, The First Affiliated Hospital (Southwest Hospital) to Third Military Medical University (Army Medical University), Chongqing, 400038 China; 4 Center for Metabolic Associated Fatty Liver Disease, The First Affiliated Hospital (Southwest Hospital) to Third Military Medical University (Army Medical University), Chongqing, 400038 China

**Keywords:** Semaphorin7a, NF-kappa-B p105, NF-kappa-B p65, Integrin β1, Inflammation

## Abstract

Semaphorin7a (SEMA7A), a membrane-anchored member of the semaphorin protein family, could be involved in a diverse range of immune responses via its receptor integrin β1. Recently, we reported that the *SEMA7A*^R148W^ mutation (a gain-of-function mutation, *Sema7a*^R145W^ in mice) is a risk factor for progressive familial intrahepatic cholestasis and nonalcoholic fatty liver disease via upregulated membrane localization. In this study, we demonstrated that integrin β1 is a membrane receptor for nuclear factor NF-kappa-B p105 (NF-κB p105) and a critical mediator of inflammation. Integrin β1 could interact with the C-terminal domain of NF-κB p105 to promote p50 generation and stimulate the NF-κB p50/p65 signalling pathway, upregulate TNF-α and IL-1β levels, and subsequently render hepatocytes more susceptible to inflammation. The induction of integrin β1 depends on elevated Sema7a membrane localization. Moreover, we revealed elevated levels of Sema7a^WT^ (SEMA7A^WT^) in hepatocellular carcinoma (HCC) patients and an HCC mouse model. In line with our findings, the NF-κB p50/p65 pathway could also be activated by high Sema7a expression and repressed by integrin β1 silencing. In conclusion, our findings suggest that the *Sema7a*^R145W^ (*SEMA7A*^R148W^) mutation and high Sema7a^WT^ (SEMA7A^WT^) expression both activate the NF-κB p50/p65 pathway via integrin β1 and play a crucial role in inflammatory responses.

**Video Abstract**

**Supplementary Information:**

The online version contains supplementary material available at 10.1186/s12964-022-01024-w.

## Background

Semaphorins are a protein family characterized by a conserved extracellular amino‐terminal SEMA domain [1]. Semaphorin 7A (SEMA7A) is classified as the VII semaphorin due to its unique glycosylphosphatidylinositol (GPI) anchorage on the cell membrane. SEMA7A plays an essential role in both the nervous and immune systems, mainly mediated by its receptors, including integrins and plexins [2–7]. Accumulating evidence has shown that the *SEMA7A*^R148W^ mutation (*Sema7a*^R145W^ in mice) is a gain-of-function mutation and a risk factor involved in progressive familial intrahepatic cholestasis (PFIC) and non-alcoholic fatty liver disease (NAFLD). Pan et al. demonstrated that the *SEMA7A*^R148W^ mutation could disturb bile acid transport by reducing bile salt export pump and multidrug resistance-associated protein-2 expression, resulting in PFIC development [8]. Zhao et al. reported that the mutation could promote hepatocyte lipid accumulation via integrin β1 and aggravate NAFLD progression [9]. Inflammation is an important immune response involved in the occurrence and development of cholestasis and fatty liver disease [10–12]. Therefore, the molecular mechanism of the *SEMA7A*^R148W^ mutation in liver inflammation needs to be studied and clearly described.

Inflammation underlies a broad range of physiological and pathological processes and is closely associated with cholestasis and NAFLD [11–13]. A classic inflammatory pathway consists of inducers, sensors, mediators and effectors [14]. For example, in chronic liver disease such as NAFLD and cholestasis, endogenous signals and products can induce the production of numerous inflammatory mediators, such as proinflammatory chemokines and cytokines. Immunoinflammatory responses to those chemokines and cytokines, especially tumour necrosis factor α (TNF-α) and interleukin-1β (IL-1β), are almost ubiquitous [15, 16]. The effectors of the inflammatory response are liver tissues and cells, the functional states of which are specifically affected by inflammatory mediators. Under sustained hepatic inflammation circumstances, the liver parenchyma will be damaged and generally exhibit a higher cancer incidence [17–20]. Thus, the exploration of the molecular mechanisms of chronic inflammation could represent attractive therapeutic targets with a reduced risk of tumour progression.

Proinflammatory cytokines such as TNF-α and IL-1β are principally encoded by target genes of the NF-κB p50/p65 canonical pathway [21, 22]. Nuclear factor NF-kappa-B (NF-κB), an important transcription factor in the inflammatory response, plays an essential role in chronic liver disease [23, 24]. The NF-κB protein family consists of p50, p52, p65 (also known as Rel-A), C-Rel and Rel-B. NF-κB p50 mostly arises from the precursor protein NF-κB p105 (*NFKB1*) and forms heterodimers with p65 [25–27]. In unstimulated cells, NF-κB p50/p65 heterodimers are mainly bound to inhibitory proteins such as inhibitor of κB alpha (IκBα). When cells are stimulated by exogenous or endogenous signals, IκBα is phosphorylated by the cytosolic IKK holoenzyme at two different serine residues (Ser32 and Ser36) and subsequently degraded through a ubiquitin-dependent pathway [28]. After that, NF-κB p50 and p65 are released from the inhibitory complex and phosphorylated immediately. After all these processes, NF-κB p50/p65 enters the nucleus, binds to DNA and activates gene transcription.

In this study, in a genetic *Sema7a*^R145W^ homozygous mouse model, we first identified that integrin β1 could interact with the C-terminal domain of NF-κB p105 to promote p50 generation and stimulate the NF-κB p50/p65 canonical pathway, consequently rendering hepatocytes more susceptible to inflammation. Furthermore, we discovered that the level of Sema7a^WT^ (SEMA7A^WT^) is increased in hepatocellular carcinoma (HCC) patients, HCC mouse model and HCC cell lines. The ectopic expression of SEMA7A^WT^ in vitro also activated NF-κB p50/p65 and upregulated proinflammation cytokines. In addition, we observed ascending cell migration and proliferation in SEMA7A^WT^ transfected HepG2 cells. Together, these results suggest that both the *Sema7a*^R145W^ (*SEMA7A*^R148W^) mutation and high Sema7a^WT^ (SEMA7A^WT^) expression displayed similar proinflammatory signalling, and Sema7a^WT^ (SEMA7A^WT^) is correlated with HCC to some extent.

## Methods

### Animals

All protocols in this study were approved by the Animal Care and Use Committee of the Army Medical University, China. All mice were housed in cages [5 mice per cage] with standard food and water and maintained in an appropriate temperature and light/dark cycle (12 h/12 h). Male homozygous *Sema7a*^R145W^ (c.433C > T) mutant mice (HO) and their wild-type (WT) littermates (aged from 8 to 10 weeks) were used in this study. Homozygous *Sema7a*^R145W^ mutant mice (C57BL/6 J background) were designed and generated by Shanghai Model Organisms Center via the Cas9-targeted single guide RNA of 5′ *ATGCCCGGAAGCCCAGCTGCTGG* 3′. The generation protocol for *Sema7a*^R145W^ mutant mice has been described previously [8, 9]. Liver samples were collected from wild-type (n = 4) and *Sema7a*^R145W^ homozygous mice (n = 5). Diethylnitrosamine (DEN)/CCl4 was used to induce HCC in C57BL/6 J mice (n = 5). The HCC group was intraperitoneally (i.p.) injected with DEN (Sigma‒Aldrich, St. Louis, MO) in physiological saline (25 μg/g) at the age of 3 weeks. At the age of 5 weeks, the mice were treated with 2.5 μl/g body weight CCl4 (Sigma‒Aldrich, St. Louis, MO) diluted (1:9) in corn oil by i.p. injection once a week for 22 weeks. The vehicle control group was i.p. injected with matching volumes of corn oil following the same method. Mice were sacrificed at 27 weeks of age. Liver samples were collected from wild-type (n = 5) and hepatocellular carcinoma mice (n = 5).

### Histological analysis

Livers were perfused with saline solution for a few minutes and then excised and placed in 10% neutral buffered formalin immediately. Haematoxylin–eosin (H&E)-staining followed the protocol from the H&E Stain Kit (Beijing Solarbio Science & Technology Co., Ltd). H&E-stained Sections (5 µm thick) were used to determine liver inflammation. Liver inflammation was scored by two expert pathologists according to the Scheuer Scoring System [29]. For liver tissue immunofluorescence (IF), 5 µm thick sections were incubated with the primary p-NF-κB p65 Ser529 antibody (Affinity, AF3388, 1:200) overnight at 4 °C. Subsequently, sections were incubated with Alexa Fluor555 secondary antibodies (1:200, Cell Signaling Technology) for 1 h at 37 °C.

### Western blotting

Total liver tissue homogenates and whole cell lysates were used for Western blotting to determine (i) protein levels of proinflammation cytokines: TNF-α (Proteintech, 60291-1-Ig, 1:1000) and IL-1β (Abcam, ab234437, 1:1000); (ii) the activation state of NF-κB p50/p65 signals including NF-κB p105 (Proteintech, 66992-1-Ig, 1:1000), p-NF-κB p50 (Abclonal, AP0125, 1:1000)/NF-κB p50 (Proteintech, 66992-1-Ig, 1:1000), p-NF-κB p65 (Affinity,AF3388, 1:2000)/NF-κB p65 (Abclonal, A19653, 1:500), and p-IκB (Affinity,AF2002, 1:2000)/IκB (Affinity,AF5002, 1:2000); and (iii) protein levels of Sema7a (Proteintech, 67397-1-Ig, 1:1000) and integrin β1 (Abcam, ab183666, 1:5000). Quantification of Western blotting results was determined by densitometric scanning using ImageJ software. Raw data of Western blotting results were included in Additional file 4.

### Real-time quantitative RT–PCR

Total RNA was extracted from frozen mouse liver tissues using TRIzol reagent (Invitrogen, USA). cDNA was synthesized from 1 µg RNA of each sample using PrimeScript RT Master Mix (Takara, Japan). Quantitative RT–PCR analysis was performed using the SYBR Green kit (Takara, Japan) and specific primers. The amplification conditions were 90 s at 95 °C, followed by 39 cycles of 5 s at 95 °C and 30 s at 65 °C. Glyceraldehyde-3-phosphate dehydrogenase (*GAPDH*) was used for normalization. Relative quantification of the gene expression was calculated by the 2^−ΔΔCt^ method. The sequences of specific primers are listed in Additional file 1: Table S2.

### Plasmid and siRNA construction

pcDNA3.1 plasmids with (i) C-terminal tagged HIS for full-length NF-κB p105 or truncated NF-κB p55; (ii) C-terminal tagged FLAG for full-length integrin β1; and (iii) full-length SEMA7A^WT^ were all generated by Youbio Biological Technology Co., Ltd. The siRNA sequences for *ITGB1* (integrin β1) were F: 5′-*CUGUAAGUG CAAUUGUCAATT*-3′ and R: 5′-*UUGACAAUUGCACUUACAGTT*-3′, as described previously [9]. Recombinant DNA sequences were confirmed by sequencing and the expression of correctly sized proteins was confirmed by Western blotting. The cultured human hepatoma HepG2 cells and human HEK 293 T cells were transfected with the above plasmids, integrin β1-specific siRNA, or control scramble siRNA using the GeneTwin Transfection reagent (Beijing Biomed Gene Technology Co., TG101) or the HiPerFect Transfection reagent (QIAGEN, #301,705), according to the manufacturer’s instructions.

### Collection and transfection of primary mouse hepatocytes, HEK293 cells and HepG2 cells

Primary mouse hepatocytes were isolated from *Sema7a*^R145W^ homozygous and WT mouse livers by collagenase perfusion and cultured in William’s E medium (Gibco) with 5% foetal bovine serum (FBS) as described previously [8, 9, 30, 31]. HEK293 and HepG2 cells were cultured in DMEM (Cytiva) with 10% FBS. Cell cultures were kept in a controlled incubator at 37 °C and 5% CO2.

For siRNA knockdown, cultured primary mouse hepatocytes seeded on 6-well plates were transfected with siRNA for *ITGB1* silencing using HiPerFect Transfection Reagent (QIAGEN, #301,705) when the cell density was approximately 40–50%. After 72 h, the interference effect was detected by Western blotting with an integrin β1 antibody. Additionally, for plasmid transfection, HEK293 cells and HepG2 cells (also seeded on 6-well plates for 40% cell density) were transfected with the corresponding plasmids using GeneTwin Transfection reagent (Beijing Biomed Gene Technology Co., TG101). HEK293 cells were cotransfected with pcDNA3.1-HIS-NF-κB p105 and pcDNA3.1-FLAG-integrin β1 plasmid or with pcDNA3.1-HIS-NF-κB p55 and pcDNA3.1-FLAG-integrin β1 plasmid for 24 h. HepG2 cells were transfected with the pEGFP-SEMA7A^WT^ plasmid for 24 h. The expression level was detected by Western blotting using the corresponding antibody.

### Coimmunoprecipitations (co-IP)

Liver tissues and cells were lysed in ice RIPA buffer (Sigma‒Aldrich, St. Louis, MO, R0278) for at least 30 min and then centrifuged to remove cell debris. For each immunoprecipitation, 500 µg of the sample was incubated with 50 μl of Protein A -agarose beads (Invitrogen, #20333) and 2 μg of antibody on a rocking platform overnight at 4 °C. The protein complexes were washed three times with cold PBS and resuspended in 2 × SDS PAGE protein loading buffer (BOSTER Biological Technology Co., AR0131-20). The immunoprecipitated proteins were eluted from the beads by incubation at 55 °C for 5 min. Immunoblotting was used to detect immunoprecipitates after separation by SDS‒PAGE.

### Wound healing assay and CCK-8

For the wound healing assay, cells were seeded into 6-well plates at 90% cell density and scratched gently with a 200 μL pipette tip. The cells were washed twice with PBS to remove detached cells and the remaining cells were incubated in medium without FBS. The scratch areas were photographed at 0 h, 24 h and 48 h. Experiments were performed in triplicate in each group. Cell proliferation was assessed by a cell counting kit-8 (CCK-8) assay according to the standard protocol (Beijing Biomed Gene Technology Co., PA133-02). The absorbances were measured at a wavelength of 450 nm on a universal microplate reader, and the calculation formula of the cell proliferation rate was ((A450 sample − background)/(A 450 control–background)) × 100%. All tests were performed three times in quadruplicate.

### Statistical analyses

Data were analysed using the independent-samples Student’s t test or the Mann–Whitney U test, as appropriate. *P*-values < 0.05 were considered statistically significant. Descriptive statistics were calculated with SPSS software (PASW Statistics 18, IBM; SPSS, Chicago, IL, USA).

## Results

### *Sema7a* gain-of-function mutation promotes inflammation in mouse liver

**Fig. 1:**
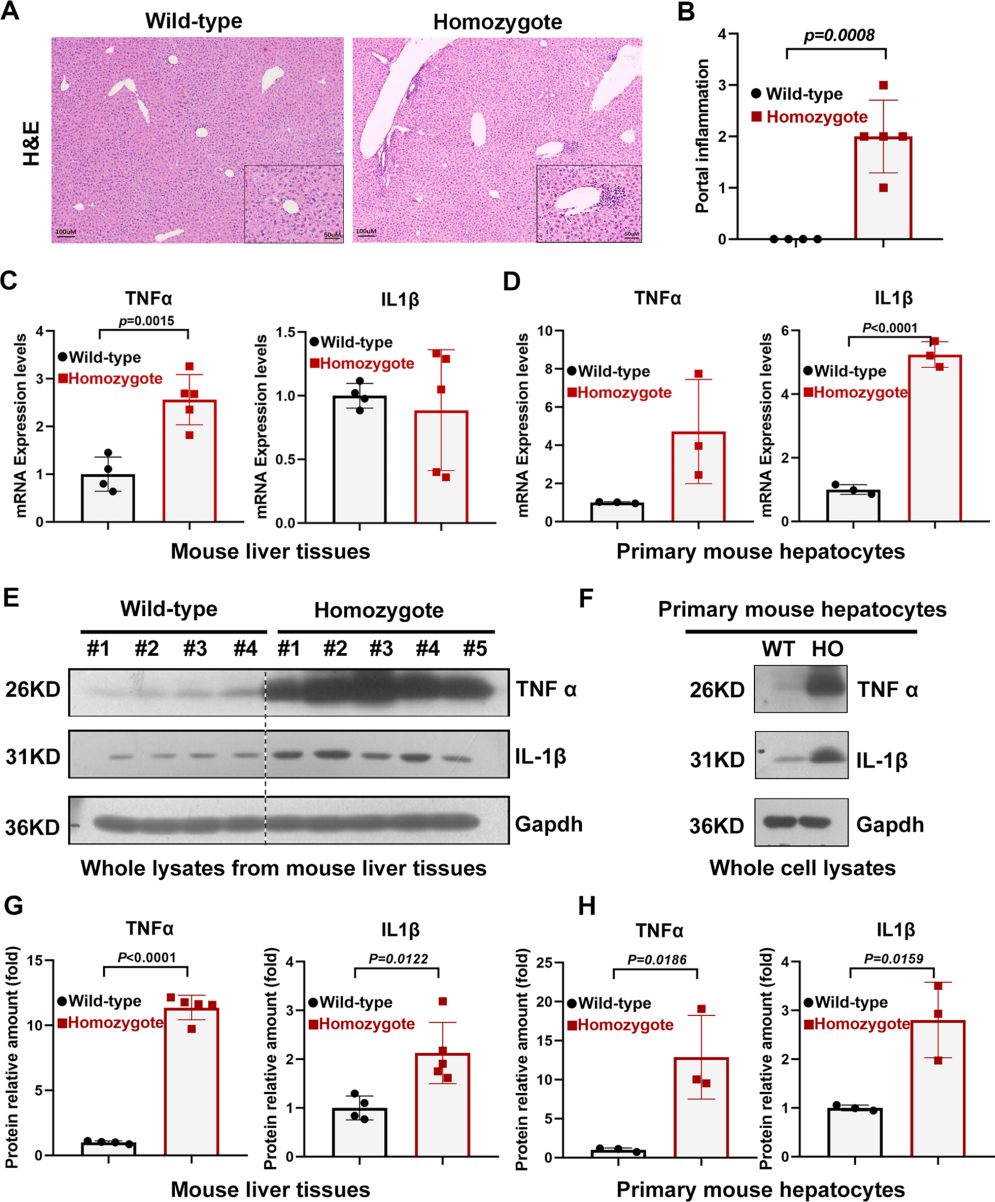
*Sema7a*^R145W^ homozygous mice displayed inflammation accompanied by elevated inflammatory cytokines. **a** Representative images of H&E staining in wild-type and *Sema7a*^R145W^ homozygous male mice at 8 weeks of age. **b** Analysis of portal inflammation infiltration in H&E-stained liver sections of wild-type (n = 4) and *Sema7a*^R145W^ homozygous male mice (n = 5). **c** The relative levels of mRNA transcripts of TNFα and IL1β in liver tissues of wild-type (n = 4) and *Sema7a*^R145W^ homozygous male mice (n = 5) and **d** in primary hepatocytes from wild-type (n = 3) and *Sema7a*^R145W^ homozygous (n = 3) male mice. **e**, **g** Western blotting analysis of the relative levels of TNFα and IL1β protein expression in the liver of wild-type (n = 4) and *Sema7a*^R145W^ homozygous male mice (n = 5) and **f**, **h** in primary hepatocytes from wild-type (n = 3) and *Sema7a*^R145W^ homozygous (n = 3) male mice. The data were analysed by the independent-samples Student’s t test. * means *p* < 0.05 versus wild-type mice

To investigate whether the *SEMA7A*^R148W^ mutation contributes to liver inflammation, we first generated a *sema7a*^R145W^ homozygous mouse model and performed histological staining of the livers of the animals using H&E staining. The H&E staining results showed an increased amount of inflammatory infiltration in liver sections of *Sema7a*^R145W^ homozygous mice compared to those from the WT group, particularly in the hepatic portal areas (Fig. 1A&B). Since proinflammatory cytokines are key mediators of inflammation, we further investigated the levels of proinflammatory cytokines in liver tissues and primary mouse hepatocytes. We observed elevated mRNA levels of TNF-α in both liver tissues and primary mouse hepatocytes of homozygous mice (Fig. 1C&D). Finally, increased protein levels of TNF-α and IL-1β in liver tissues and primary mouse hepatocytes of *Sema7a*^R145W^ homozygous mice were observed by Western blotting (Fig. 1E-H), which was basically consistent with the qPCR results. Together, these results indicate that the *Sema7a*^R145W^ (*SEMA7A*^R148W^) mutation is related to liver inflammation and is most likely to occur in hepatocytes.

### The NF-κB p50/p65 signalling pathway is activated in ***Sema7a***^R145W^ mouse liver

**Fig. 2:**
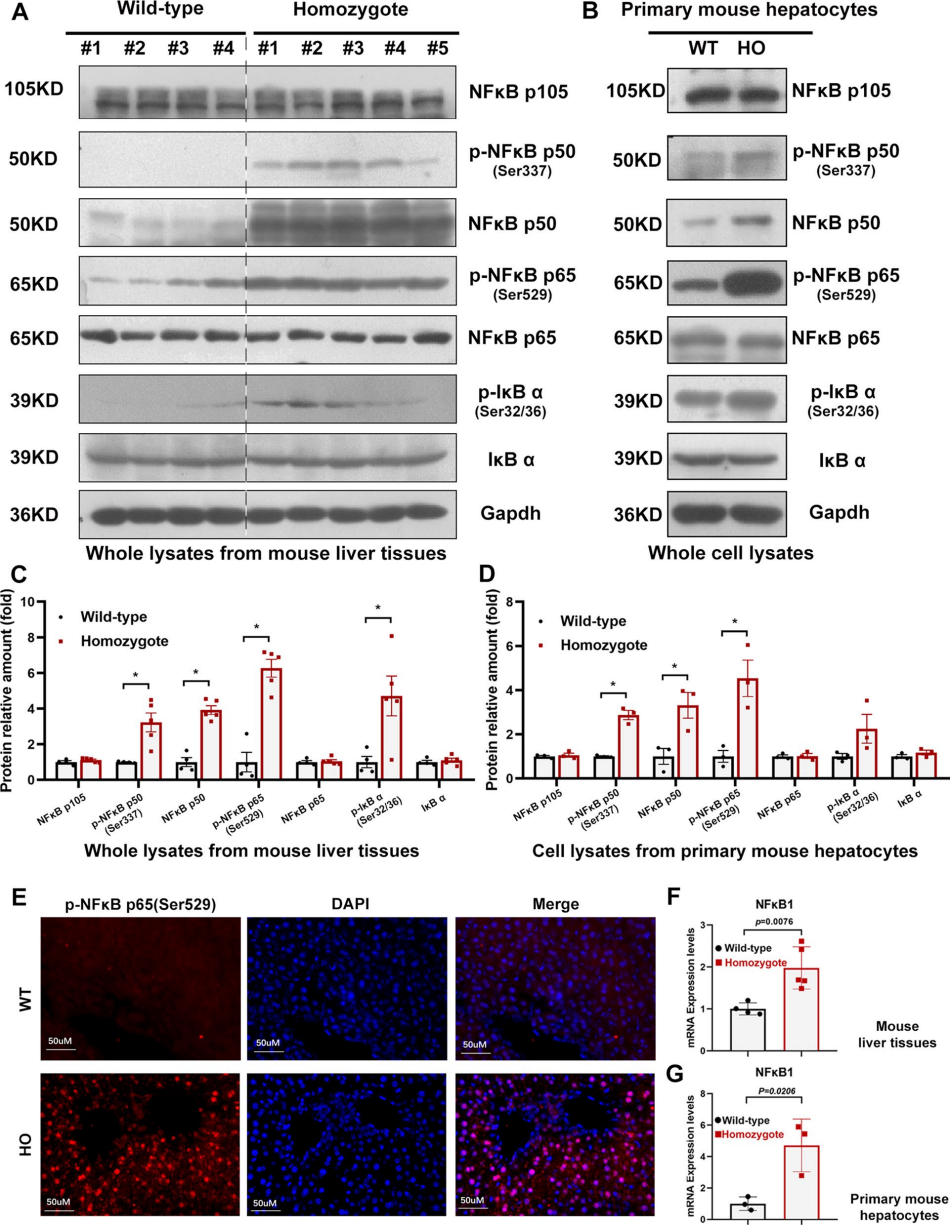
The NF-κB pathway is activated in *Sema7a*^R145W^ mouse liver and primary mouse hepatocytes. **a–c** Western blotting analysis of the relative protein levels of NF-κB p105, p- NF-κB p50/NF-κB p50, p- NF-κB p65/NF-κB p65 and p-IκB/IκB in the livers of wild-type (n = 4) and *Sema7a*^R145W^ homozygous male mice (n = 5) and **b**, **d** in primary hepatocytes from wild-type (n = 3) and *Sema7a*^R145W^ homozygous (n = 3) male mice. Phosphorylation levels were measured by the phosphor/total protein ratio. **e** Representative IF staining showing p-NF-κB p65 Ser529 (red) and DAPI (blue). Scale bars: 50 μm in liver sections from wild-type and *Sema7a*^R145W^ homozygous male mice. **f** The relative levels of mRNA transcripts of the genes for NF-κB p105 in wild-type (n = 4) and *Sema7a*^R145W^ homozygous male mice (n = 5) and **g** in primary hepatocytes from wild-type (n = 3) and *Sema7a*^R145W^ homozygous (n = 3) male mice. The data were analysed by the independent-samples Student’s t test. * means *p* < 0.05 versus wild-type mice

We next investigated the underlying mechanism of hepatic inflammation promoted by the *Sema7a*^R145W^ (*SEMA7A*^R148W^) mutation. Previous studies indicated that TNF-α and IL-1β could be notably regulated by NF-κB p50/p65 signalling [21, 32, 33]. Hence, we evaluated the protein expression of NF-κB subunits, IκBα and their corresponding phosphorylated forms, which were strongly linked to the activation of the NF-κB p50/p65 canonical pathway using Western blotting. We found that the levels of the phosphorylated forms of p-p50 (Ser337), p-p65 (Ser529), and p-IκBα (Ser32/36) were significantly induced by the *Sema7a*^R145W^ mutation, suggesting that the *Sema7a*^R145W^ mutation might play a critical role in inflammation via the NF-κB p50/p65 pathway (Fig. 2A&C). Similarly, we observed a significant increase in the phosphorylation levels of NF-κB subunits and IκBα in *Sema7a*^R145W^ primary mouse hepatocytes (Fig. 2B&D). Numerous references have supported that NF-κB p50 and p65 immediately form heterodimers once they have been phosphorylated and transferred into the nucleus for gene activation [25–27]. Thus, the immunofluorescence result of phosphorylated NF-κB p65 was used to prove the nuclear translocation of NF-κB p50/p65 (Fig. 2E). Interestingly, we noticed that the levels of NF-κB p50 were remarkably high in both *Sema7a*^R145W^ mouse liver tissues and primary mouse hepatocytes, indicating that numerous p105 might have been processed into p50. Because the p50/p65 heterodimers is the mainly targets of NF-κB canonical pathway and the NF-κB p50 subunit is of vital importance when heterodimers bound to DNA, the function of NF-κB p105 and p50 in the *Sema7a*^R145W^ mutation should be further demonstrated in the subsequent studies.

### Integrin β1 interacts with NF-κB p105, promotes NF-κB p105 into p50 and activates the downstream pathway

**Fig. 3:**
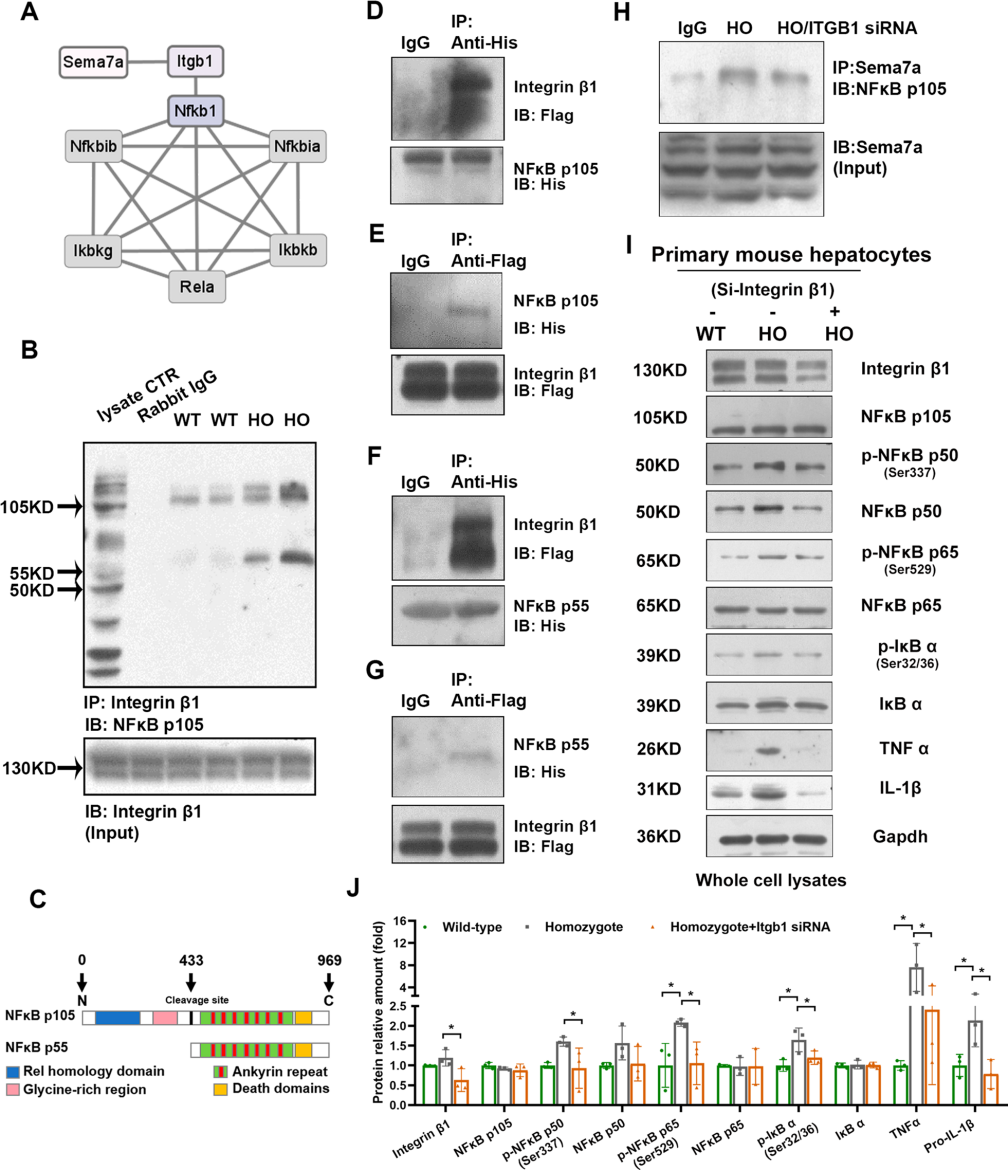
Integrin β1 binds to NF-κB p105 and promotes the NF-κB p50/65 pathway. **a** The protein‒protein interaction network of the Sema7a, integrin β1 and NF-κB protein families was predicted by the Retrieval of Interacting Genes Database. **b** Coimmunoprecipitation analysis of protein interactions among integrin β1 and NF-κB p105 in liver tissues from 8-week-old male wild-type and *Sema7a*.^R145W^ homozygous mice. The higher band is NF-κB p105 (105 kDa) and the lower band is NF-κB p55 (55 kDa). **c** Schematic showing the primary structure and truncation of NF-κB p105. **d** and** e** Coimmunoprecipitation analysis of His-tagged NF-κB p105 and Flag-tagged integrin β1 in HEK293 cells. **f**, **g** Co-immunoprecipitation analysis of His-tagged NF-κB p55 and Flag-tagged integrin β1 in HEK293 cells. **h** Coimmunoprecipitation analysis of the protein interaction between Sema7a and NF-κB p105 in primary mouse hepatocytes after integrin β1 silencing (n = 3). **i**, **j** Western blotting analysis of the relative levels of integrin β1, NF-κB p105, p- NF-κB p50/NF-κB p50, p- NF-κB p65/NF-κB p65, p-IκB/IκB, TNFα and IL1β protein expression in primary hepatocytes following integrin β1 silencing (n = 3). Phosphorylation levels were measured by the phosphor/total protein ratio. The difference among the groups was determined by one-way ANOVA with Tukey’s post hoc tests or by Kruskal‒Wallis test with Dunn’s post hoc test analysis. *Means *p* < 0.05

We wondered if there is a correlation between membrane Sema7a^R145W^ and increased NF-κB p105 processing in homozygous *Sema7a*^R148W^ mutant mice. Since the membrane localization of Sema7a^R145W^ (SEMA7A^R148W^) and integrin β1 were thought to increase in our previous study [9], we first retrieved protein–protein interaction (PPI) information among SEMA7A, integrin β1 and the NF-κB protein family using the Search Tool for the Retrieval of Interacting Genes Database (STRING) (https://www.string-db.org/). The PPI network shows that integrin β1 directly interacts with NF-κB p105 and mediates the interaction between Sema7a and NF-κB p105 (Fig. 3A). To confirm this, we used an anti-integrin β1 antibody to precipitate NF-κB p105/p50 in liver tissues from both WT and *Sema7a*^R145W^ mice and then used an NF-κB p105 antibody that specifically binds to the C-terminus for immunoprecipitation detection. As expected, integrin β1 bound to NF-κB p105 in mouse livers. The results demonstrated that integrin β1 could interact with NF-κB p105 (105 kDa, the higher bands) (Fig. 3B). Meanwhile, there was another band in the lower location. According to the kDa number, the lower bands are probably NF-κB p55 (55 kDa). NF-κB p50 (~ 50 kDa) is the N-terminal processed product of p105 (~ 105 kDa), and a massive 55 kDa truncation protein (named p55 in the study) might be generated when p50 is largely generated from full-length p105 (Fig. 3C). Thus, our analysis suggests that the lower bands are NF-κB p55 (55 kDa). According to these results, we speculated that integrin β1 could bind to the C-terminus of p105 (including 7 ankyrin repeats and a death domain) and promote p50 generation. To further discuss this assumption, HEK293 cells were cotransfected with flag-integrin β1 plasmid and His-NF-κB p105 plasmid to overexpress integrin β1 and NF-κB p105. The co-IP and Western blotting analyses validated the interaction between integrin β1 and NF-κB p105 (Fig. 3D&E), indicating that there is probably a new kind of protein complex that consists of Sema7a, integrin β1 and NF-κB p105. Next, we constructed a p55 truncation plasmid and then ectopically expressed both flag-integrin β1 and His-NF-κB p55 plasmids in HEK293 cells. Co-IP and Western blotting further determined that integrin β1 mainly binds to the structure of p55 (Fig. 3F&G). Moreover, the exogenous co-IP results in primary hepatocytes showed that NF-κB p105 was detected in the immunoprecipitated proteins of SEMA7A (Fig. 3H). When integrin β1 was inhibited, this interaction between Sema7a^R145W^ and NF-κB p105 subsequently decreased. However, because of the insufficient inhibition efficiency (~ 50–60%), there still existed a slight protein band. Overall, Fig. 3H demonstrates that Sema7a^R145W^ binds to NF-κB p105 and that this binding may be primarily mediated by integrin β1. Since the effect of integrin β1 on p50 generation and NF-κB p65 signalling pathway activation is unknown, we used siRNA targeting *ITGB1* (integrin β1) and detected its relative protein expression by Western blotting analyses. We found that the phosphorylation levels of NF-κb subunits, p-IκBα (Ser32/36), TNF-α and IL-1β significantly decreased after si-*ITGB1* treatment in *Sema7a*^R145W^ primary mouse hepatocytes (Fig. 3I&J). Taken together, our data revealed a new protein complex that was formed by Sema7a, integrin β1 and NF-κB p105. Moreover, integrin β1 binds to p105, promoting p50 generation and NF-κB p65 signalling pathway activation. The activated NF-κB p50/p65 signalling pathway can then stimulate TNF-α and IL-1β synthesis and secretion, leading to excessive accumulation of hepatic inflammation.

### Sema7a/integrin β1/NF-κB p105 signalling pathway is activated in hepatocellular carcinoma

**Fig. 4:**
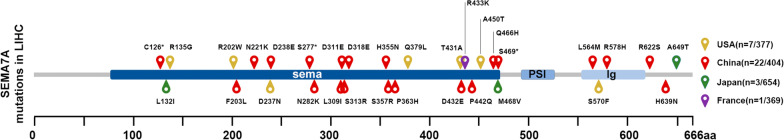
*SEMA7A* gene mutation in 1804 hepatocellular carcinoma patients from ICGC database [https://dcc.icgc.org/]

**Fig. 5:**
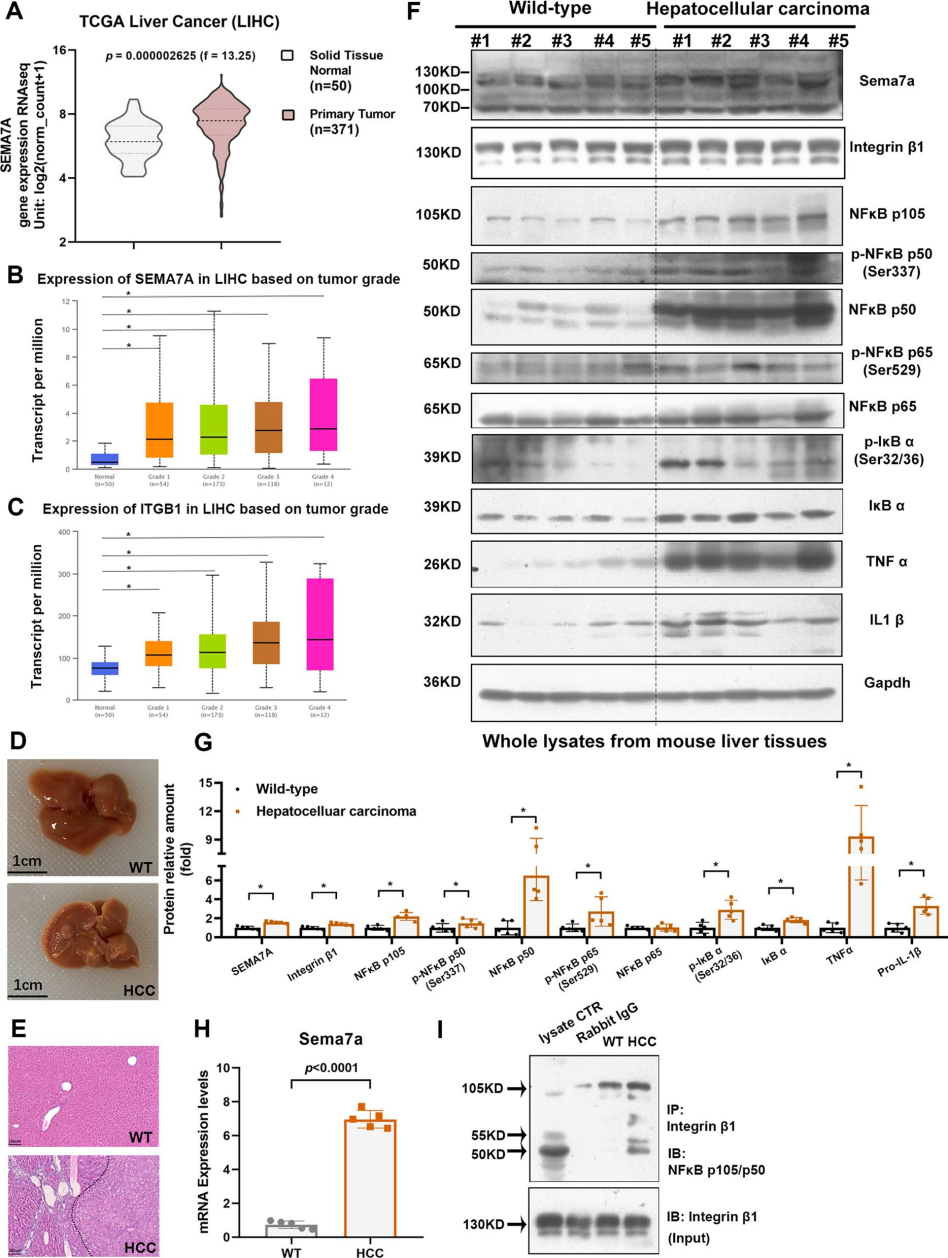
SEMA7A is elevated in hepatocellular carcinoma. **a** The mRNA level of SEMA7A in liver hepatocellular carcinoma from TCGA. **b** and **c** The mRNA levels of SEMA7A and integrin β1 in four tumour grades. **d** Characteristic images of liver tissue from wild-type and hepatocellular carcinoma mice. **e** Representative images of H&E staining in wild-type and hepatocellular carcinoma. **f**, **g** Western blotting analysis of the relative levels of Sema7a, integrin β1, NF-κB p105, p- NF-κB p50/NF-κB p50, p- NF-κB p65/NF-κB p65, p-IκB/IκB, TNFα and IL1β protein expression in the livers of wild-type (n = 5) and hepatocellular carcinoma mice (n = 5). **h** The relative levels of mRNA transcripts of the genes for SEMA7A in wild-type (n = 5) and hepatocellular carcinoma mice (n = 5). **i** Coimmunoprecipitation analysis of protein interactions among integrin β1 and NF-κB p105 in liver tissues from wild-type and hepatocellular carcinoma mice. The data were analysed by the independent-samples Student’s t test. *Means *p* < 0.05 versus wild-type mice

The link between chronic inflammation and tumorigenesis has been described in numerous studies. The inflammatory response plays crucial roles at different stages of tumour development, including initiation, promotion, and invasion. [20, 34]. To explore whether the *SEMA7A*^R148W^ mutation is a risk factor in HCC, we investigated the *SEMA7A* mutation frequency in a total of 1804 HCC patients included in four cohorts from the International Cancer Genome Consortium (ICGC) database[35]. These four cohorts were classified by different countries; the *SEMA7A* mutation rate is 1.9% (7/377 patients) in the U.S., 5.4% in China (22/404 patients), 0.5% in Japan (3/654 patients) and 0.3% in France (1/369 patients) (Fig. 4 and Additional file 1: Table S1). However, the *SEMA7A*^R148W^ mutation was not found in HCC patients. Interestingly, we noticed that the mRNA levels of SEMA7A^WT^ and integrin β1 were significantly increased in the tumour group (n = 371) compared to the normal group (n = 50) (Fig. 5A-C). The Figs. 5B and 5C show that the gene expression of SEMA7A^WT^ and integrin β1 in different tumour grades was significantly higher than that in the normal group. However, there were no significant differences among the four tumour grades. We therefore constructed a primary hepatocellular cancer mouse model (Fig. 5D&E and Additional file 2: Fig. S1) to investigate the role of Sema7a^WT^ (SEMA7A^WT^) in HCC. Liver H&E staining showed elevated inflammatory infiltration in liver sections of model animals (Fig. 5E). Liver tissues were used to examine the protein and mRNA levels of Sema7a^WT^ via Western blotting and qPCR, respectively (Fig. 5F-H). These results are consistent with HCC data analysis in the TCGA [http://cancergenome.nih.gov/] and UALCAN platform [36]. Compared to WT mice, the NF-κB p50/p65 signalling pathway was activated and proinflammatory cytokines were upregulated in the HCC mouse group (Fig. 5F&G), which is consistent with previous studies [18, 21, 37]. Additionally, co-IP results displayed an increased interaction between integrin β1 and NF-κB p105 in HCC mouse livers (Fig. 5I).

**Fig. 6:**
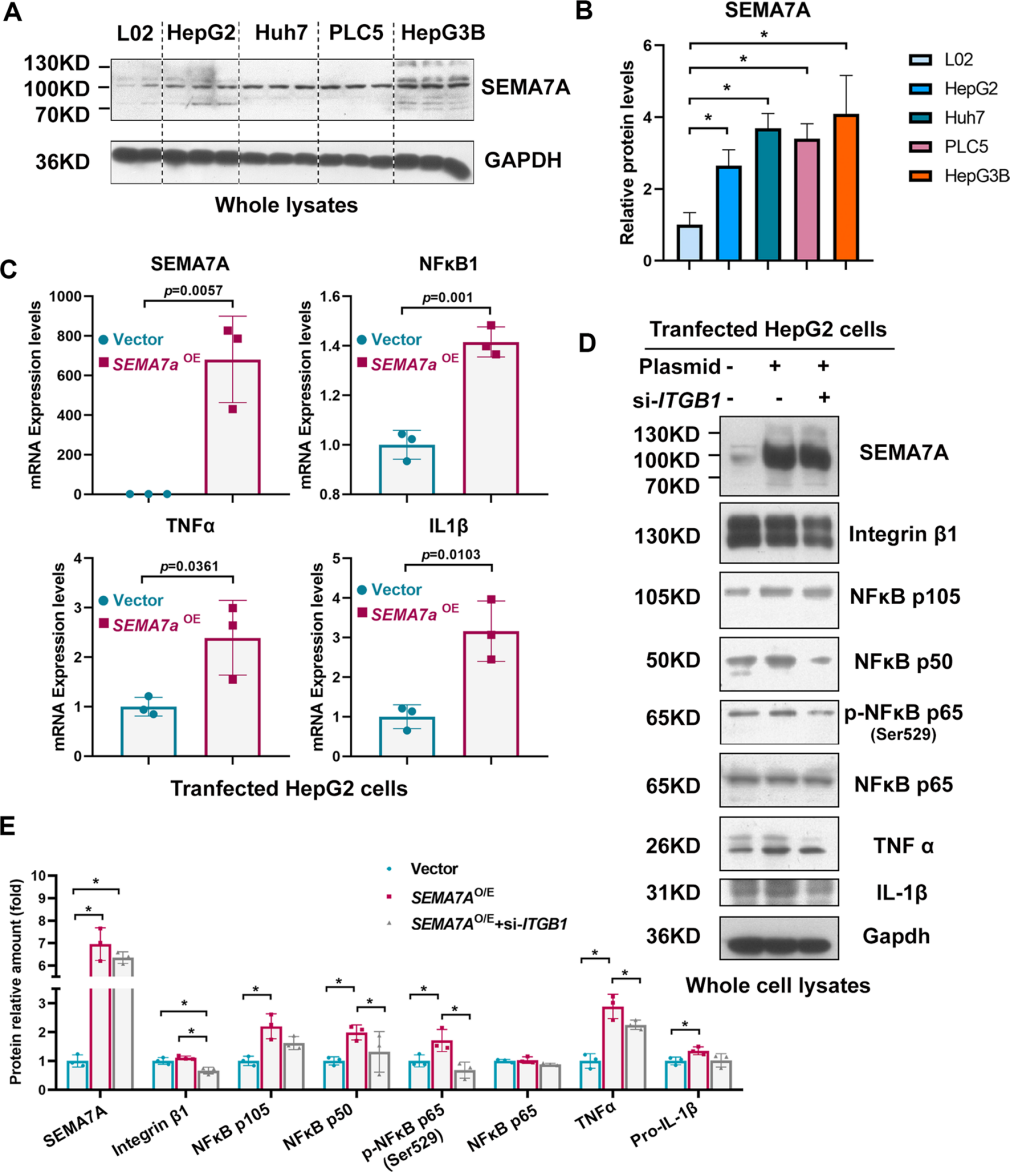
Ectopic expression of SEMA7A promotes NF-κB p105 procession and activates the NF-κB p50/p65 pathway. **a** and** b** Relative Sema7a expression in HCC cell lines (HepG2, Huh7, PLC5, and Hep3B) compared with an immortalized normal human hepatic cell line (L02). **c** Sema7a overexpression efficiency and relative NF-κB1, TNFα and IL1β mRNA levels in transfected HepG2 cell lines (n = 3). **d** and **e** Western blotting analysis of the relative levels of Sema7a, integrin β1, NF-κB p105, NF-κB p50, p- NF-κB p65/NF-κB p65, p-IκB/IκB, TNFα and IL1β protein expression in transfected HepG2 cell lines following integrin β1 silencing (n = 3). Phosphorylation levels were measured by the phosphor/total protein ratio. The qPCR data were analysed by the independent-samples Student’s t test and differences among the groups were determined by one-way ANOVA with Tukey’s post hoc tests or by Kruskal‒Wallis test with Dunn’s post hoc test analysis. *Means *p* < 0.05

Further experiments showed that SEMA7A^WT^ was elevated in four HCC cell lines, including HepG2, Huh7, PLC5, and HepG3B cells (Fig. 6A&B). To explore the potential biological function of SEMA7A^WT^, we selected HepG2 cells as an overexpression model according to the SEMA7A^WT^ expression patterns. Plasmids with the *SEMA7A*^WT^ gene were transfected into cells and the overexpression effectiveness was assessed by qPCR. The qPCR results suggested that the inflammatory response could be induced via SEMA7A^WT^ overexpression in HepG2 cells, as shown by increased *NFKB1*, *TNF-α* and *IL-1β* (Fig. 6C). As mentioned previously, integrin β1 is closely related to NF-κB p105 procession. The relative levels of NF-κB p105, NF-κB p50, p- NF-κB p65/NF-κB p65, p-IκB/IκB, TNFα and IL1β protein expression increased in transfected HepG2 cell lines and decreased following integrin β1 silencing (Fig. 6D&E). Subsequent wound healing and cell proliferation assays proved that upregulated SEMA7A^WT^ could accelerate the migratory (Additional file 3: Fig. S2A&B) and proliferation (Additional file 3: Fig. S2C) capability of HepG2 cells.

**Fig. 7:**
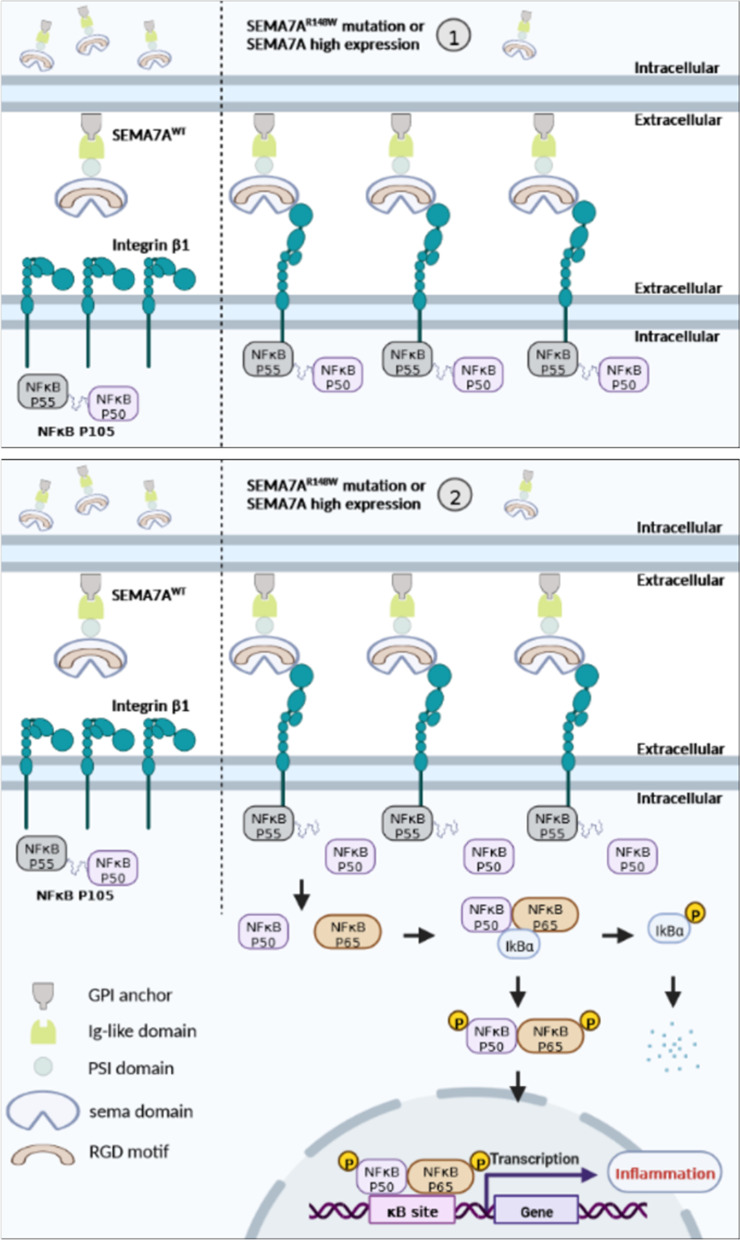
The potential mechanism diagram in hepatocytes. Integrin β1 binds to NF-κB p105 and activates downstream signalling in *SEMA7A*^R148W^ mutation and SEMA7A high expression models

Taken together, these results revealed that Sema7a^WT^ (SEMA7A^WT^) is upregulated in the HCC patients, mouse model and four cell lines. The elevated SEMA7A^WT^ activates the NF-κB p50/p65 pathway by integrin β1 and induces an inflammatory response. Therefore, SEMA7A^WT^ is of extensive clinical significance and might be a therapeutic target in inflammation and tumorigenesis (Fig. 7).

## Discussions

In this study, we reported a potential protein complex consisting of Sema7a (SEMA7A), integrin β1 and NF-κB p105. In this complex, integrin β1 likely acts as a bridge that links Sema7a (SEMA7A) and NF-κB p105 and mediates NF-κB p105 procession and downstream signalling activation, subsequently promoting hepatic inflammation. This pathological process is promoted by *Sema7a*^R145W^ (*SEMA7A*^R148W^) mutation. Meanwhile, Sema7a^WT^ (SEMA7A^WT^) expression was significantly increased in the HCC patients and mouse model, and might be an important target in clinical preventive treatment measures for alleviating inflammation in tumorigenesis and development.

In previous studies, *Sema7a*^R145W^ (*SEMA7A*^R148W^) reduced the expression of canalicular membrane bile acid transporters, resulting in intrahepatic cholestasis in mice [8]. Additionally, the cell membrane localization of *Sema7A*^R145W^ and its interaction with integrin β1 were significantly increased in *Sema7A*^R145W^ mutant mice. Sema7a^R145W^ (SEMA7A^R148W^) binds to integrin β1 to induce the phosphorylation of PKCα and activation of downstream signals, contributing to intrahepatic lipid accumulation and aggravating NAFLD [9]. Moreover, in this study, *Sema7A*^R145W^ bound to integrin β1 and activated NF-κB p50/p65 signalling, promoting inflammatory responses. Therefore, according to the definition of gene mutation [38] and the above experimental results, the *Sema7A*^R145W^ (*SEMA7A*^R148W^) mutation could be considered a gain-of-function mutation. However, researches on the regulatory mechanisms of the signalling pathway associated with the *Sema7A*^R145W^ (*SEMA7A*^R148W^) mutation is extremely limited. Therefore, it is necessary to determine the molecular mechanisms involved in the *Sema7A*^R145W^ (*SEMA7A*^R148W^) mutation.

The endogenous and exogenous co-IP data (Fig. 3B, D-G) revealed that integrin β1 could bind to the C-terminus of NF-κB p105. This interaction was elevated in *Sema7a*^R145W^ homozygous mouse livers. Unfortunately, we could not determine the exact binding site between integrin β1 and NF-κB p105. In addition, since we did not observe the binding of the 50 kDa protein in Fig. 3B, we hypothesized that integrin β1 cannot bind to NF-κB p50 and thus did not verify the interaction between integrin β1 and the N-terminus of NF-κB p105. However, this issue should be demonstrated clearly in our future studies. Meanwhile, because exogenous co-IP can only support the evidence of interactions between two proteins, whether directly or indirectly, we could only confirm that NF-κB p105 was included in the immunoprecipitated proteins of Sema7A^R145W^ (Fig. 3H) and that this interaction between Sema7A^R145W^ and NF-κB p105 was following integrin β1 inhibition. Thus, although we reported that there might exist a new protein complex, the present experimental results only support the information that integrin β1 can directly bind to the C-terminus of NF-κB p105 and probably plays a linking role between Sema7A^R145W^ and NF-κB p105.

Integrin β1 is a receptor of Sema7a (SEMA7A). Sema7a (SEMA7A) interacts with integrin β1, participates in various immunoinflammatory responses, and contributes to proinflammation cytokine release [39–41]. In this study, we found that elevated NF-κB p105 procession, advanced NF-κB p50/p65 signalling activation, proinflammatory cytokine production and inflammatory infiltration were displayed in homozygous *Sema7a*^R145W^ mutant mice. Inhibition of integrin β1 restrained NF-κB p50 generation, subsequently decreasing phosphorylated NF-κB p50/p65 levels and suppressing TNFα and IL1β production (Fig. 3I&J). According to the above results, we propose the hypothesis that integrin β1 acts as a bridge connecting Sema7a^R145W^ and NF-κB p105 and participates in proinflammatory responses in *Sema7a*^R145W^ mutant mice. Enhanced NF-κB p50 levels largely indicate NF-κB p105 procession. However, we noticed that there was no significant difference in the precursor protein p105 in WT and homozygous *Sema7a*^R145W^ mutant mice (Fig. 2A&B). Thus, we further evaluated the mRNA level of *NFKB1* and found that *NFKB1* significantly increased. The increased mRNA level reminded us that there might exist a quick dynamic balance of p105 protein translation and processing (Fig. 2F&G). However, this hypothesis still needs experimental supports, and we would like to focus on this topic in our future studies.

Integrin β1 is a transmembrane protein that mainly mediates cell-to-cell communication in various immunoinflammatory responses. Depending on different microenvironments, different cytokines or chemokines are secreted by different cells. According to these diverse signals, integrin β1 plays a dual proinflammatory and anti-inflammatory role. Thus, the immunoinflammatory consequences of integrin β1 could differ. For example, integrin β1-enriched extracellular vesicles from hepatocytes could mediate monocyte adhesion and contribute to liver inflammation in non-alcoholic steatohepatitis [42]. However, pancreas-specific ablation of integrin β1 is related to widespread inflammation and collagen deposition [43]. In addition, the effects of losing integrin β1 in the same mouse model could also be opposite at different ages [44]. Thus, depending on different microenvironments in different organs and cell types, the proinflammatory and anti-inflammatory functions of integrin β1 could be different but not conflicting. In our study, integrin β1 contributed to NF-κB p50/p65 activation and TNF-α and IL-1β secretion, which play key roles in proinflammation. However, other molecular mechanisms of integrin β1 in the *Sema7a*^R145W^ (*SEMA7A*^R148W^) mutation and high Sema7a^WT^ (SEMA7A^R148W^) expression in immunoinflammatory responses still need to be characterized in future studies.

It is well known that HCC, the foremost form of primary liver cancer, is frequently linked with continuous liver inflammation [17, 45, 46]. In a retrospective cohort study of 417 cancer-free patients with cirrhosis, 27% developed liver cancer after approximately 12 years [47]. To determine whether there was relevance between the *SEMA7A*^R148W^ mutation and HCC, we analysed 1804 HCC patients from the ICGC database. The *SEMA7A* gene mutation frequency varies widely in different countries; the cohort from China displayed the highest mutation frequency (5.4%) in HCC patients. However, the *SEMA7A*^R148W^ mutation was not detected in hepatocellular carcinoma but in endometrioid carcinoma (n = 1), adenocarcinoma (n = 2), and oesophageal adenocarcinoma (n = 1). Although the clinical significance of *SEMA7A*^R148W^ in HCC is limited, its generality of physiological function in gain-of-function mutation and high expression is worth exploring. We noticed that Sema7a^WT^ (SEMA7A^WT^) was significantly increased in HCC patients and a mouse model (Fig. 5A-C and F–H). Additionally, the interactions between integrin β1 and NF-κB p105 were elevated in the liver cancer group (Fig. 5I). Interestingly, the relevance of Sema7a^WT^ (SEMA7A^WT^) and HCC has not been previously reported. Further mechanistic studies demonstrated that similar to the *Sema7a*^R145W^ mutation, NF-κB p50 generation, NF-κB p50/p65 signalling activation, and proinflammation cytokine production were also observed in the HCC group (Fig. 5F-H). In addition, these effects were inhibited by integrin β1 silencing (Fig. 6D-E). Overall, our data indicated that the *Sema7A*^R145W^ (*SEMA7A*^R148W^) gain-of-function mutation and high Sema7A^WT^ (SEMA7A^WT^) expression had similar effects on inflammation promotion.

Since inflammation is a potential risk factor for tumour occurrence and development, SEMA7A^WT^ is probably an important factor in tumour progression. In addition, we noticed that cell migration and proliferation were stimulated in transfected HepG2 cells. However, some studies have reported that an adequate immune response might be a protective factor in certain cancers, contrasting evidence for protumorigenic functions for inflammation [34]. The molecular mechanisms of SEMA7A^WT^ and its receptors in tumour formation and development need to be determined in our next study.

Based on research on the *Sema7A*^R145W^ (*SEMA7A*^R148W^) mutation [9], the membrane localization of SEMA7A and integrin β1 was thought to increase without expression level changes. Here, we show a new protein‒protein interaction among Sema7a, integrin β1 and NF-κB p105. This interaction strongly increased after *Sema7a*^R145W^ mutation and promoted the inflammatory response. According to our assumption, this phenomenon might be explained by the upregulated membrane localization of Sema7A^R145W^ (SEMA7A^R148W^) and integrin β1 benefiting the exposure of their protein structure, especially the binding site for NF-κB p105. Additionally, this protein‒protein interaction and proinflammatory effect still needs to be described in liver nonparenchymal cells and immune cells. These questions need to be answered in our further studies.

In conclusion, we emphasized the important role of Sema7a (SEMA7A) and its receptor integrin β1 in NF-κB p105 procession and NF-κB p50/p65 pathway activation, which had never been reported. Our data also supported that the *Sema7A*^R145W^ (*SEMA7A*^R148W^) mutation and high Sema7a^WT^ (SEMA7A^WT^) expression both result in NF-κB p50 generation, NF-κB p50/p65 signalling activation and inflammatory cytokine production in an integrin β1-dependent manner.

## Supplementary Information

**Additional file 1** Supplementary Table 1 and Table 2.

**Additional file 2** Supplymentary Figure 1.

**Additional file 3.** Supplymentary Figure 2.

**Additional file 4.** Raw Data of Western Blotting.

## Data Availability

The datasets supporting the conclusions of this article are available in the ICGC Data Portal [https://dcc.icgc.org/] and TCGA database [http://cancergenome.nih.gov/].

## Abbreviations

SEMA7A/Sema7a: Semaphorin 7A
GPI: Glycosylphosphatidylinositol
PFIC: Progressive familial intrahepatic cholestasis
NAFLD: Nonalcoholic fatty liver disease
TNF-α: Tumor necrosis factor α
IL-1β: Interleukin-1β
NF-κB: Nuclear factor kappa-B
IκBα: Inhibitor of κB alpha
HCC: Hepatocellular carcinoma
WT: Wild-type
HO: Homozygous
DEN: Diethylnitrosamine
i.p: Intraperitoneal
HE: Hematoxylin–eosin
Co-IP: Co-immunoprecipitations
PPI: Protein–protein interaction
ICGC: International cancer genome consortium
TCGA: The cancer genome atlas program
