# Supplementary material for: Semaphorin 7A interacts with nuclear factor NF-kappa-B p105 via integrin β1 and mediates inflammation
Source: Cell Commun Signal. 2023 Jan 30;21:24. doi: 10.1186/s12964-022-01024-w (PMC9885601; doi:10.1186/s12964-022-01024-w)
Supplement: Supplementary file 2 — Additional file 1 Supplementary Table 1 and Table 2. [file 12964_2022_1024_MOESM2_ESM.docx]

**Supplementary Table 1. List of *SEMA7A* gene mutation identified by ICGC liver hepatocellular carcinoma database.**

| Patient ^b^ | Protein  Change | cDNA  position ^a^ | Exon  (14 in total) | Mutation Type | Sex | Diagnosis  Age | Sample  ID | Race |
| --- | --- | --- | --- | --- | --- | --- | --- | --- |
| 1 | ***C126**** | c.378C>A | 4 | Stop gained | Male | 57 | DO229037 | Asian |
| 2 | ***L132I*** | c.394C>A | 4 | Missense | Female | 76 | DO23136 | Asian |
| *3* | ***R135G*** | c.403A>G | 4 | Missense | Male | 54 | DO22956 | Black |
| *4* | ***R202W*** | c.604C>T | 6 | Missense | Male | 76 | DO22908 | White |
| 5 | ***F203L*** | c.609C>A | 6 | Missense | Female | 41 | DO229029 | Asian |
| 6 | ***N221K*** | c.663C>A | 7 | Missense | Male | 77 | DO228974 | Asian |
| *7* | ***D237N*** | c.709G>A | 7 | Missense | Female | 78 | DO44879 | White |
| 8 | ***D238E*** | c.714C>A | 7 | Missense | Male | 68 | DO229031 | Asian |
| 9 | ***S277**** | c.830C>A | 8 | Stop gained | Male | 49 | DO229025 | Asian |
| 10 | ***N282K*** | c.846C>A | 8 | Missense | Male | 76 | DO229038 | Asian |
| 11 | ***L309I*** | c.925C>A | 8 | Missense | Male | 57 | DO229037 | Asian |
| 12 | ***D311E*** | c.933C>A | 8 | Missense | Male | 62 | DO229015 | Asian |
| 13 | ***S313R*** | c.939C>A | 8 | Missense | Male | 65 | DO229006 | Asian |
| 14 | ***D318E*** | c.954C>A | 8 | Missense | Male | 62 | DO228971 | Asian |
| 15 | ***H355N*** | c.1063C>A | 9 | Missense | Male | 26 | DO229019 | Asian |
| 16 | ***S357R*** | c.1071C>A | 9 | Missense | Male | 26 | DO229019 | Asian |
| 17 | ***P363H*** | c.1088C>A | 9 | Missense | Female | 73 | DO229024 | Asian |
| 18 | ***Q379L*** | c.1136A>T | 10 | Missense | Male | 24 | DO52318 | White |
| 19 | ***T431A*** | c.1291A>G | 10 | Missense | Male | 64 | DO49722 | White |
| 20 | ***D432E*** | c.1296C>A | 11 | Missense | Male | 67 | DO228985 | Asian |
| 21 | ***D432E*** | c.1296C>A | 11 | Missense | Male | 76 | DO229038 | Asian |
| 22 | ***R433K*** | c.1298G>A | 11 | Missense | Female | 72 | DO50970 | White |
| 23 | ***P442Q*** | c.1325C>A | 11 | Missense | Male | 56 | DO229008 | Asian |
| 24 | ***A450T*** | c.1348G>A | 11 | Missense | Female | 68 | DO52325 | White |
| 25 | ***Q466H*** | c.1398G>T | 11 | Missense | Male | 77 | DO228974 | Asian |
| 26 | ***M468V*** | c.1402A>G | 11 | Missense | Male | 81 | DO45255 | Asian |
| 27 | ***S469**** | c.1406C>A | 11 | Stop gained | Female | 64 | DO229018 | Asian |
| 28 | ***L564M*** | c.1690C>A | 14 | Missense | Male | 57 | DO229037 | Asian |
| 29 | ***S570F*** | c.1709C>T | 14 | Missense | Male | 60 | DO52267 | Asian |
| 30 | ***R578H*** | c.1733G>A | 14 | Missense | Male | 70 | DO229054 | Asian |
| 31 | ***R622S*** | c.1864C>A | 14 | Missense | Male | 64 | DO228948 | Asian |
| 32 | ***H639N*** | c.1915C>A | 14 | Missense | Male | 57 | DO229037 | Asian |
| 33 | ***A649T*** | c.1945G>A | 14 | Missense | Male | 72 | DO23210 | Asian |

^a^ Mutation data of patients #3/4/7/18/19/24/29 were obtained from the LIHC-US, #1/5/6/8-17/20/21/23/25/27/28/30-32was obtained from the LICA-CN, #26 was obtained from the LIRI-JP, #2 and #33 were obtained from the LINC-JP, #22 were obtained from the LICA-FR.

^b^ Nucleotide position is according to the NM_003612 sequence, starting from the first nucleotide of the translation start codon.

**Supplementary Table 2: Primer sequences**

| Gene name | Sequence（5'-3'） |
| --- | --- |
| *SEMA7A-human* | *F: TCATCAAAGCCACCATCG* |
|  | *R: AGCTCACATACAGCTTCCTCC* |
| *NFKB1-human* | *F: AACAGAGAGGATTTCGTTTCCG* |
|  | *R: TTTGACCTGAGGGTAAGACTTCT* |
| *TNFA-human* | *F: ATCCTGGGGGACCCAATGTA* |
|  | *R: AAAAGAAGGCACAGAGGCCA* |
| *IL1B-human* | *F: TCGCCAGTGAAATGATGGCT* |
|  | *R: TGGAAGGAGCACTTCATCTGTT* |
| *GAPDH-human* | *F:CTGACTTCAACAGCGACACC* |
|  | *R: TGCTGTAGCCAAATTCGTTGT* |
| *Sema7a-mouse* | *F: CCAGAAGGAACAGTGGTTTGGC* |
|  | *R: ACTGTCCTCTGGGCTTGGTGTT* |
| *Nfkb1-mouse* | *F: GGTATGCACCGTAACAGCAG* |
|  | *R: GCTTCCCTCTGTCATCCGT* |
| *Tnfa-mouse* | *F: CCCTCACACTCAGATCATCTTCT* |
|  | *R: GCTACGACGTGGGCTACAG* |
| *Il1b-mouse* | *F:GTGAAATGCCACCTTTTGACAGTG* |
|  | *R: CCTGCCTGAAGCTCTTGTTG* |
| *Gapdh-mouse* | *F: TGACCTCAACTACATGGTCTACA* |
|  | *R: CTTCCCATTCTCGGCCTTG* |
