# Supplementary figures and images for: Semaphorin 7A interacts with nuclear factor NF-kappa-B p105 via integrin β1 and mediates inflammation
Source: Cell Commun Signal. 2023 Jan 30;21:24. doi: 10.1186/s12964-022-01024-w (PMC9885601; doi:10.1186/s12964-022-01024-w)

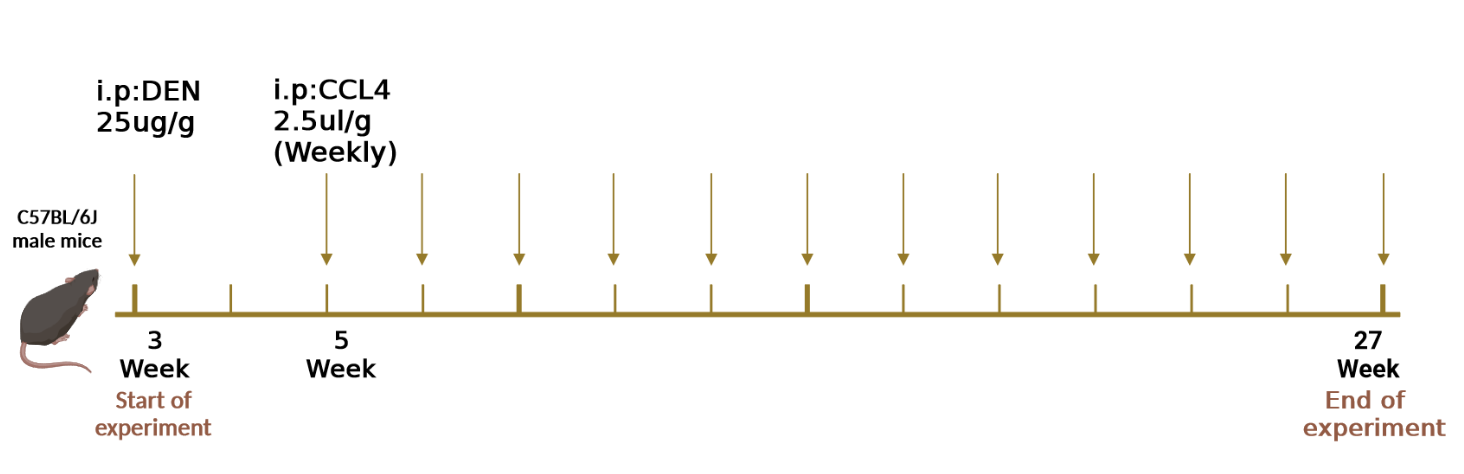

Supplement: Supplementary file 3 — Additional file 2 Supplymentary Figure 1. [file 12964_2022_1024_MOESM3_ESM.png]

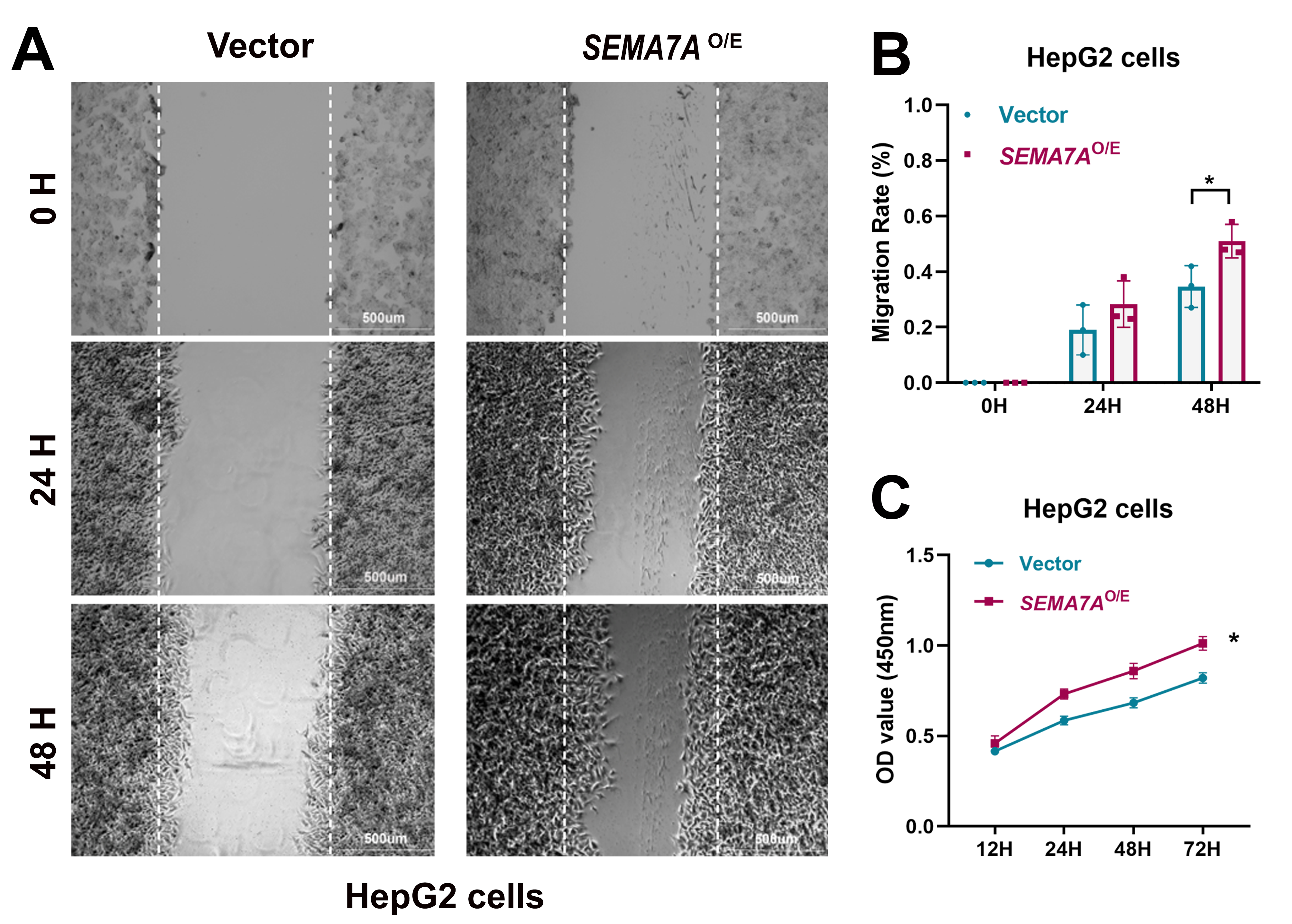

Supplement: Supplementary file 4 — Additional file 3. Supplymentary Figure 2. [file 12964_2022_1024_MOESM4_ESM.png]
