# Supplementary material for: Semaphorin 7A interacts with nuclear factor NF-kappa-B p105 via integrin β1 and mediates inflammation
Source: Cell Commun Signal. 2023 Jan 30;21:24. doi: 10.1186/s12964-022-01024-w (PMC9885601; doi:10.1186/s12964-022-01024-w)
Supplement: Supplementary file 5 — Additional file 4. Raw Data of Western Blotting. [file 12964_2022_1024_MOESM5_ESM.pptx]

## Slide 1
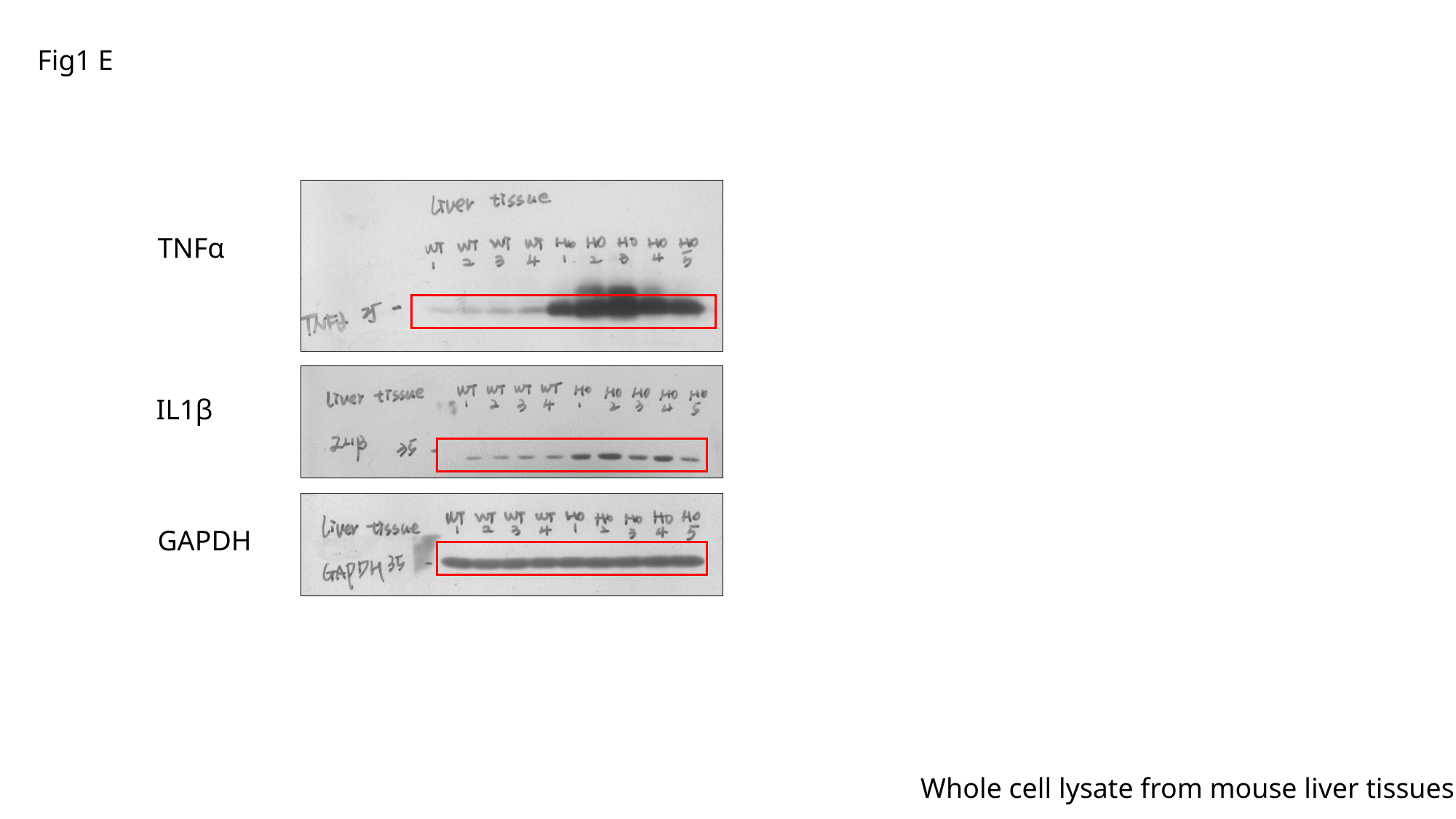

Fig1 E
TNFα
IL1β
GAPDH
Whole cell lysate from mouse liver tissues

## Slide 2
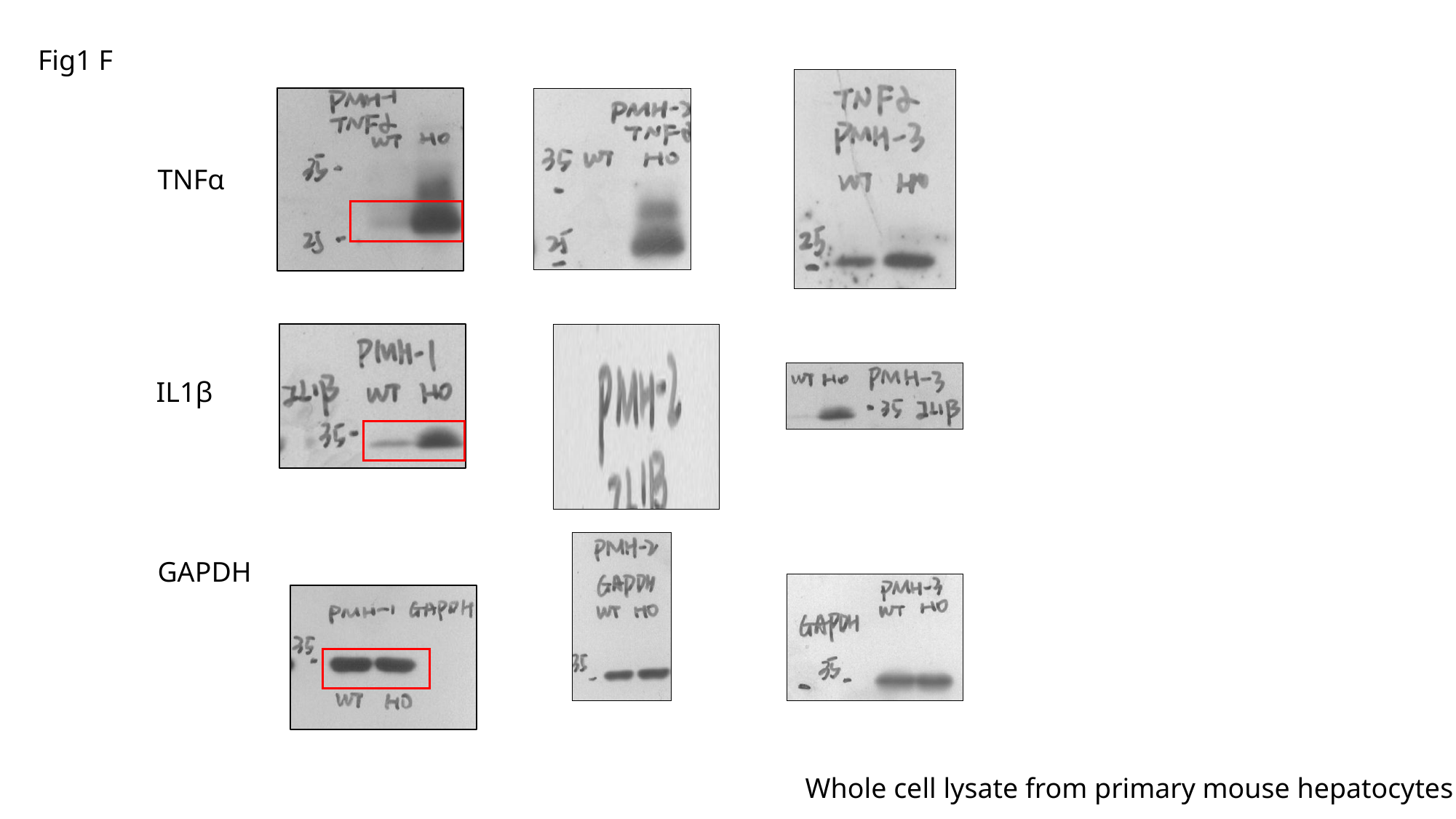

Fig1 F
TNFα
IL1β
GAPDH
Whole cell lysate from primary mouse hepatocytes

## Slide 3
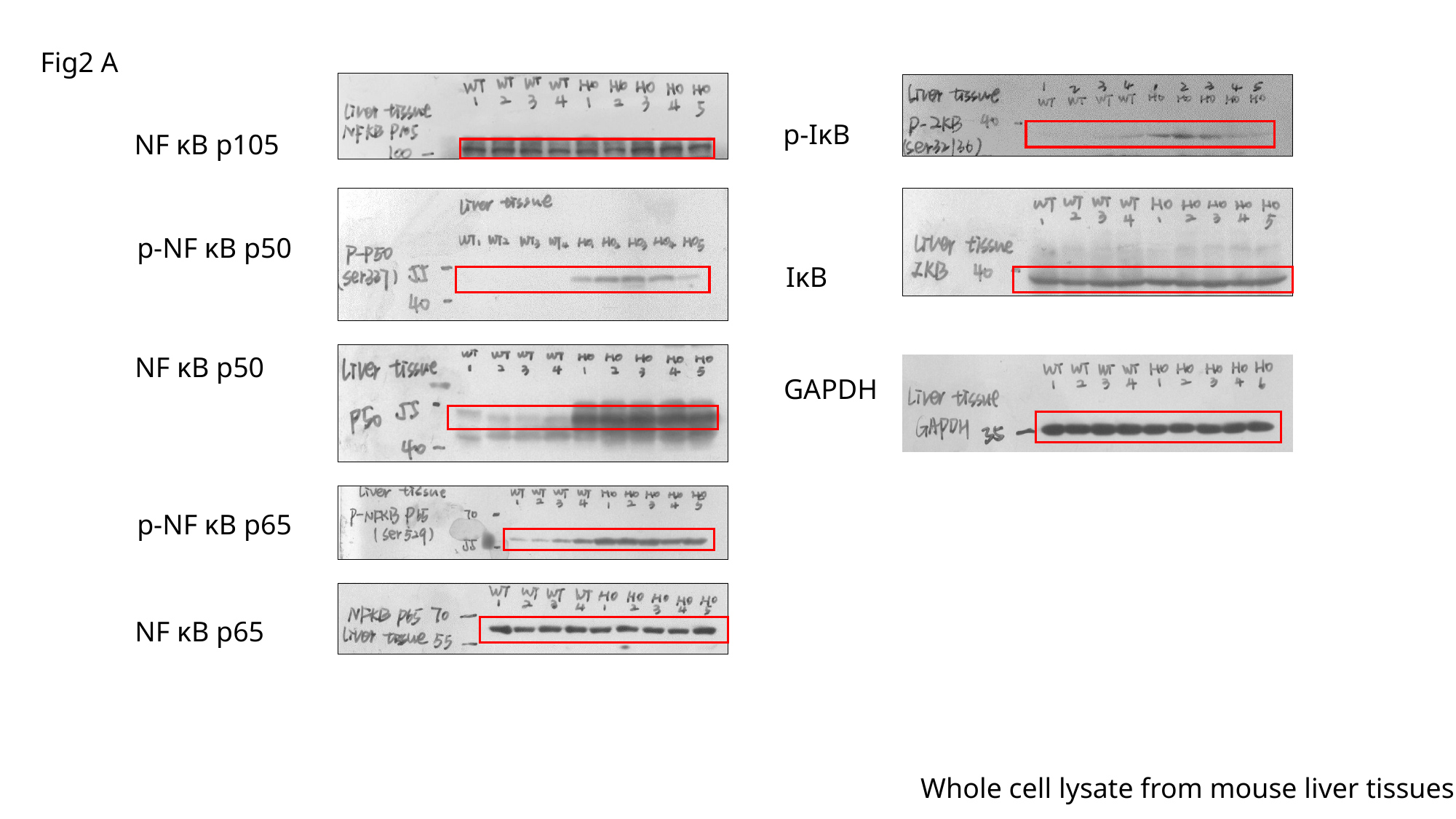

Fig2 A
p-IκB
NF κB p105
p-NF κB p50
IκB
NF κB p50
GAPDH
p-NF κB p65
NF κB p65
Whole cell lysate from mouse liver tissues

## Slide 4
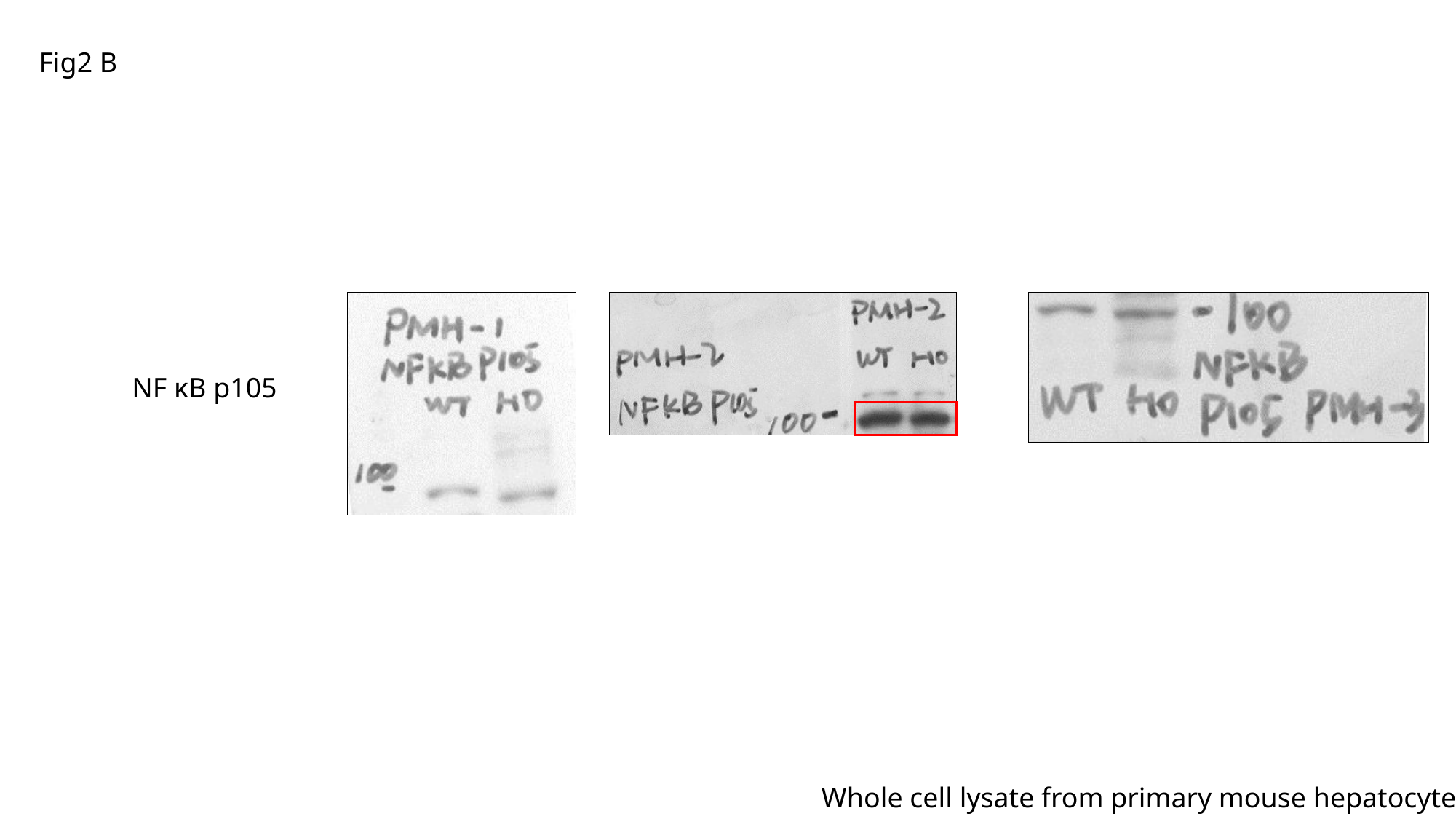

Fig2 B
NF κB p105
Whole cell lysate from primary mouse hepatocytes

## Slide 5
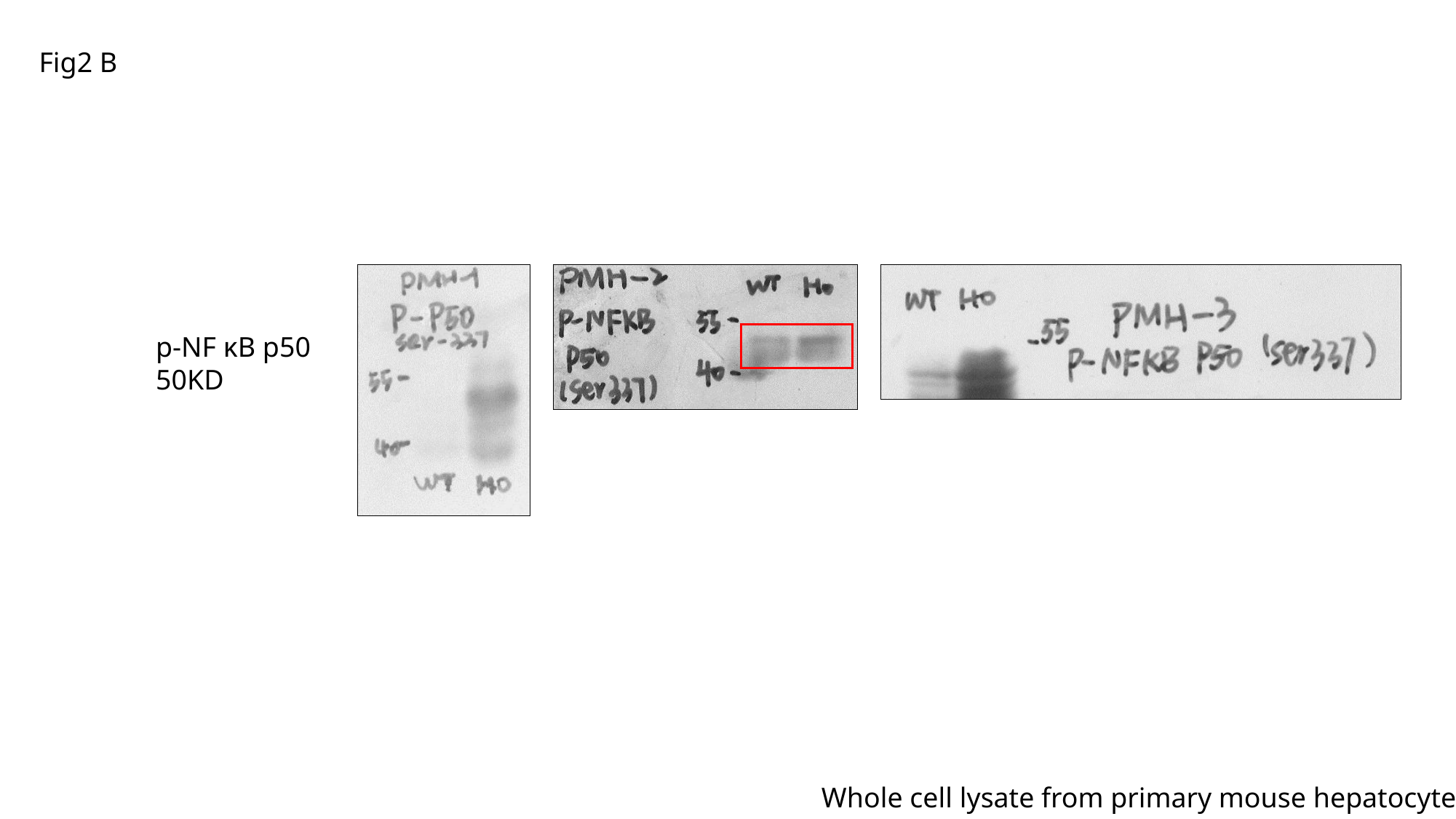

Fig2 B
p-NF κB p50
50KD
Whole cell lysate from primary mouse hepatocytes

## Slide 6
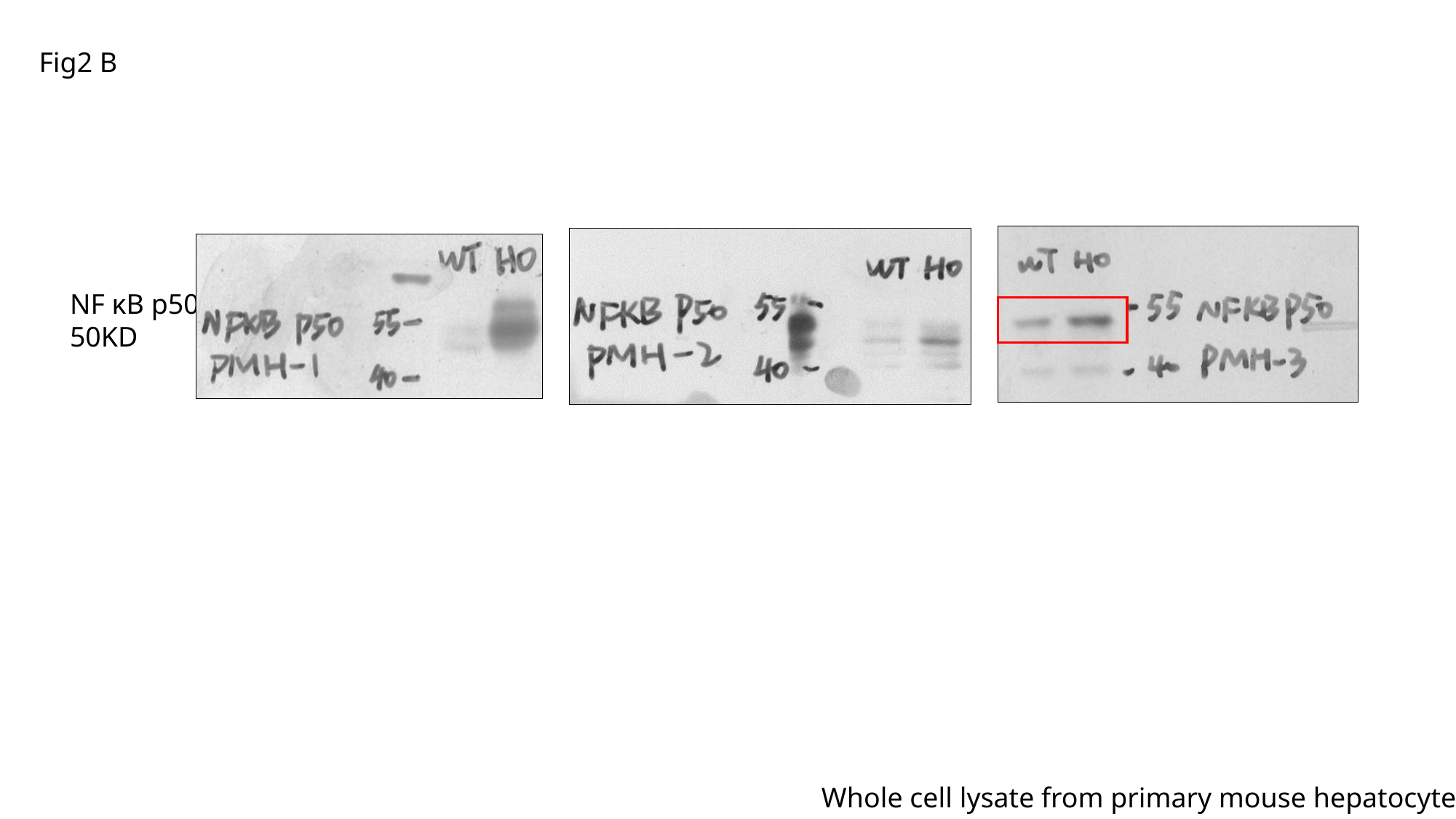

Fig2 B
NF κB p50
50KD
Whole cell lysate from primary mouse hepatocytes

## Slide 7
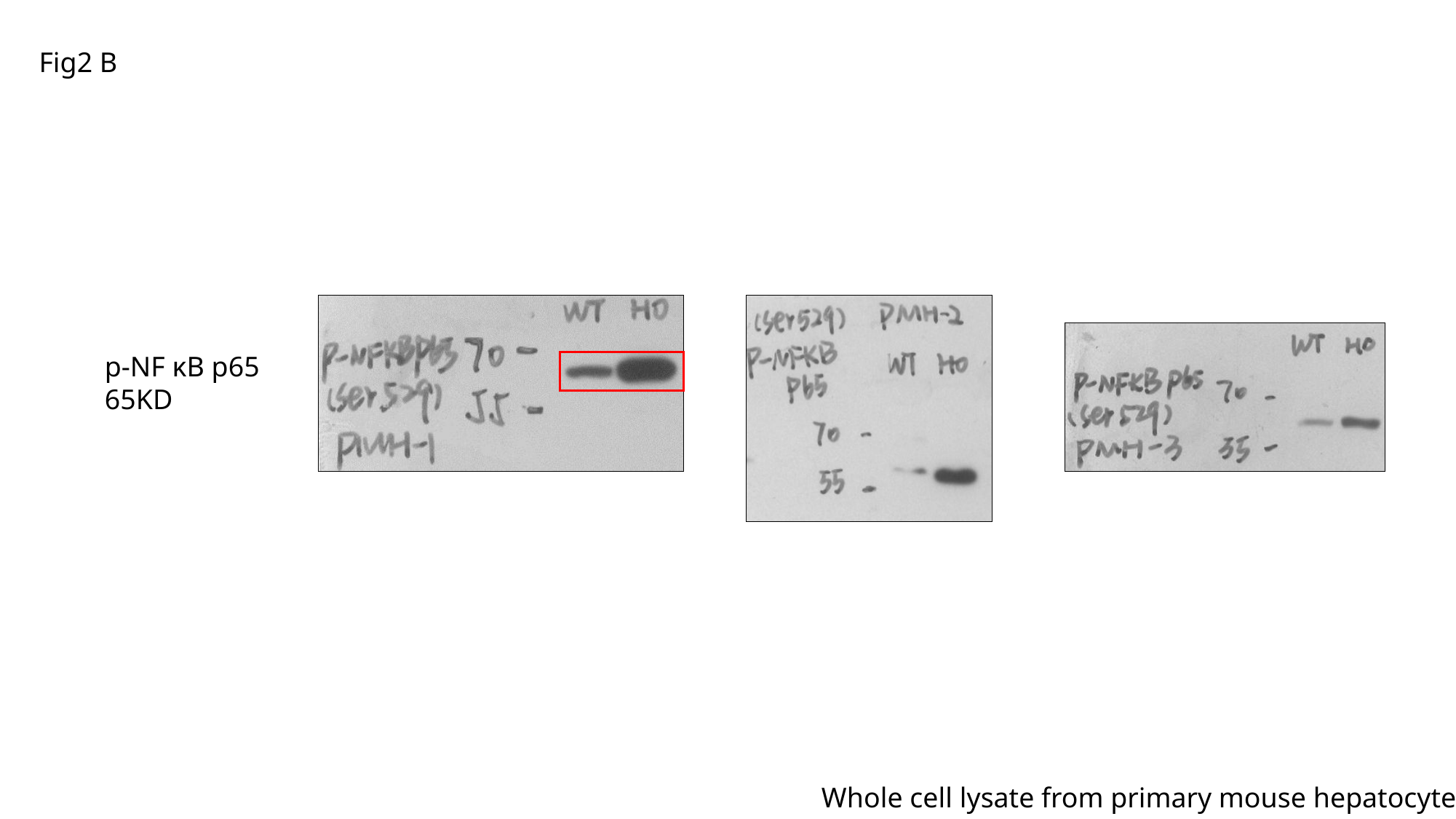

Fig2 B
p-NF κB p65
65KD
Whole cell lysate from primary mouse hepatocytes

## Slide 8
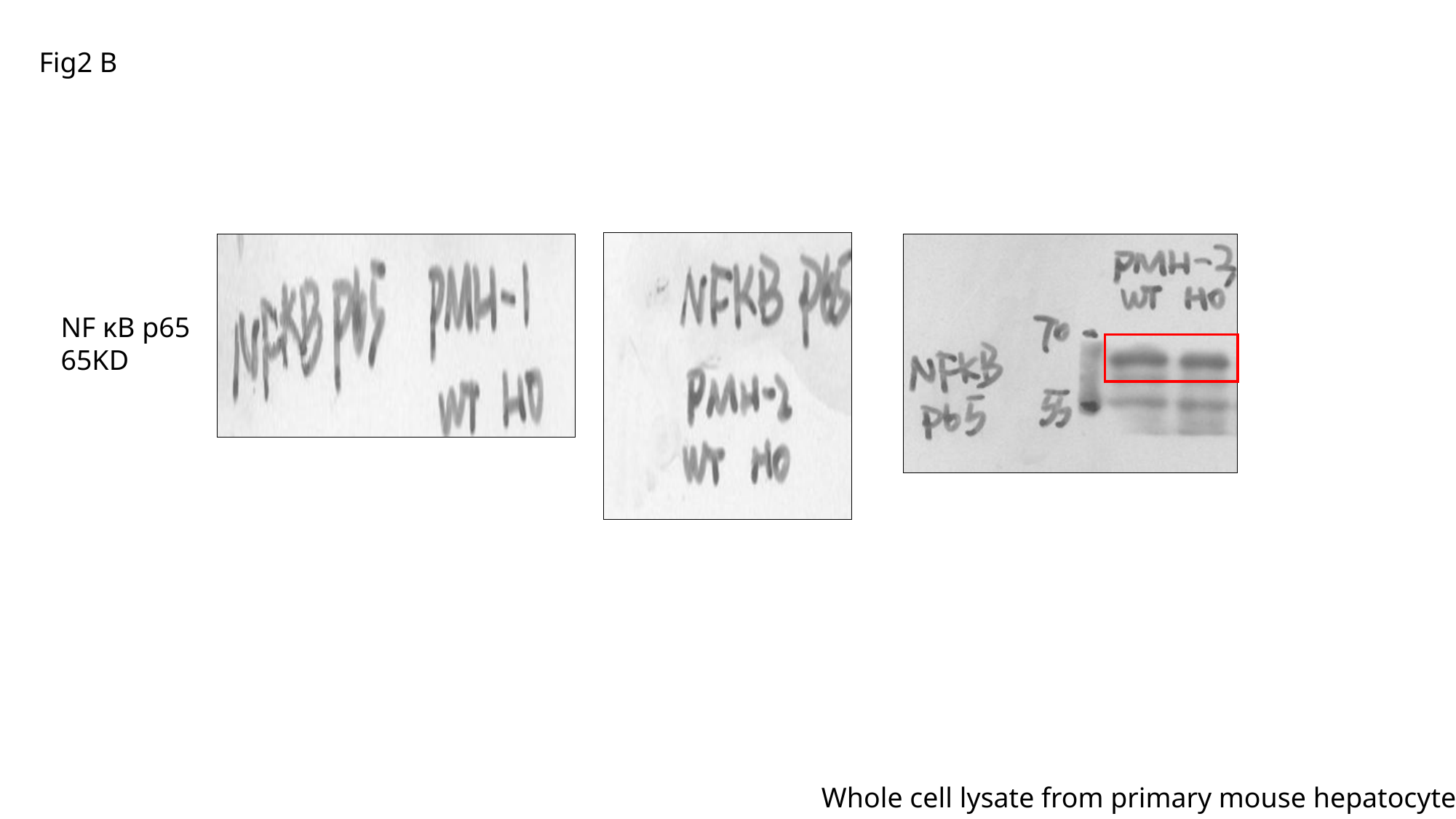

Fig2 B
NF κB p65
65KD
Whole cell lysate from primary mouse hepatocytes

## Slide 9
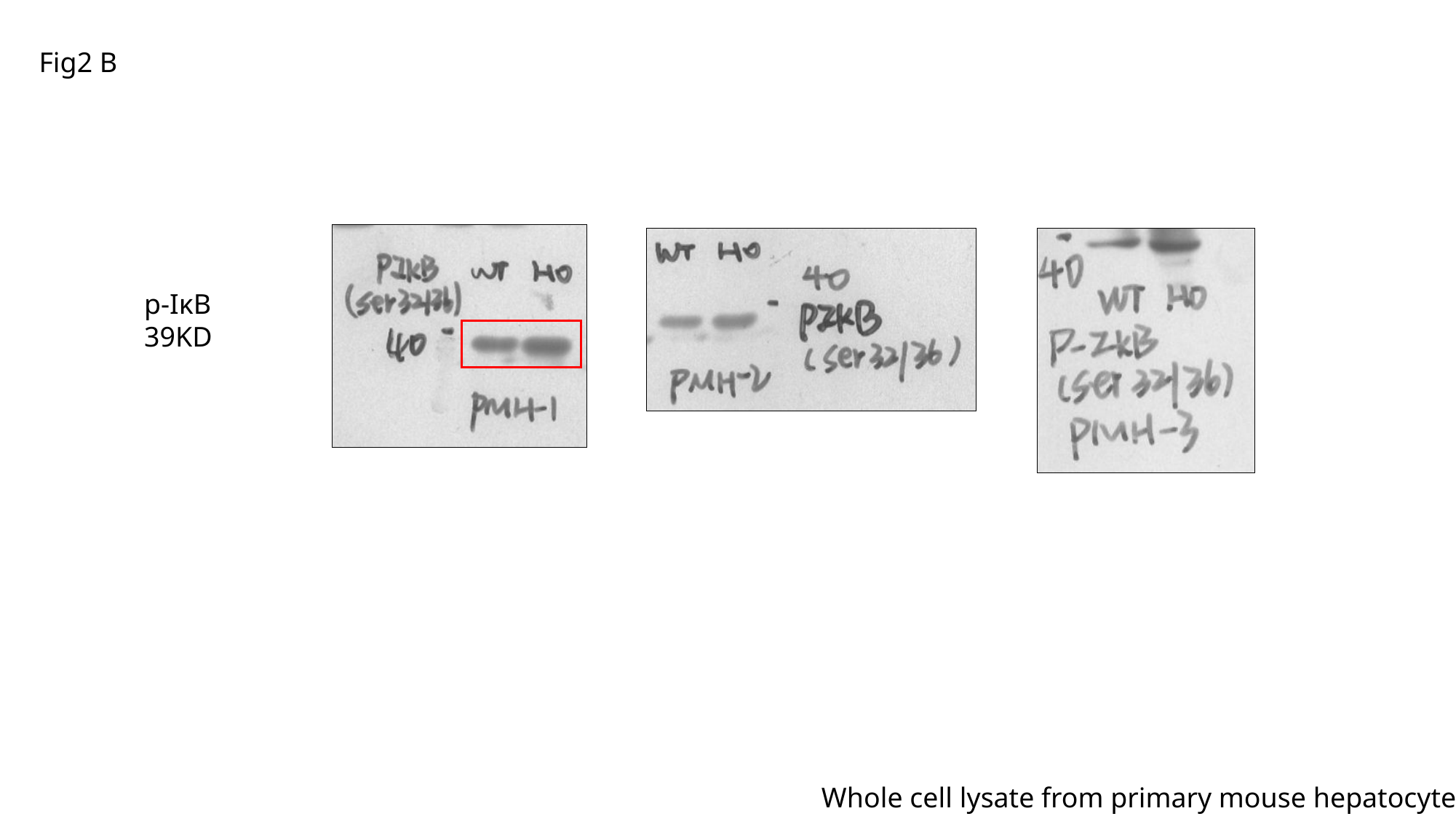

Fig2 B
p-IκB
39KD
Whole cell lysate from primary mouse hepatocytes

## Slide 10
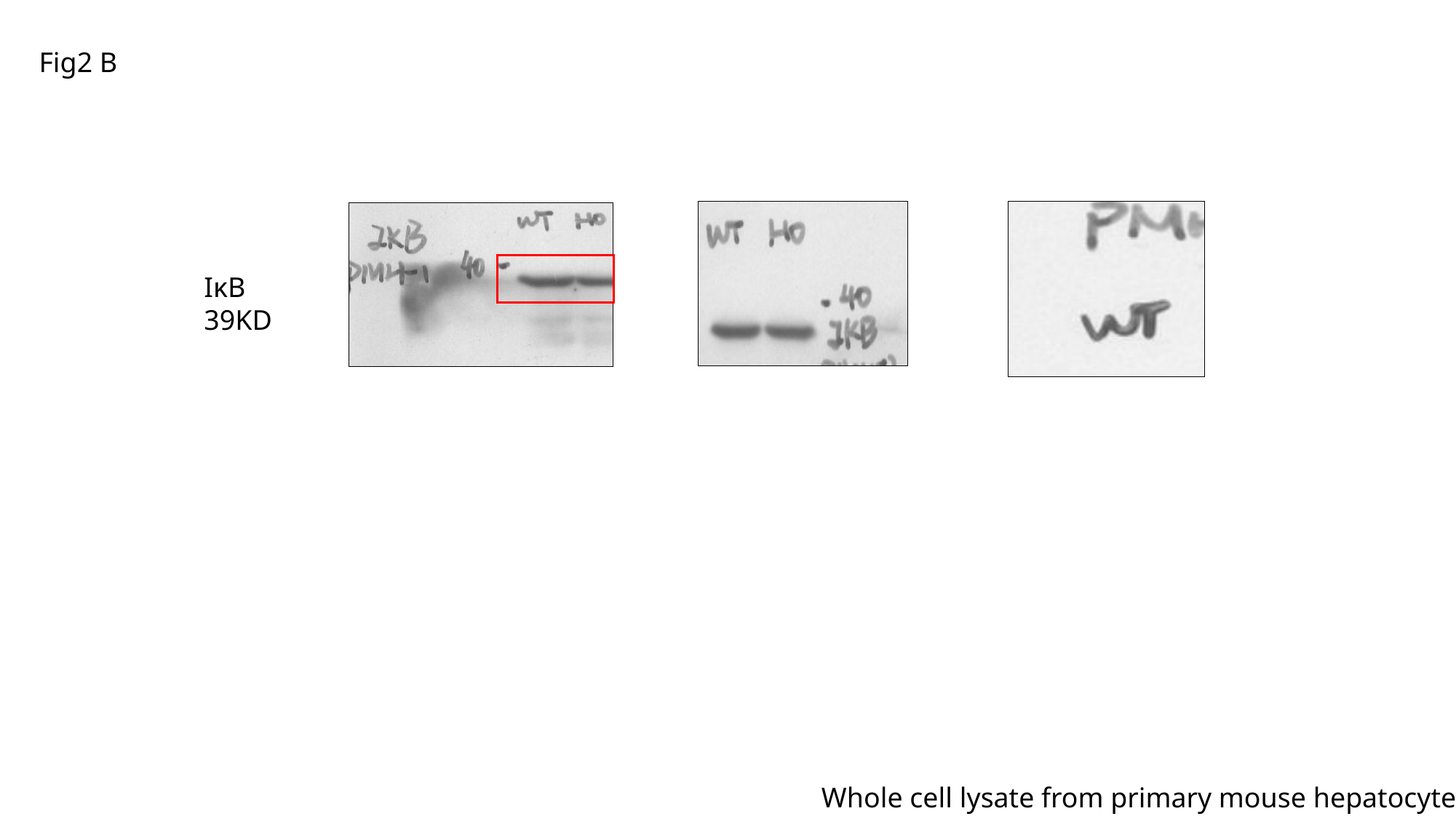

Fig2 B
IκB
39KD
Whole cell lysate from primary mouse hepatocytes

## Slide 11
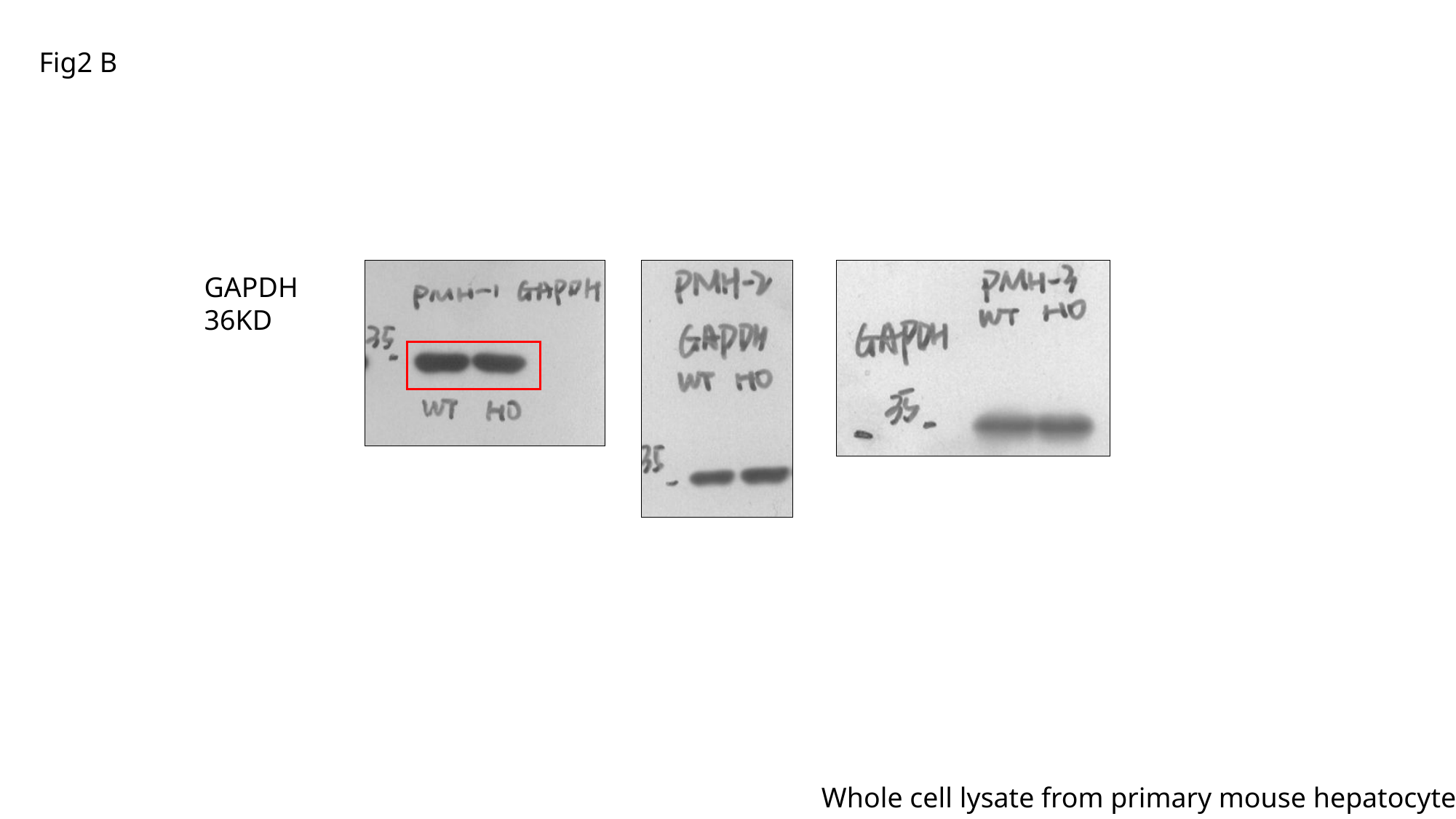

Fig2 B
GAPDH
36KD
Whole cell lysate from primary mouse hepatocytes

## Slide 12
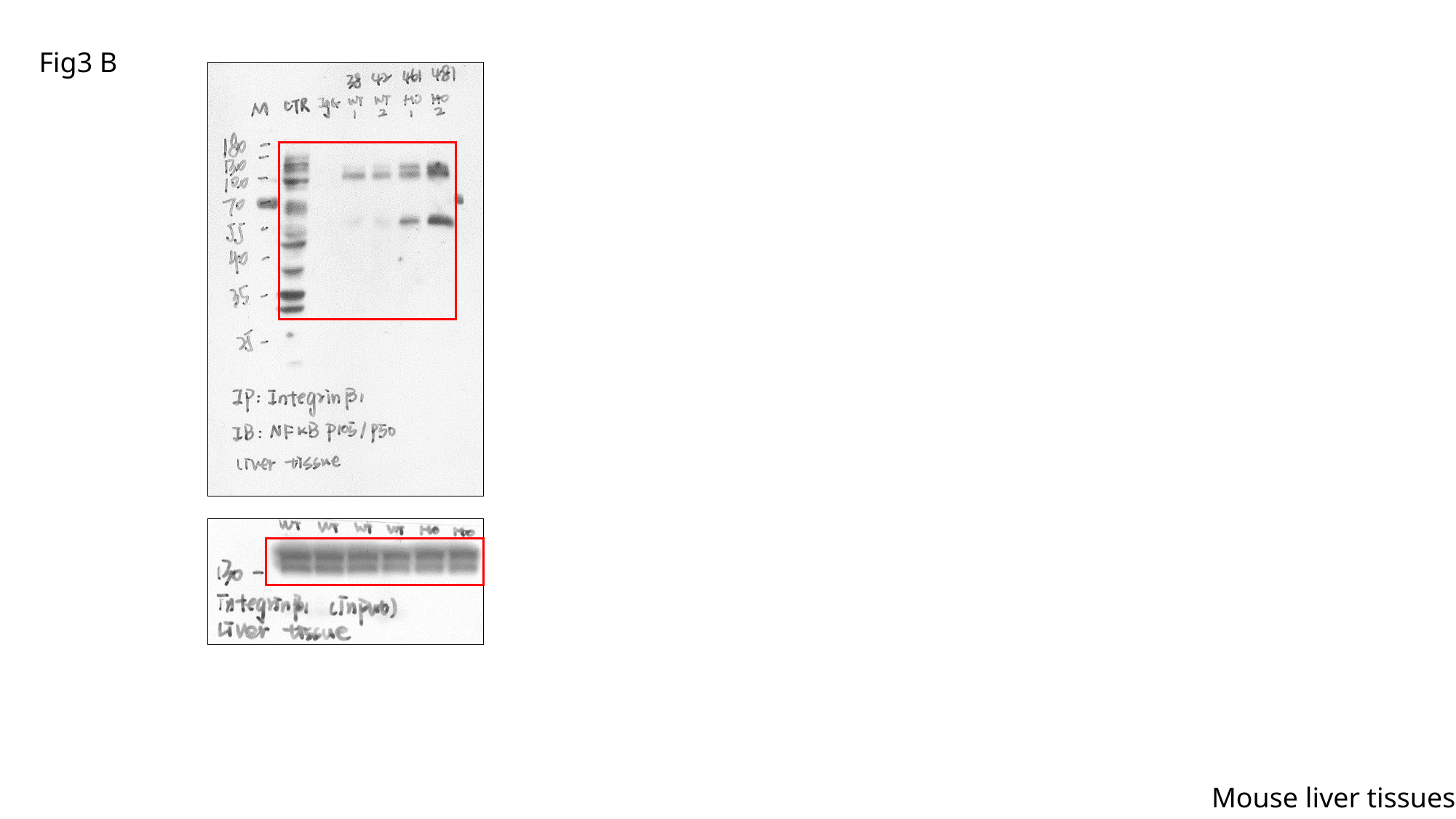

Fig3 B
Mouse liver tissues

## Slide 13
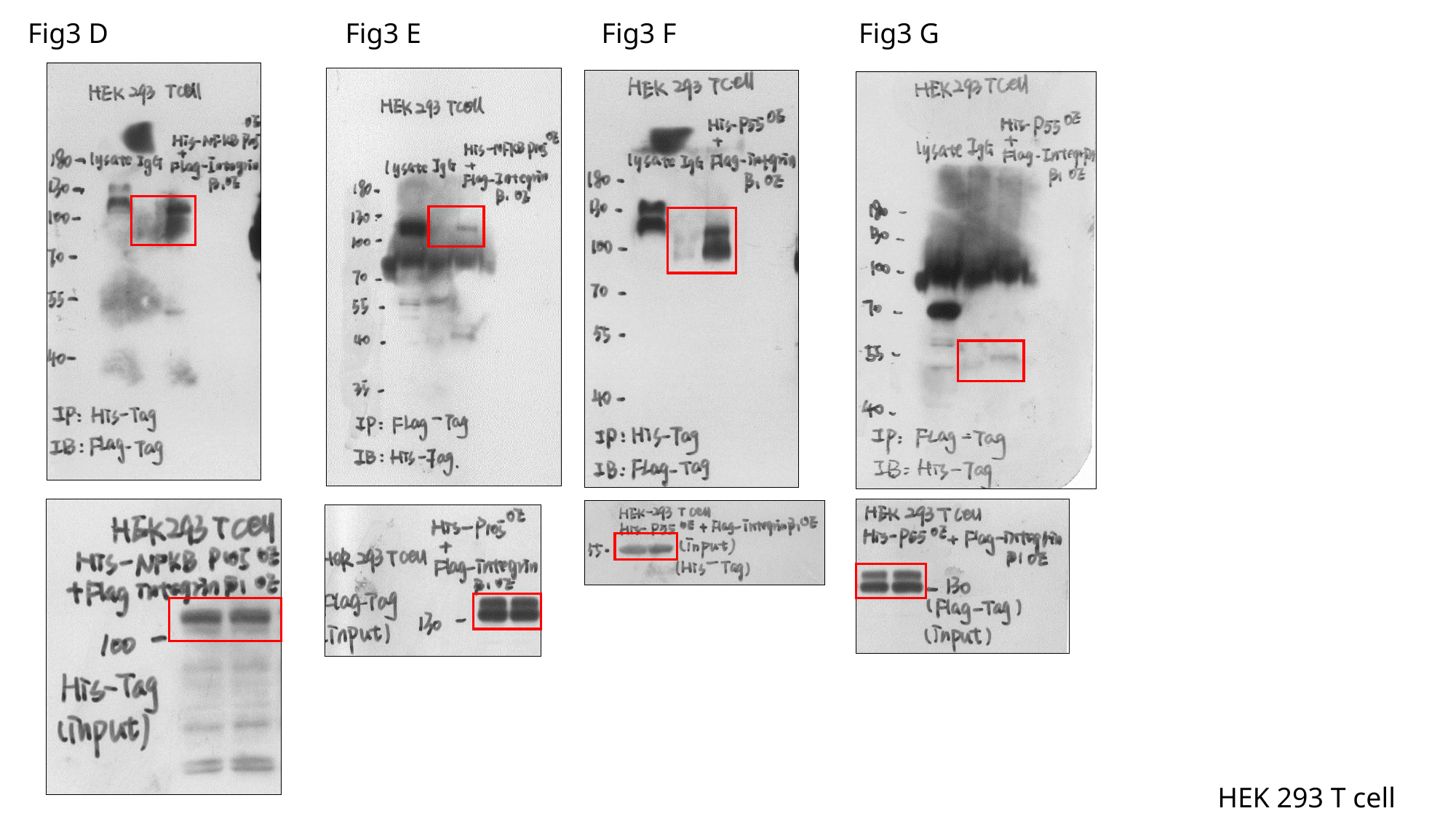

Fig3 F
Fig3 G
Fig3 D
Fig3 E
HEK 293 T cell

## Slide 14
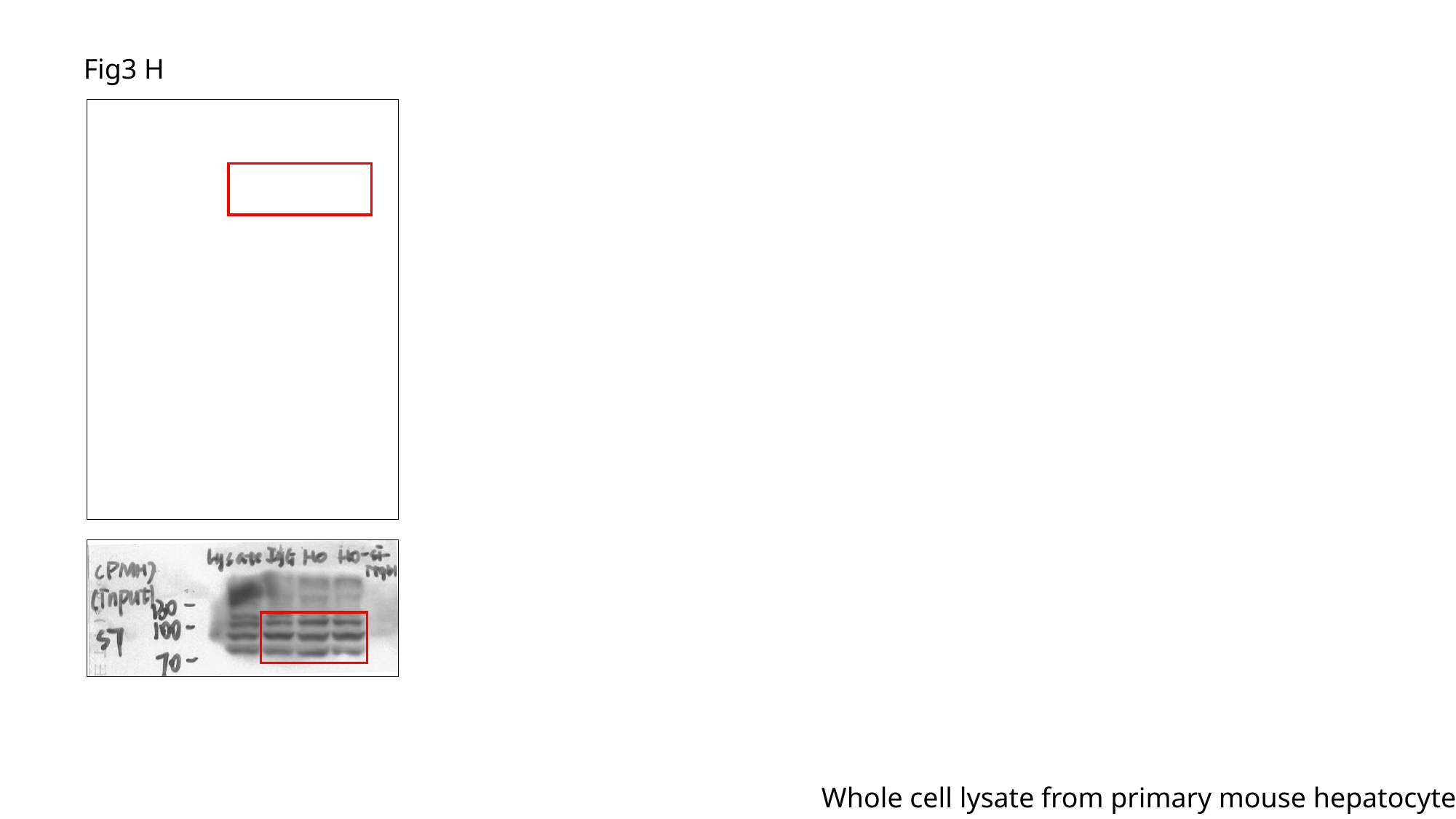

Fig3 H
Whole cell lysate from primary mouse hepatocytes

## Slide 15
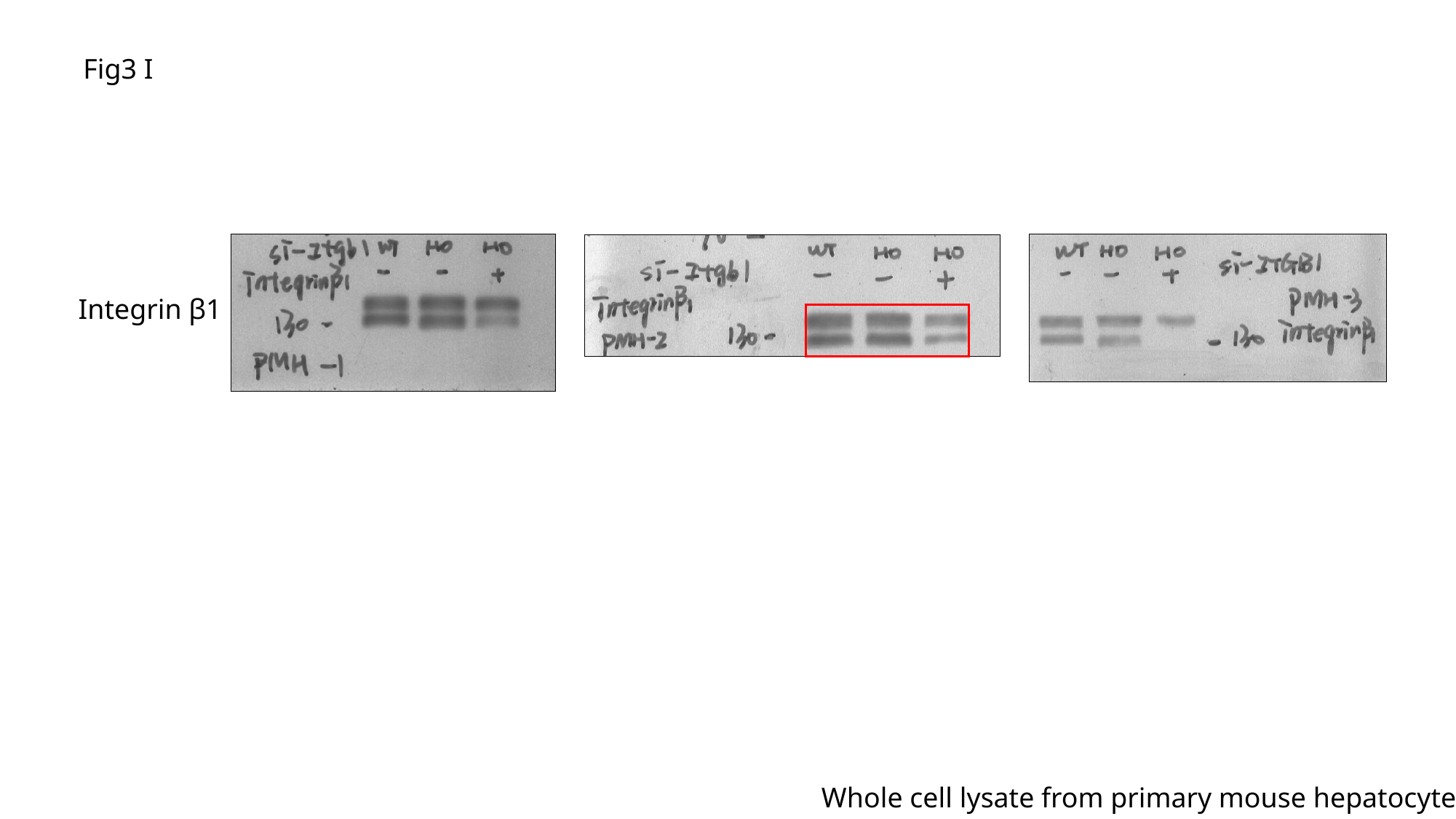

Fig3 I
Integrin β1
Whole cell lysate from primary mouse hepatocytes

## Slide 16
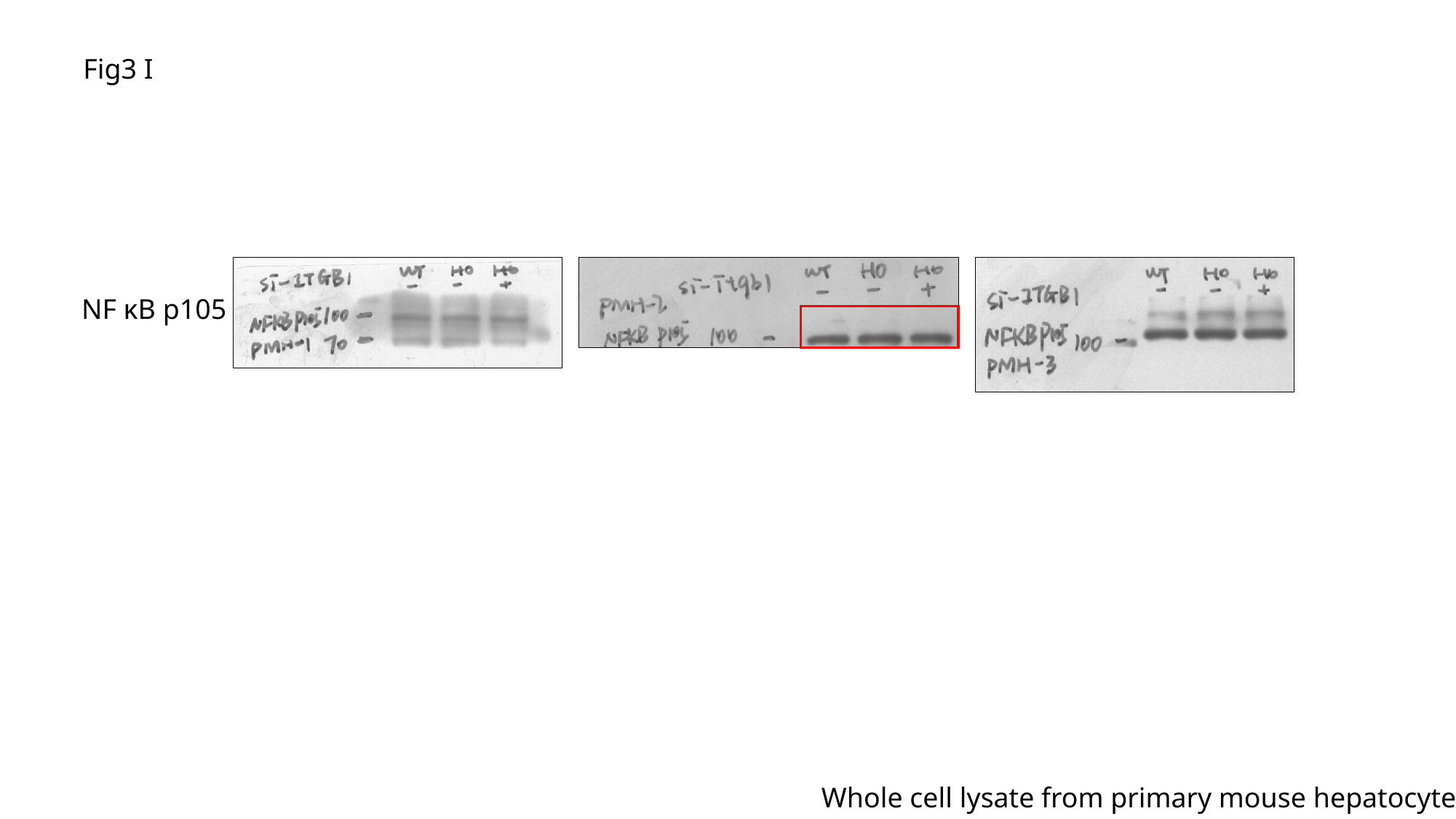

Fig3 I
NF κB p105
Whole cell lysate from primary mouse hepatocytes

## Slide 17
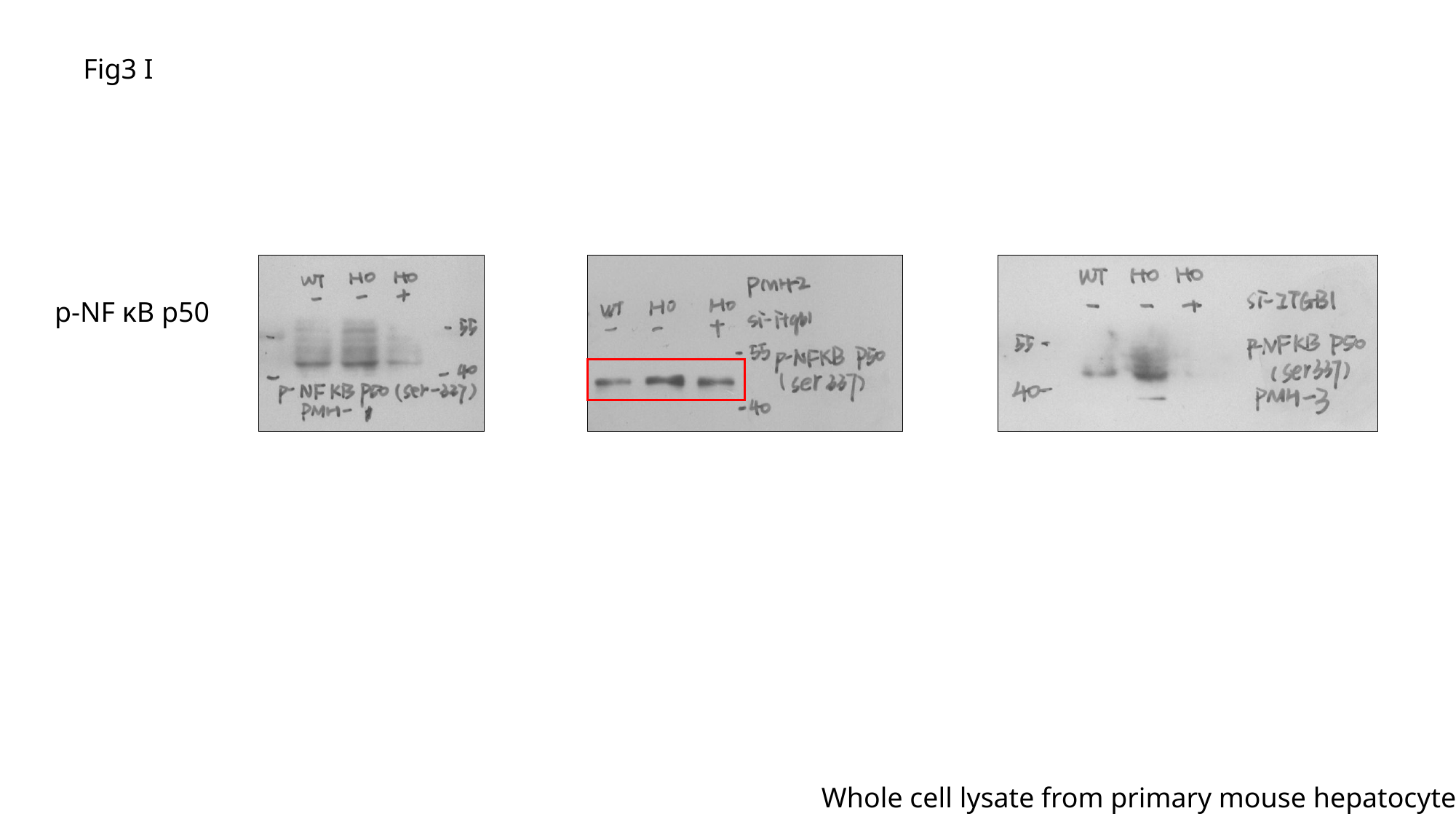

Fig3 I
p-NF κB p50
Whole cell lysate from primary mouse hepatocytes

## Slide 18
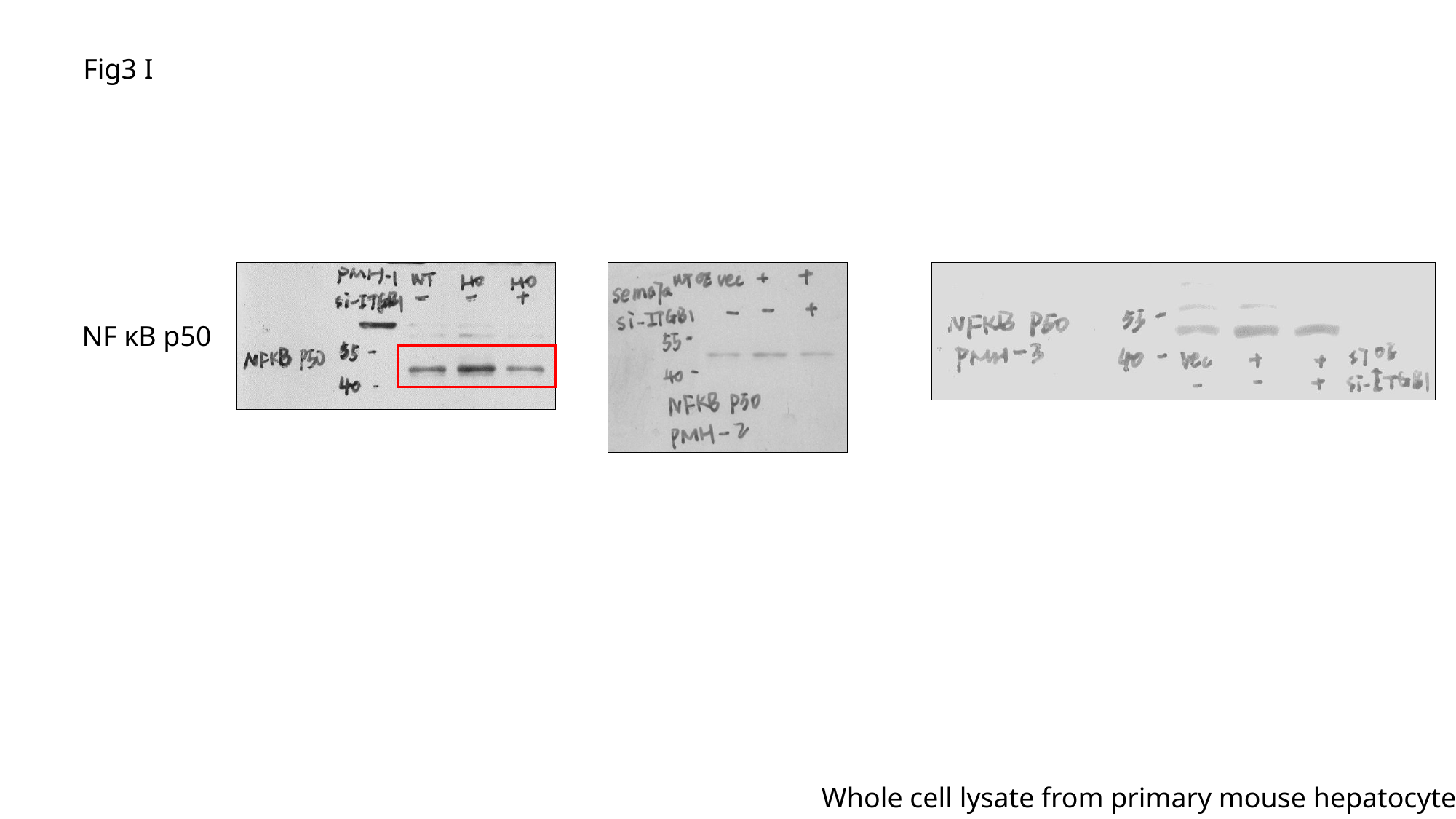

Fig3 I
NF κB p50
Whole cell lysate from primary mouse hepatocytes

## Slide 19
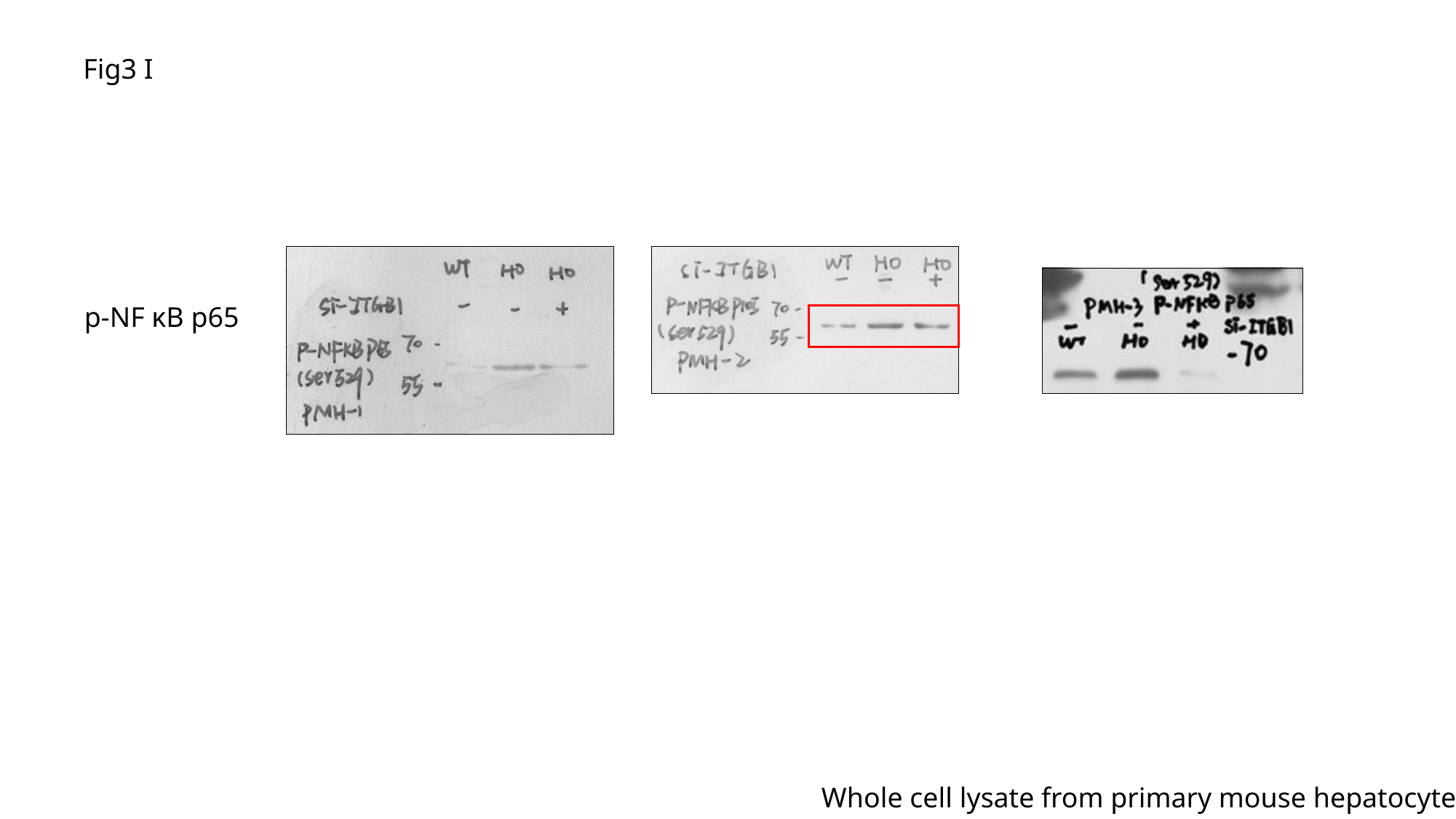

Fig3 I
p-NF κB p65
Whole cell lysate from primary mouse hepatocytes

## Slide 20
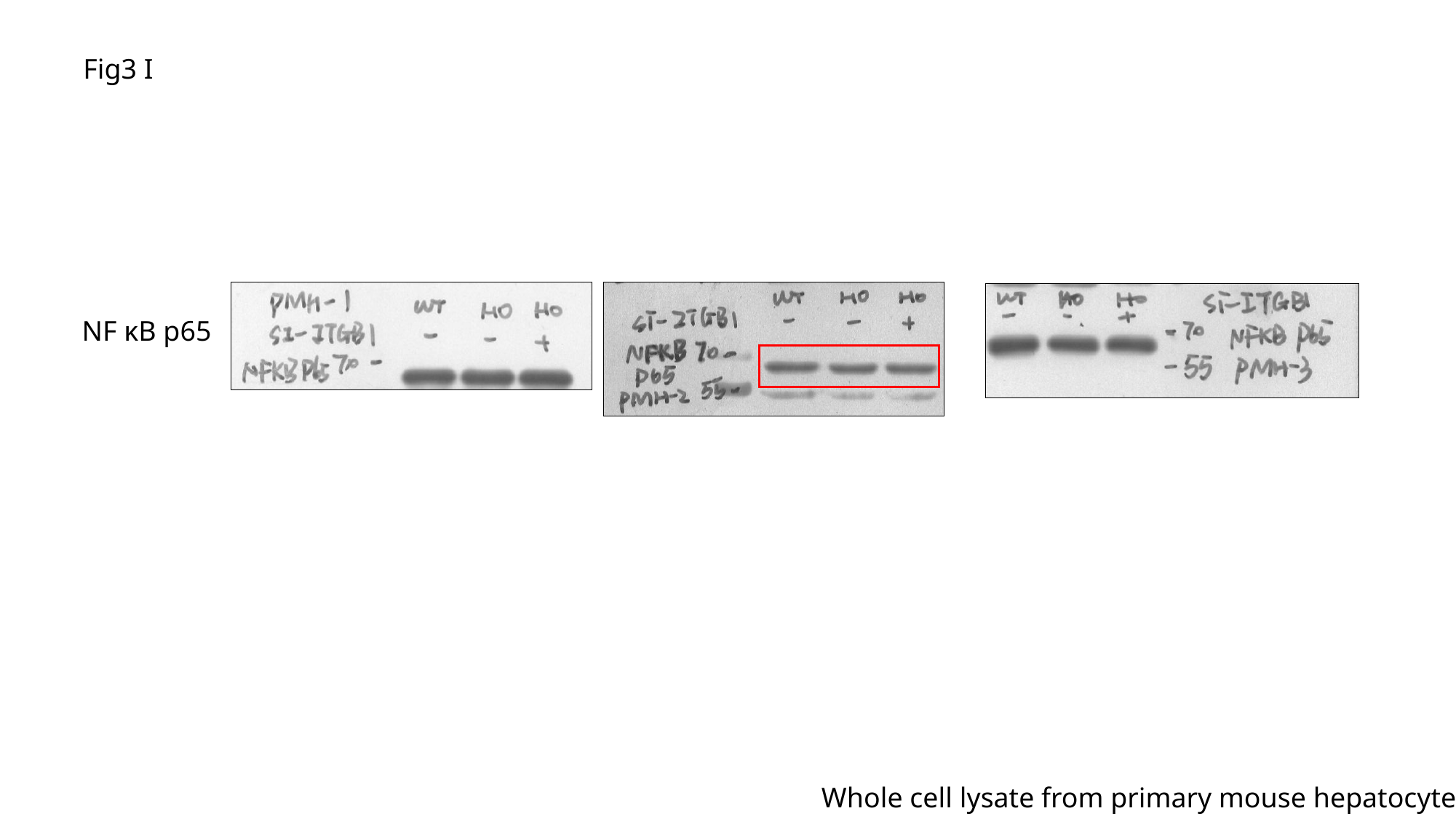

Fig3 I
NF κB p65
Whole cell lysate from primary mouse hepatocytes

## Slide 21
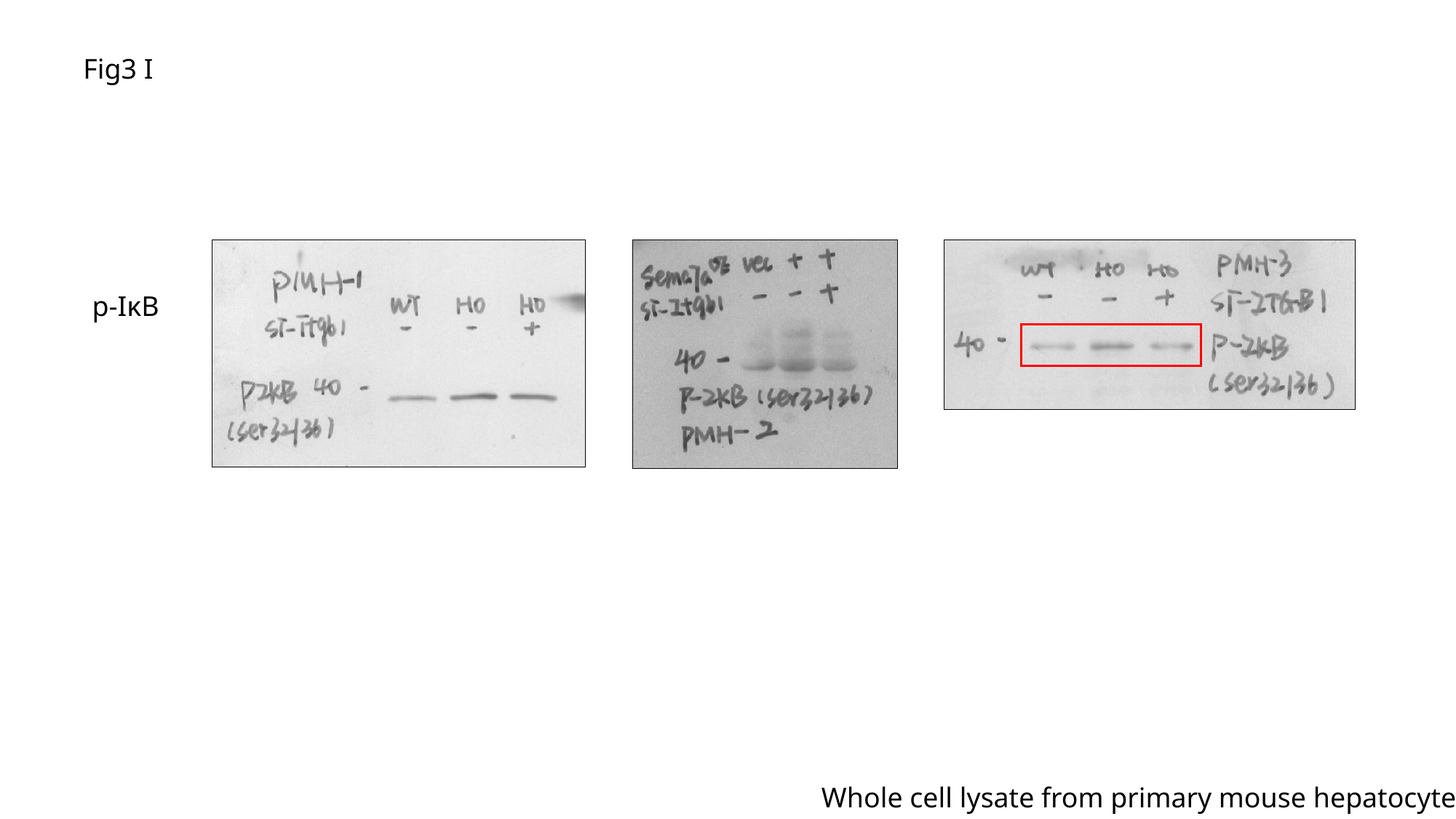

Fig3 I
p-IκB
Whole cell lysate from primary mouse hepatocytes

## Slide 22
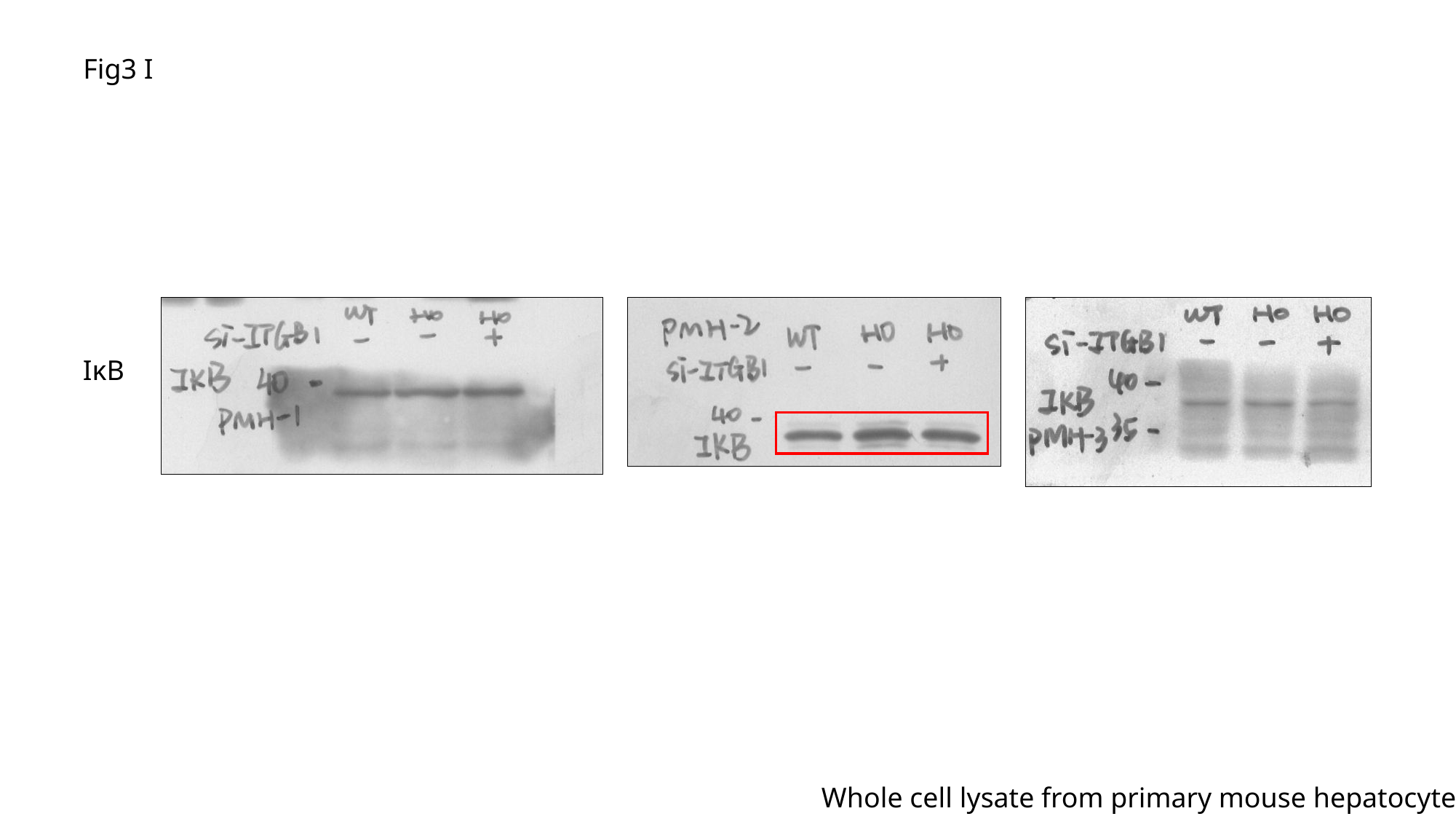

Fig3 I
IκB
Whole cell lysate from primary mouse hepatocytes

## Slide 23
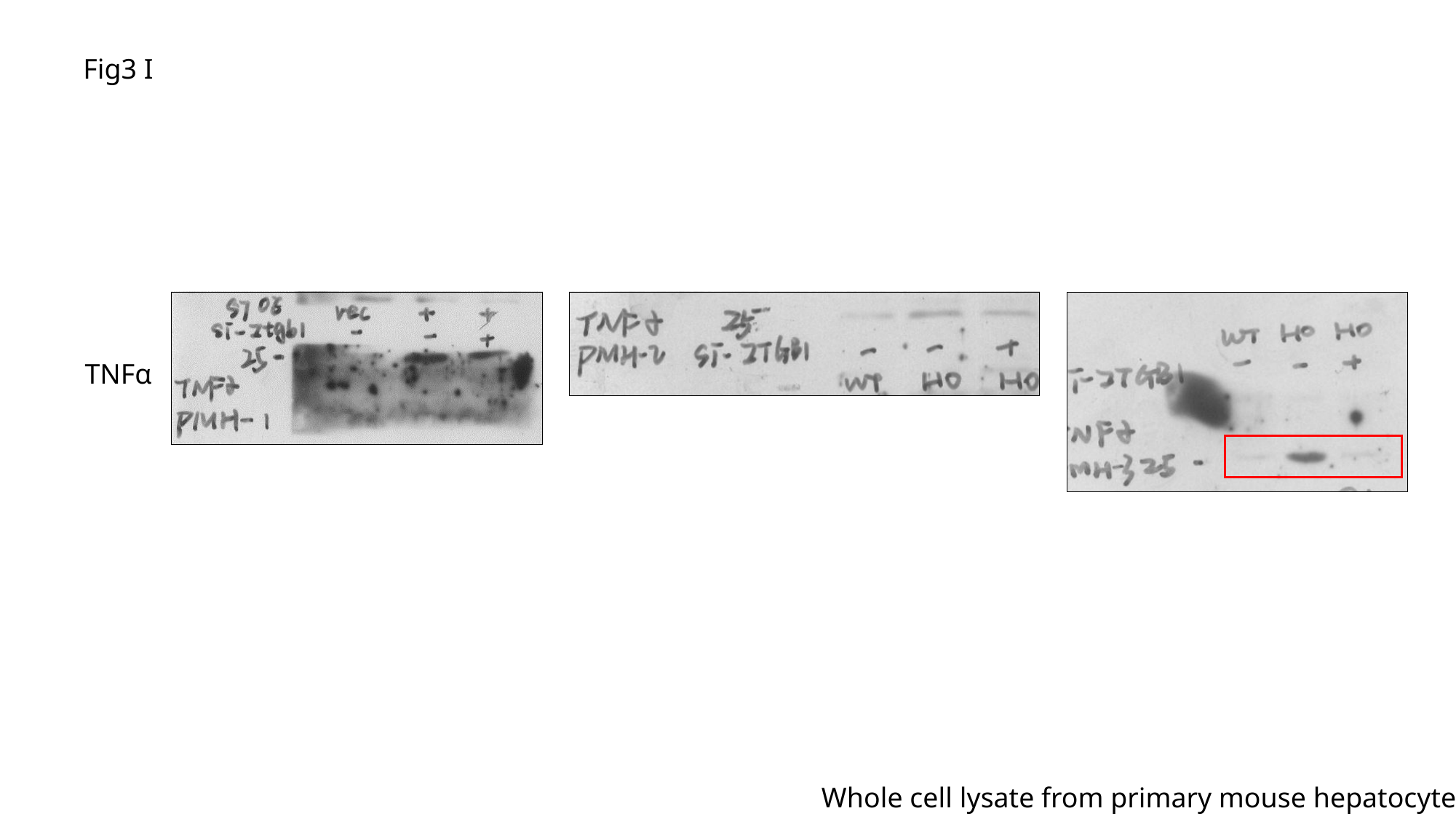

Fig3 I
TNFα
Whole cell lysate from primary mouse hepatocytes

## Slide 24
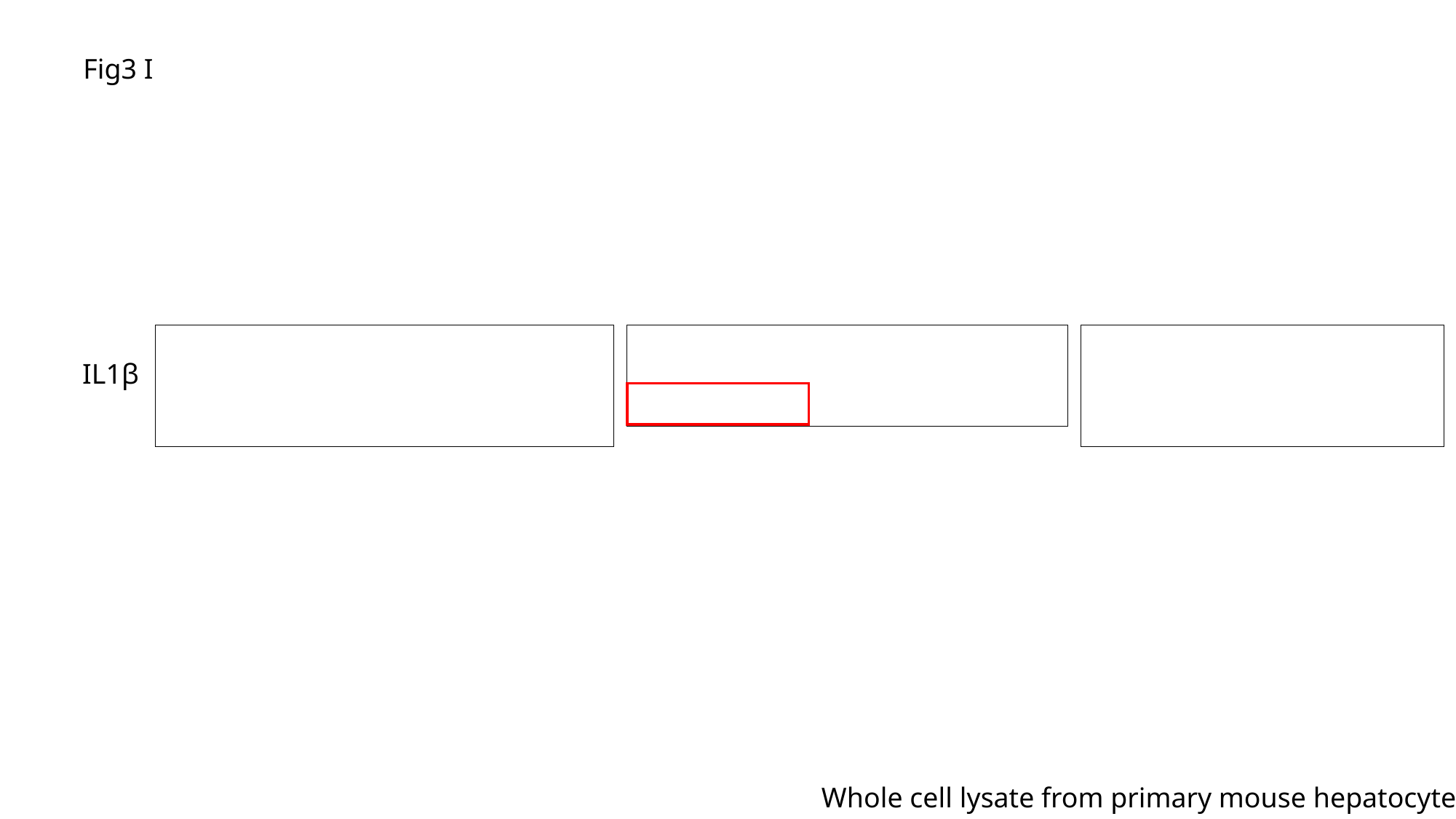

Fig3 I
IL1β
Whole cell lysate from primary mouse hepatocytes

## Slide 25
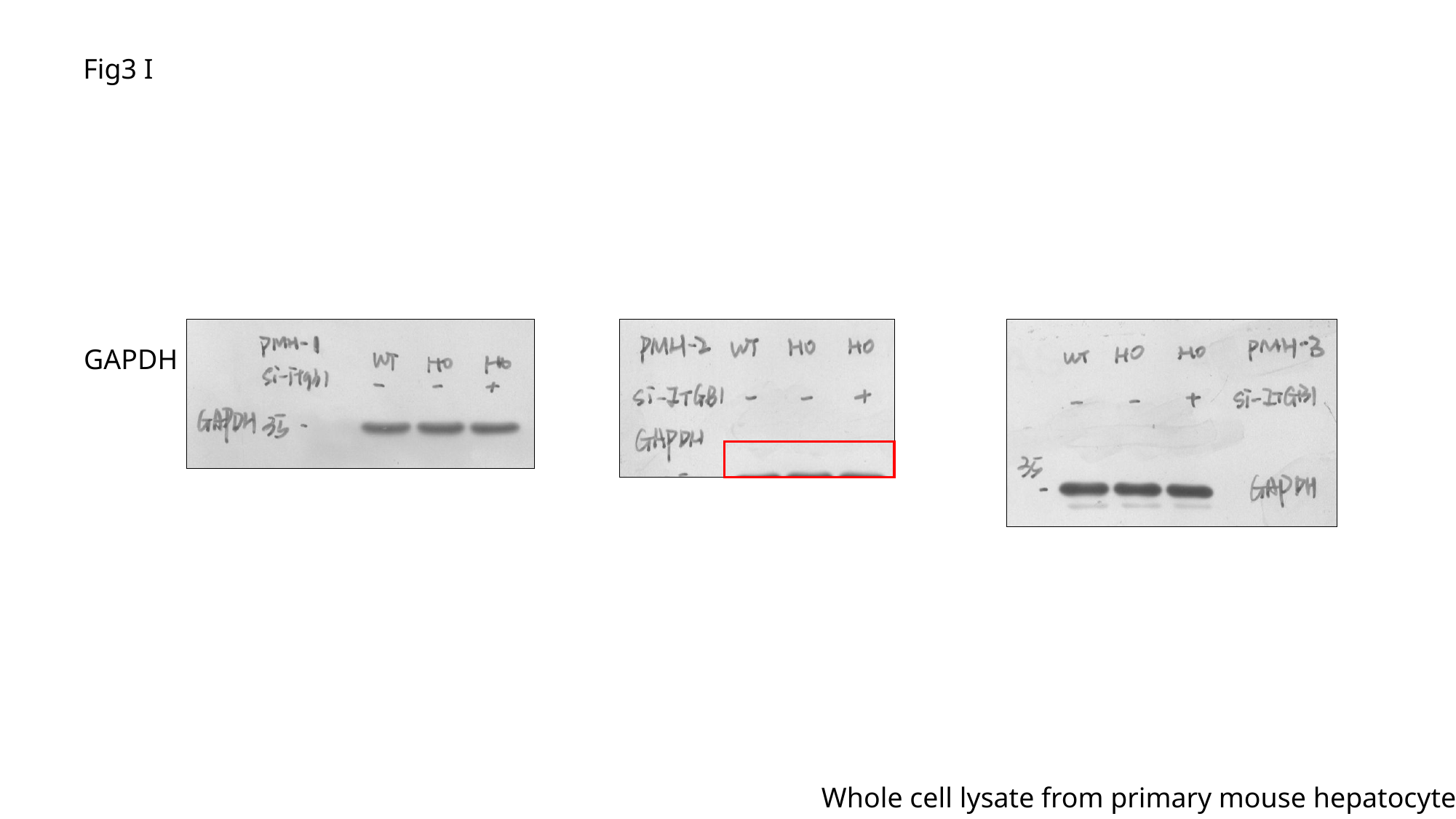

Fig3 I
GAPDH
Whole cell lysate from primary mouse hepatocytes

## Slide 26
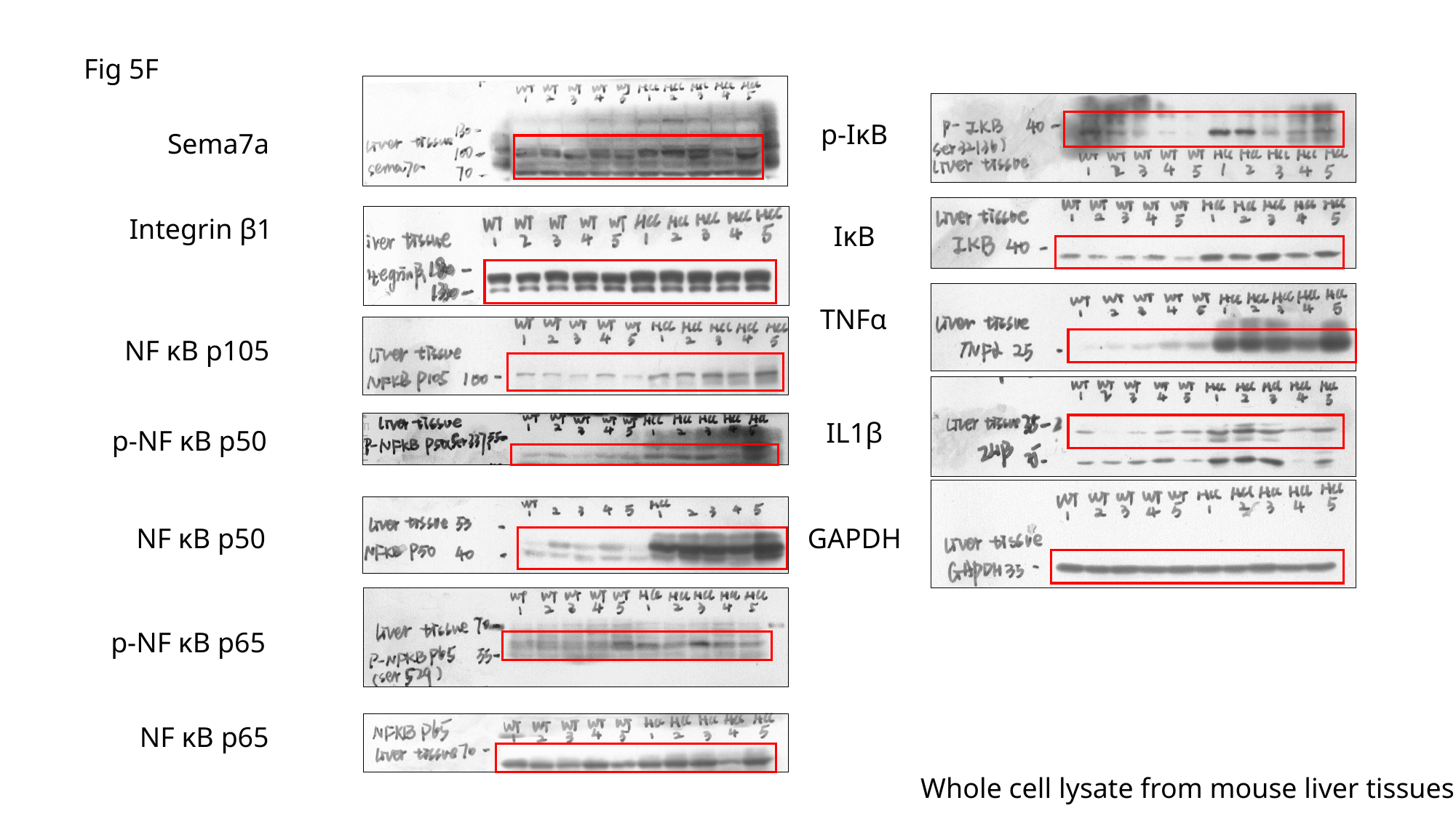

Fig 5F
p-IκB
Sema7a
Integrin β1
IκB
TNFα
NF κB p105
IL1β
p-NF κB p50
NF κB p50
GAPDH
p-NF κB p65
NF κB p65
Whole cell lysate from mouse liver tissues

## Slide 27
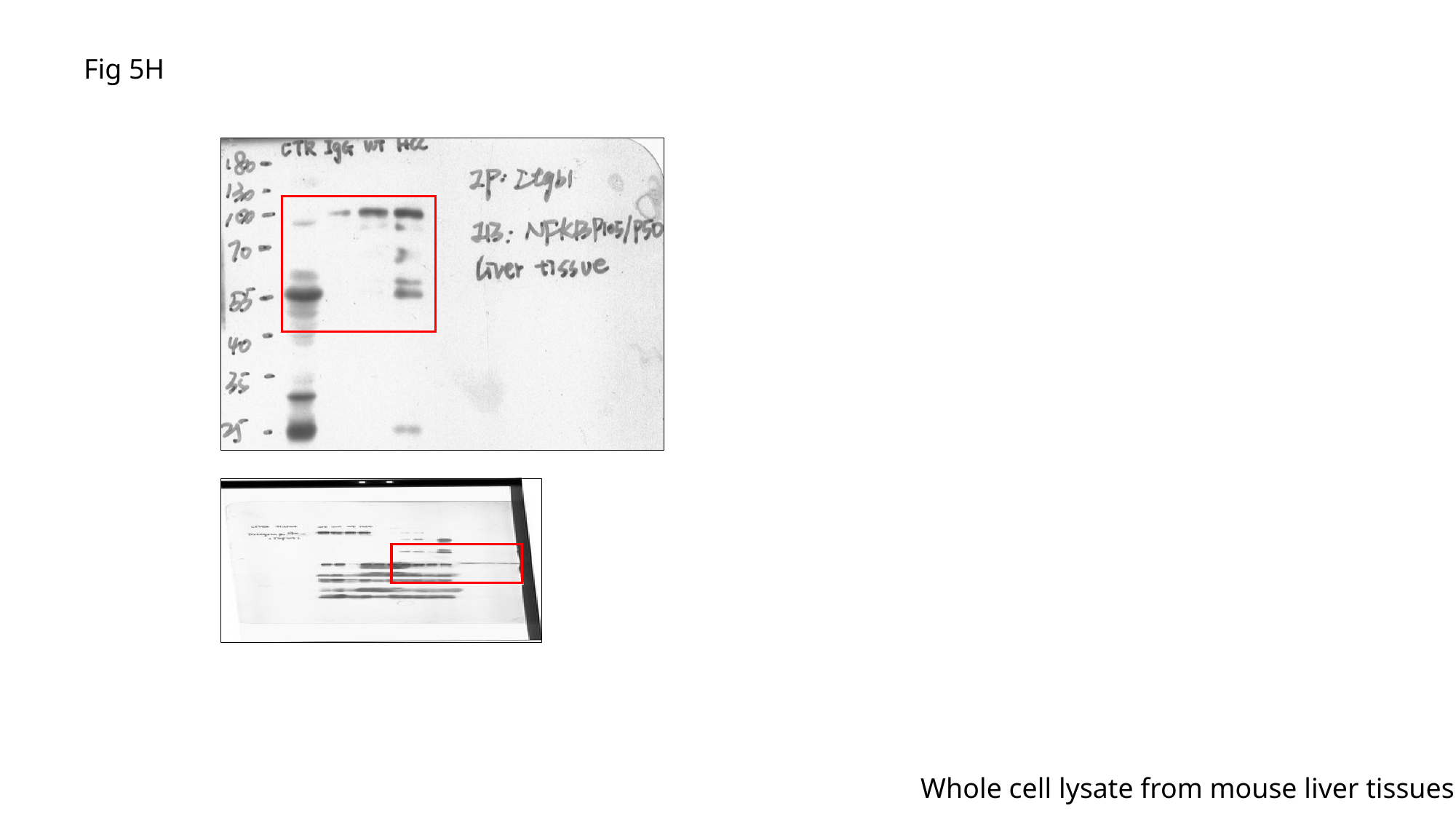

Fig 5H
Whole cell lysate from mouse liver tissues

## Slide 28
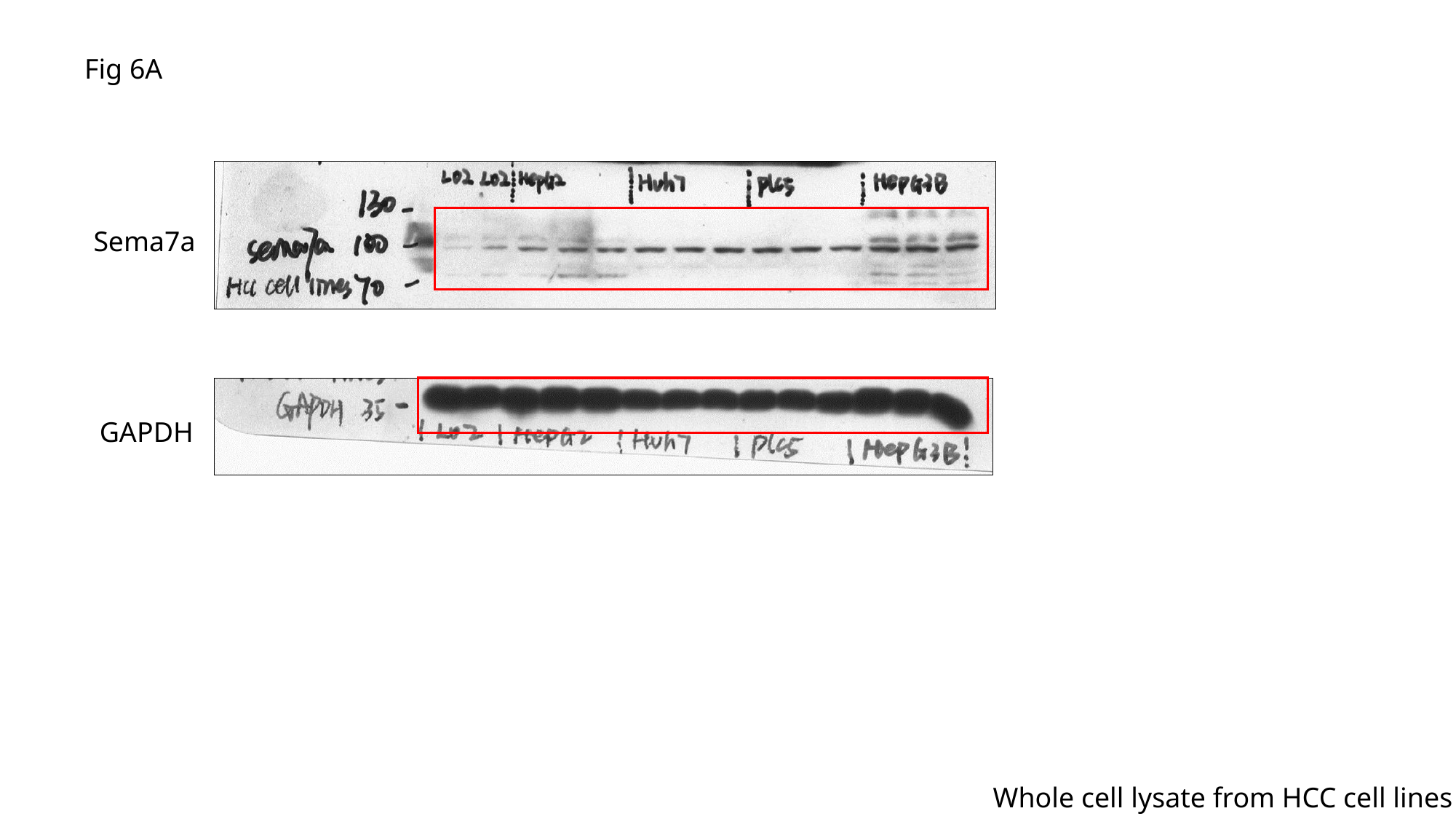

Fig 6A
Sema7a
GAPDH
Whole cell lysate from HCC cell lines

## Slide 29
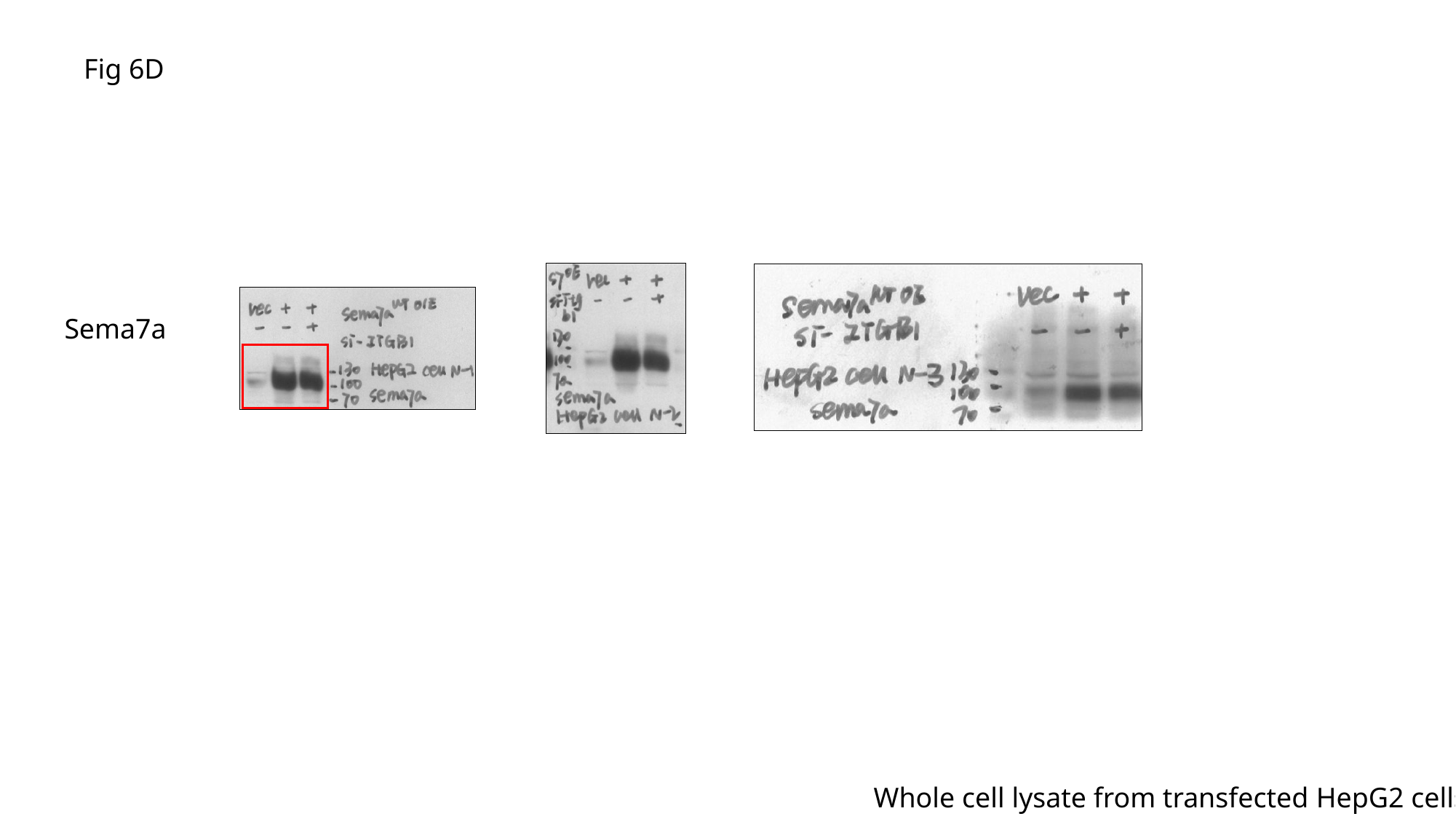

Fig 6D
Sema7a
Whole cell lysate from transfected HepG2 cells

## Slide 30
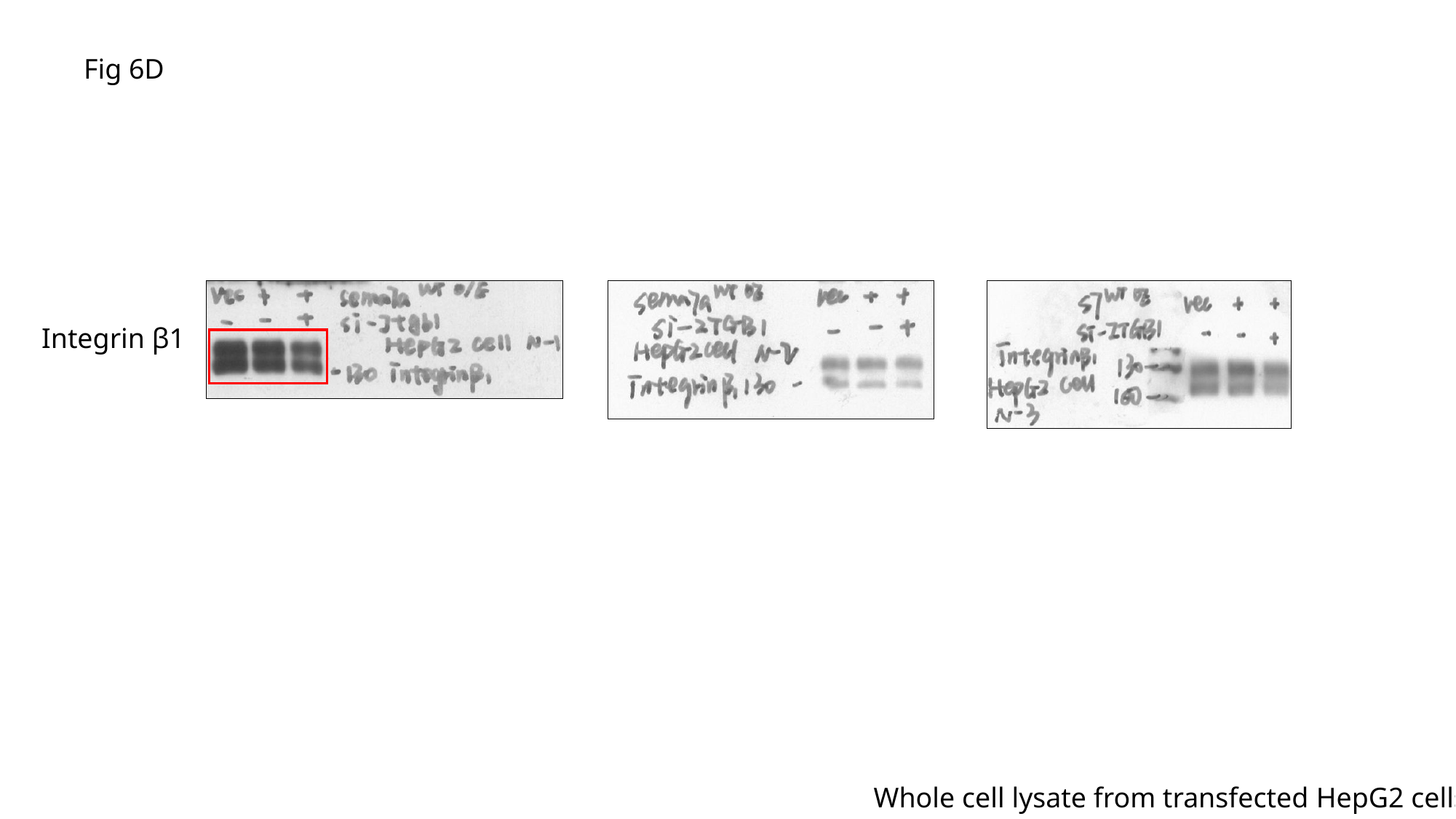

Fig 6D
Integrin β1
Whole cell lysate from transfected HepG2 cells

## Slide 31
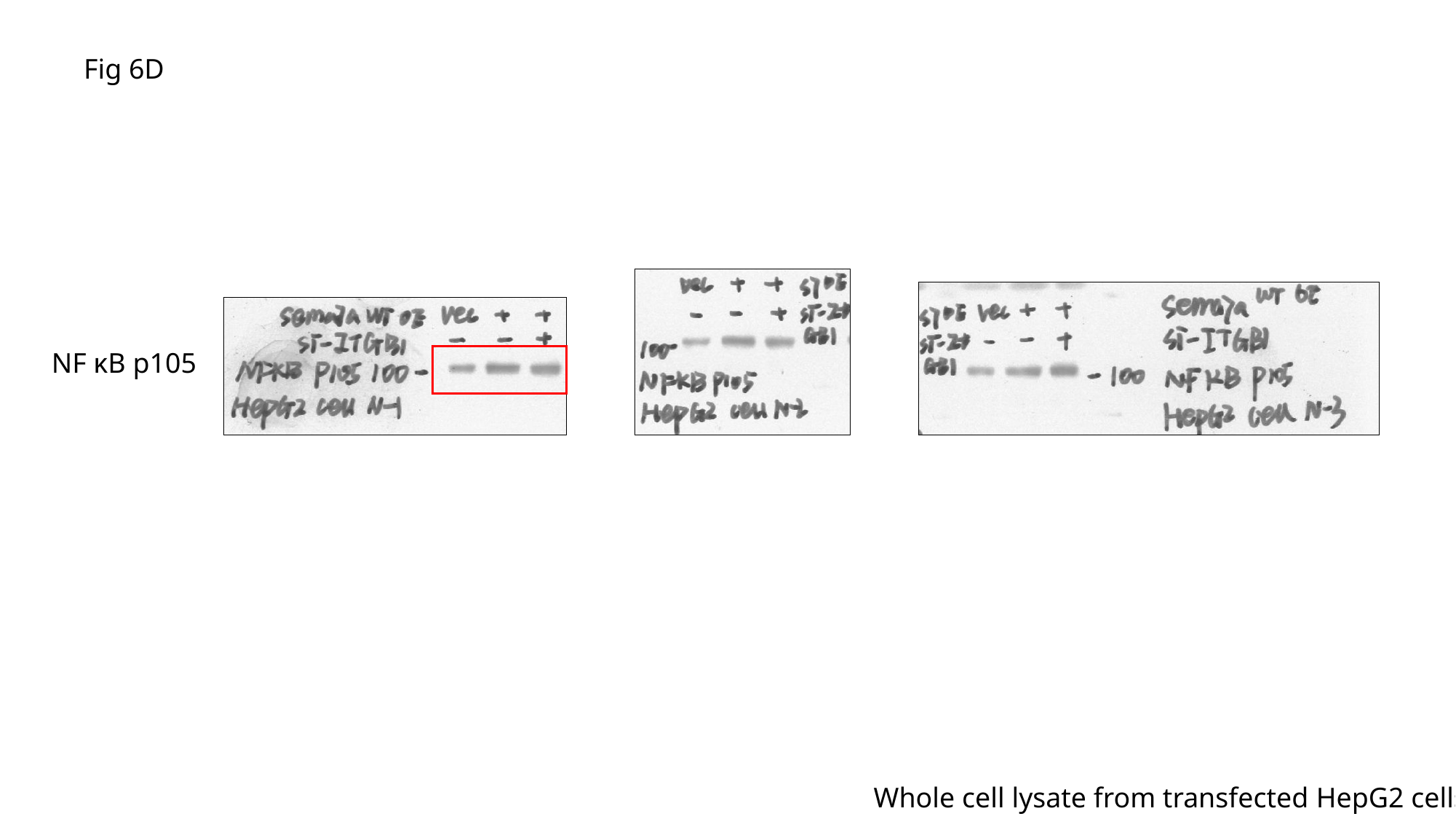

Fig 6D
NF κB p105
Whole cell lysate from transfected HepG2 cells

## Slide 32
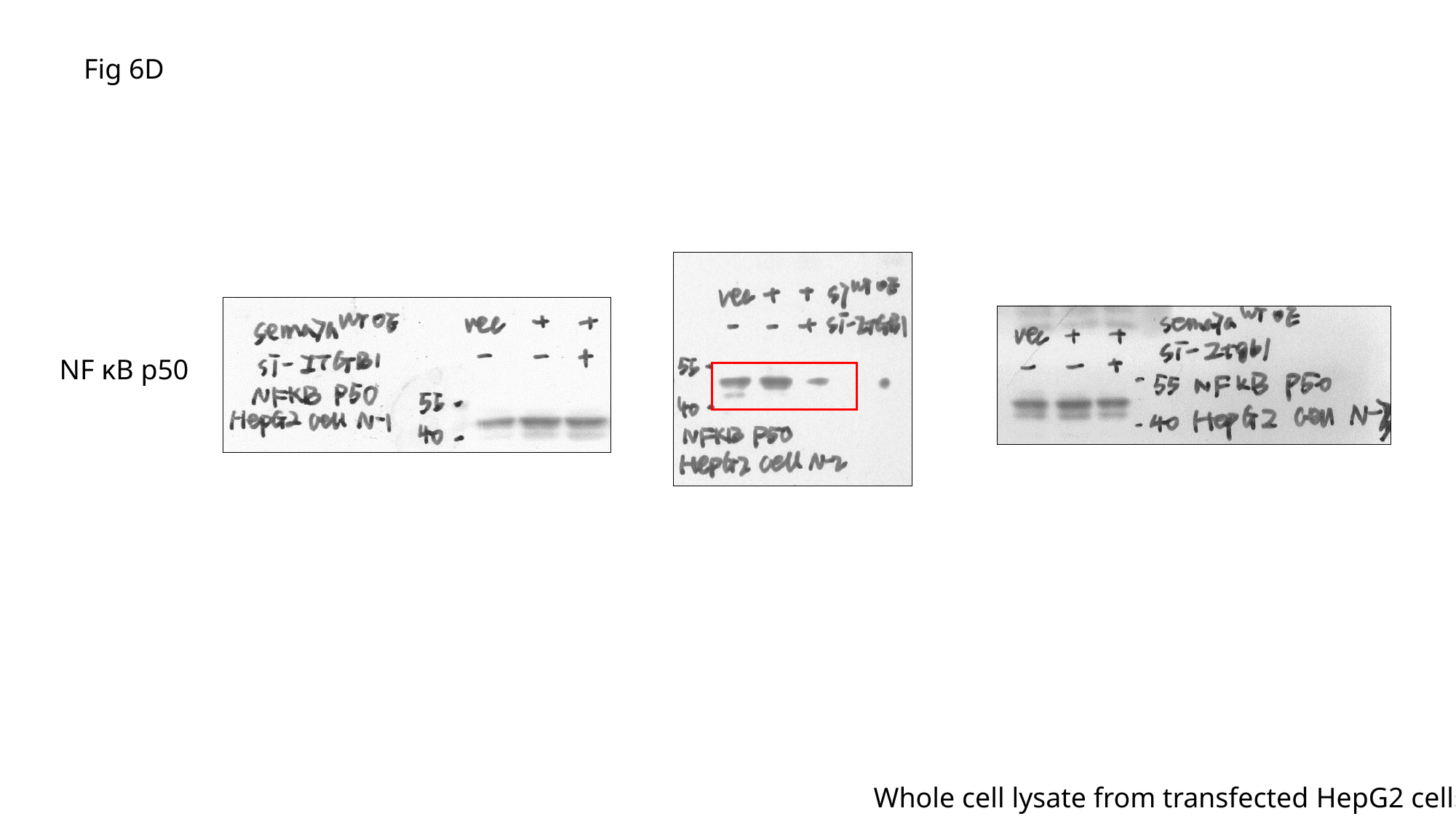

Fig 6D
NF κB p50
Whole cell lysate from transfected HepG2 cells

## Slide 33
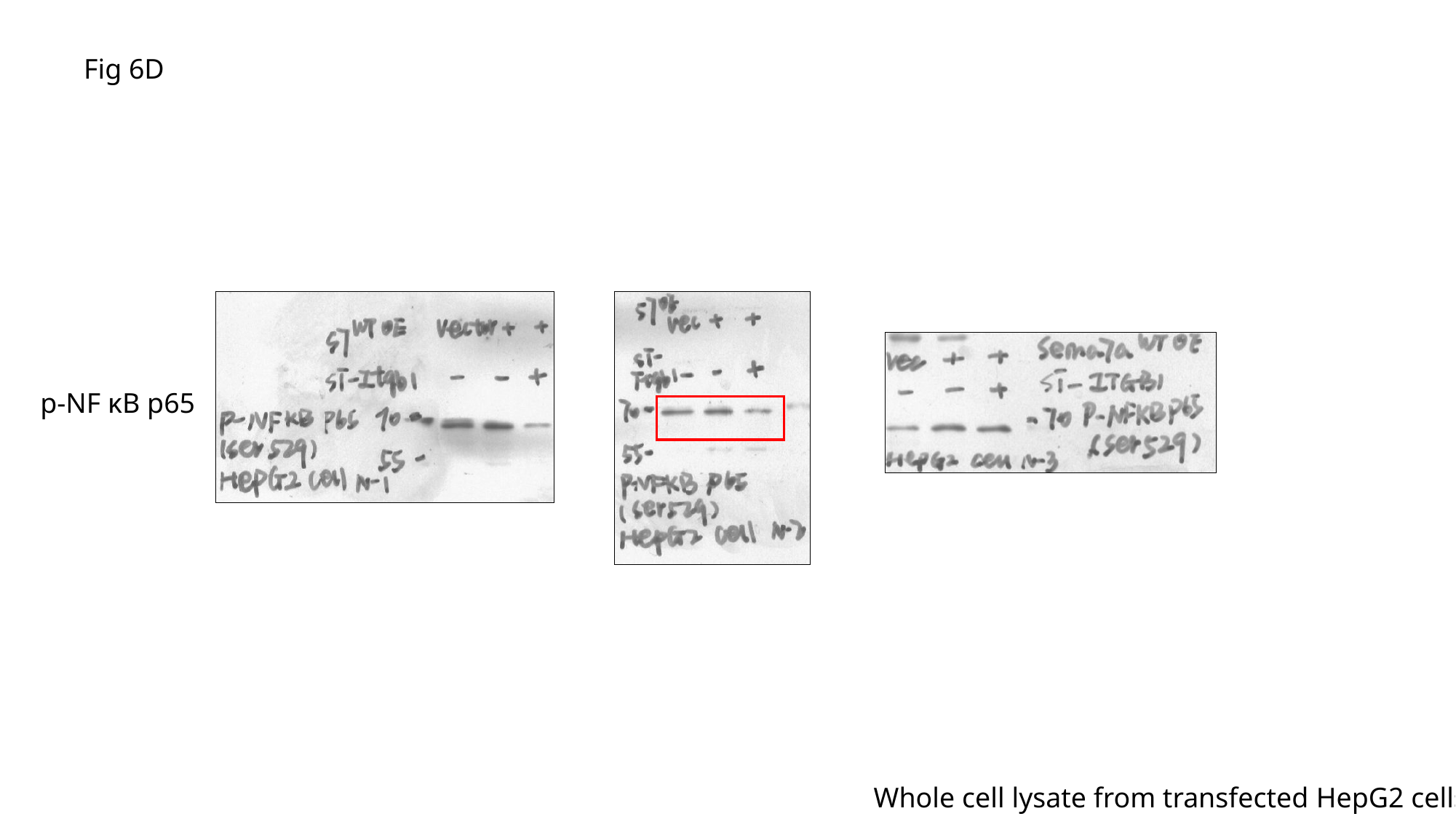

Fig 6D
p-NF κB p65
Whole cell lysate from transfected HepG2 cells

## Slide 34
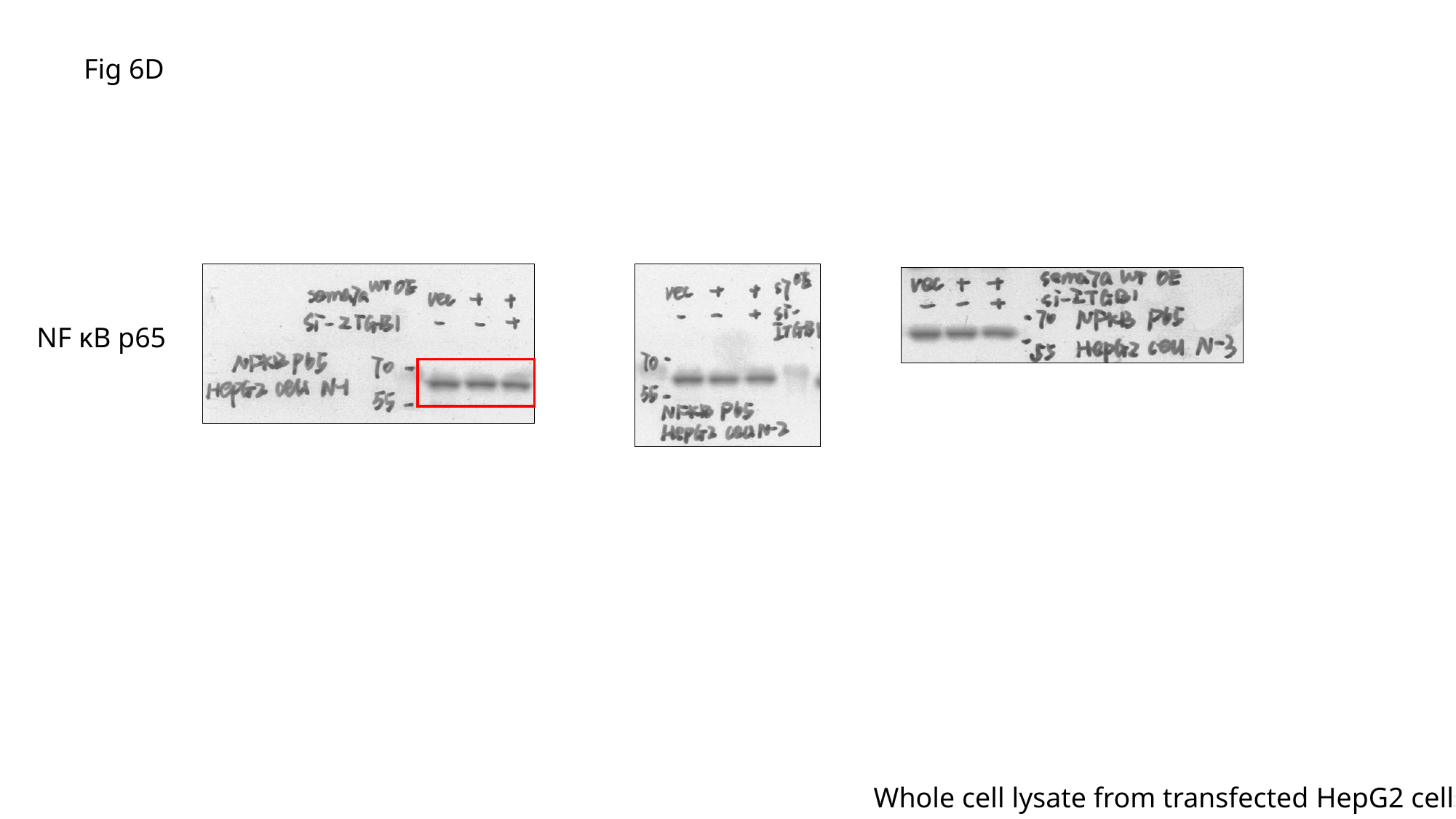

Fig 6D
NF κB p65
Whole cell lysate from transfected HepG2 cells

## Slide 35
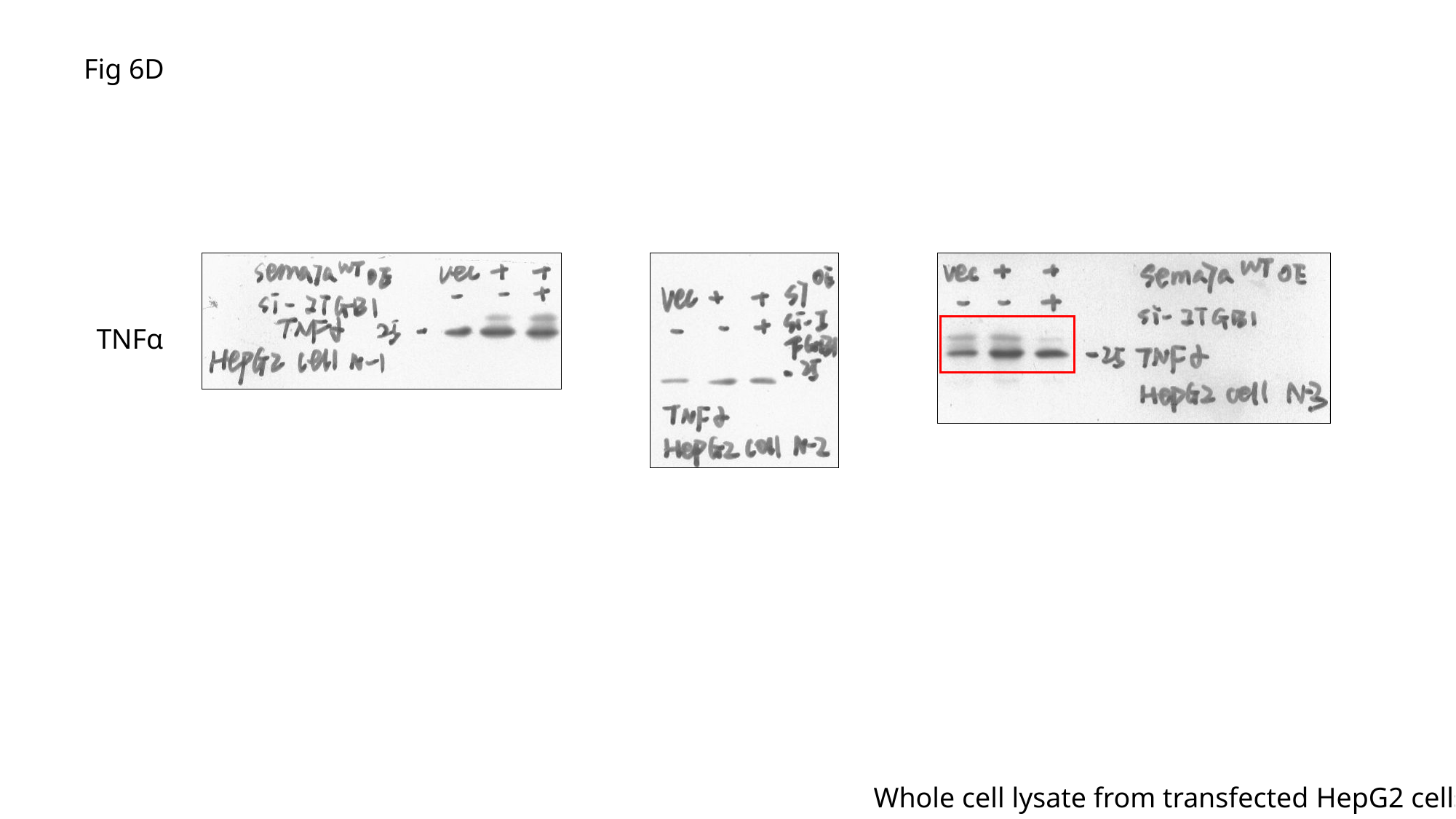

Fig 6D
TNFα
Whole cell lysate from transfected HepG2 cells

## Slide 36
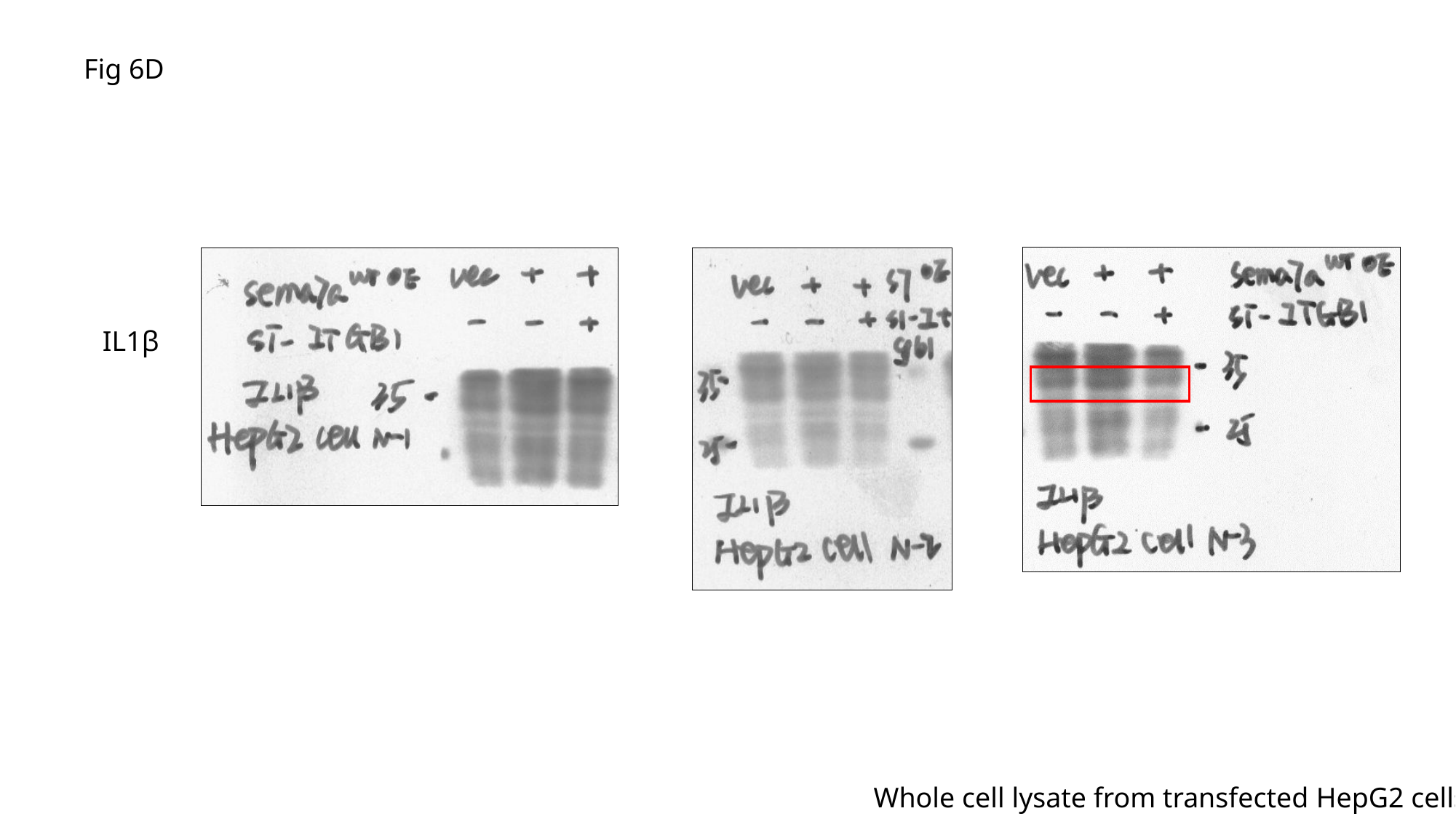

Fig 6D
IL1β
Whole cell lysate from transfected HepG2 cells

## Slide 37
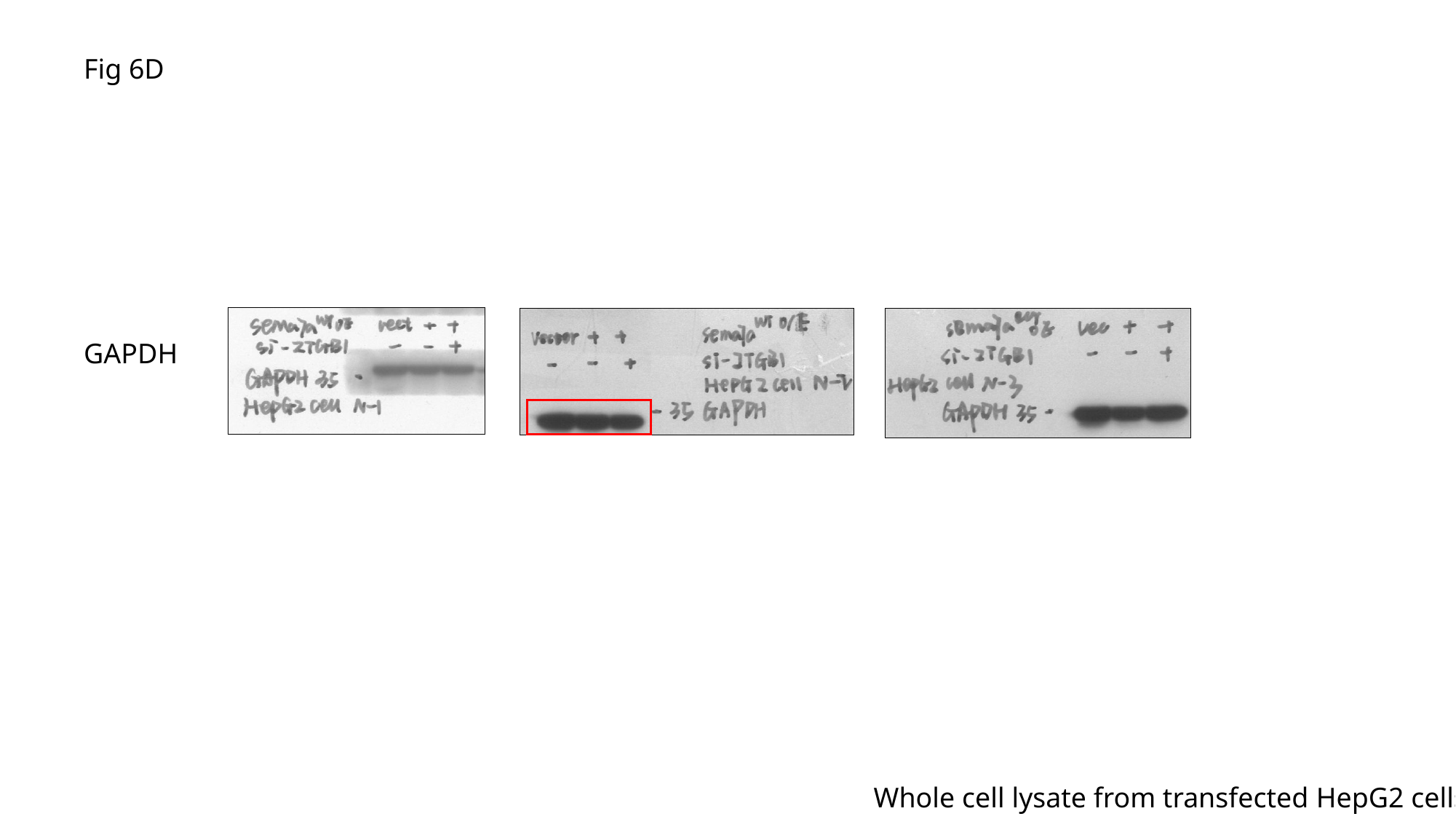

Fig 6D
GAPDH
Whole cell lysate from transfected HepG2 cells
